# Role of the heat shock transcription factor, Hsf1, in a major fungal pathogen that is obligately associated with warm-blooded animals

**DOI:** 10.1111/j.1365-2958.2009.06883.x

**Published:** 2009-10-08

**Authors:** Susan Nicholls, Michelle D Leach, Claire L Priest, Alistair J P Brown

**Affiliations:** Aberdeen Fungal Group, School of Medical Sciences, University of Aberdeen, Institute of Medical SciencesAberdeen AB25 2ZD, UK

## Abstract

All organisms have evolved mechanisms that protect them against environmental stress. The major fungal pathogen of humans, *Candida albicans*, has evolved robust stress responses that protect it against human immune defences and promote its pathogenicity. However, *C. albicans* is unlikely to be exposed to heat shock as it is obligatorily associated with warm-blooded animals. Therefore, we examined the role of the heat shock transcription factor (Hsf1) in this pathogen. We show that *C. albicans* expresses an evolutionarily conserved Hsf1 (orf19.4775) that is phosphorylated in response to heat shock, induces transcription via the heat shock element (HSE), contributes to the global transcriptional response to heat shock, and is essential for viability. Why has Hsf1 been conserved in this obligate animal saprophyte? We reasoned that Hsf1 might contribute to medically relevant stress responses. However, this is not the case, as an Hsf1-specific *HSE-lacZ* reporter is not activated by oxidative, osmotic, weak acid or pH stress. Rather, Hsf1 is required for the expression of essential chaperones in the absence of heat shock (e.g. Hsp104, Hsp90, Hsp70). Furthermore, Hsf1 regulates the expression of HSE-containing genes in response to growth temperature in *C. albicans*. Therefore, the main role of Hsf1 in this pathogen might be the homeostatic modulation of chaperone levels in response to growth temperature, rather than the activation of acute responses to sudden thermal transitions.

## Introduction

Organisms exist in constantly changing and complex environments where they are subject to wide-ranging perturbations that are often perceived as stresses. For this reason organisms have evolved stress responses that promote their survival in these dynamic environments. In particular, the heat shock response protects cells against sudden changes in temperature by inducing the expression of heat shock proteins (HSPs) that protect proteins during thermal stress and facilitate the repair or degradation of damaged proteins (Panaretou and Zhai, 2008). This response is highly conserved across the eukaryotic kingdom from the fungi to plants and animals.

In the yeast *Saccharomyces cerevisiae* the heat shock response is regulated by the heat shock transcription factor (Hsf1), which activates heat shock genes (*HSP*s) via canonical heat shock elements (HSEs) in their promoters. Hsf1 is essential for viability, binding to the HSE DNA sequence as a homotrimer even in the absence of stress (Jakobsen and Pelham, 1988; Wiederrecht *et al*., 1988; Giardina and Lis, 1995). Following heat shock, *S. cerevisiae* Hsf1 becomes hyperphosphorylated leading to the activation of *HSP* gene induction (Sorger and Pelham, 1987; Gallo *et al*., 1993). Hsf1 also responds to oxidative and heavy metal stresses in *S. cerevisiae* (Sewell *et al*., 1995; Liu and Thiele, 1996). Functional homologues of Hsf1 are conserved in metazoans (Morimoto, 1998; Pirkkala *et al*., 2001; Voellmy, 2004). These homologues execute equivalent roles to ScHsf1, but they can be activated by stress through alternative post-translational mechanisms (Hietakangas *et al*., 2003).

Stress responses appear to have evolved rapidly and in a niche-specific fashion in the fungal kingdom, as fungal species generally display stress phenotypes that reflect their environmental niches rather than their phylogenetic relatedness (Krantz *et al*., 2006; Nikolaou *et al*., 2009). For example, fungal pathogens of humans such as *Candida albicans*, *Candida glabrata* and *Aspergillus fumigatus* are more resistant to oxidative stresses than evolutionarily related benign species such as *Debaryomyces hansenii* and *Aspergillus nidulans* (Nikolaou *et al*., 2009). This is significant because these robust oxidative stresses responses help to protect these pathogens against the immune defences of their host thereby contributing to their virulence (Wysong *et al*., 1998; Hwang *et al*., 2002; Martchenko *et al*., 2004; Fradin *et al*., 2005).

*Candida albicans* is a major fungal pathogen of humans. It causes frequent mucosal infections in otherwise healthy individuals (thrush), and life-threatening systemic infections in immunocompromised patients (Odds, 1988; Calderone, 2002). *C. albicans* appears well adapted to its human host, existing as a commensal in the microbial flora of the oral and gastrointestinal tracts in nearly half of individuals (Odds, 1984). While the vast majority of isolates are from clinical specimens, *C. albicans* has also been isolated from a variety of animal hosts. These include domesticated mammals (e.g. cats, dogs, pigs, sheep), wild mammals and marsupials (e.g. monkeys, bats, rodents, kangaroos) and birds (e.g. chickens, pigeons, parrots, seagulls) (Odds, 1988). These animal isolates appear to have diverged slightly from human isolates, but not to the extent that they have become genetically separated (Jacobsen *et al*., 2008). *C. albicans* has been recovered from environmental samples such as plants, soil, lakes, sewage and hospital laundry (Do Carmo-Sousa, 1969; Gentles and La Touche, 1969; Barnett *et al*., 1983). However, the isolation of *C. albicans* from the environment has been largely restricted to areas that were likely to have been contaminated by humans or animals (reviewed by Odds, 1988). Therefore, in contrast to other fungal pathogens such as *A. fumigatus* and *Cryptococcus neoformans*, which occupy defined environmental niches as part of their life cycles, *C. albicans* is considered to be an obligatory animal saprophyte (Do Carmo-Sousa, 1969; Odds, 1988).

Numerous observations indicate that the stress responses of *C. albicans* have evolved to promote survival in animal hosts, and that these stress responses have diverged from those in benign model yeasts such as *S. cerevisiae* and *Schizosaccharomyces pombe*. For example, *C. albicans* is considerably more resistant to oxidative stresses than *S. cerevisiae* and *S. pombe* (Jamieson *et al*., 1996; Quinn and Brown, 2007; Nikolaou *et al*., 2009), although these responses are still dependent upon an evolutionarily conserved AP-1-like transcription factor, Cap1 (Alarco and Raymond, 1999). Osmotic stress responses in *C. albicans* are dependent upon the conserved stress-activated protein kinase, Hog1 (San Jose *et al*., 1996). However, the upstream signalling mechanisms that activate the Hog1 MAP kinase module have diverged in *C. albicans* compared with *S. cerevisiae* (Roman *et al*., 2005; Cheetham *et al*., 2007). Furthermore, while Hog1 primarily mediates responses to osmotic stress in *S. cerevisiae*, in *C. albicans* Hog1 is essential for responses to a broad range of stresses and also contributes to the virulence of this pathogen (Alonso-Monge *et al*., 1999; 2003; Smith *et al*., 2004). Even the core environmental stress response has diverged significantly in *C. albicans* compared with the corresponding responses in *S. cerevisiae* and *S. pombe* (Gasch *et al*., 2000; Causton *et al*., 2001; Chen *et al*., 2003; Enjalbert *et al*., 2003; 2006). While the transcription factors Msn2 and Msn4 play central roles in the activation of the environmental stress response in *S. cerevisiae* (Gasch *et al*., 2000; Causton *et al*., 2001), this is not the case in *C. albicans* where the cellular roles of Msn2/4-like proteins have diverged (Nicholls *et al*., 2004; Ramsdale *et al*., 2008). These observations led us to consider the possibility of divergence in the heat shock response in *C. albicans*. Is there a role for the heat shock response in this obligate animal saprophyte? Even in febrile patients, *C. albicans* would not be exposed to the sudden temperature changes that define the heat shock response *in vitro.*

Heat shock proteins have attracted special attention in *C. albicans* because: (i) they are particularly immunogenic during *Candida* infections, (ii) the levels of anti-HSP antibodies have been associated with increased survival in patients with systemic candidosis, and (iii) anti-Hsp90 antibodies have been reported to be immunoprotective against systemic candidosis (Matthews *et al*., 1987; 1991; Swoboda *et al*., 1993; 1995; Bromuro *et al*., 1998; Burnie *et al*., 2006). *C. albicans* activates the transcription of some *HSP* orthologues in response to heat shock (Swoboda *et al*., 1995; Sandini *et al*., 2002; Enjalbert *et al*., 2003). On the basis that these *HSP* genes contain sequences related to the classical HSE in their upstream regions, it was suggested that *C. albicans HSP* genes might be regulated by a heat shock transcription factor (Hsf1) via HSEs in their promoters. However, neither Hsf1 nor HSE functionality has been characterized in *C. albicans*.

In this study we show that *C. albicans* does have an *HSF1* gene, that this gene is essential for viability, that Hsf1 activates transcription via the HSE, and that Hsf1 contributes significantly to the global transcriptional response to heat shock in *C. albicans*. Our examination of the cellular roles of Hsf1 in *C. albicans* has led us to suggest that although it has retained the capacity to act as an ON-switch in response to heat shock, the primary role of Hsf1 in the wild is to act as a thermostat that tunes the levels of essential chaperones to growth temperature. This would account for the strong conservation of ‘heat shock’ regulation in this obligatory animal saprophyte.

## Results

### The *C. albicans HSF1* locus

Our first aim was to determine whether *C. albicans* has a homologue of the *S. cerevisiae* and other metazoan *HSF1* genes*.* Bidirectional blastn searches of the *C. albicans* genome (http://www.candidagenome.org/) revealed that *orf19.4775* gene is the sequence orthologue of *S. cerevisiae HSF1.* The *C. albicans* orf19*.*4775 protein displays significant sequence similarity to *S. cerevisiae* Hsf1 (32.5% identity over the full lengths of these proteins). Furthermore this sequence similarity increases in their DNA-binding domains (71.9%), suggesting that *orf19.4775* is *C. albicans HSF1.*

The *C. albicans orf19.4775* locus was initially named as *CTA8* on the basis that an *orf19.4775* cDNA clone was identified in a one-hybrid screen for *C. albicans* sequences capable of *trans*-activation in *S. cerevisiae* (Kaiser *et al*., 1999; http://www.candidagenome.org/). This reinforces the idea that *orf19.4775* encodes a transcriptional activator. However, to our knowledge this locus has not been characterized further. We now refer to *orf19.4775* as *HSF1* because it is the sequence and functional orthologue of *S. cerevisiae HSF1* (see below)*.*

### *C. albicans HSF1* is essential for viability

*HSF1* is an essential gene in *S. cerevisiae* (Sorger and Pelham, 1988). Given that *C. albicans* is viewed as an obligate animal saprophyte, and as such would rarely be exposed to sudden changes in ambient temperature, we reasoned that *HSF1* might not be essential for viability in this pathogenic yeast. To test this we generated a conditional *HSF1* mutant using the doxycycline-regulatable *tet*_*p*_ promoter system in the *C. albicans* strain THE1 (Nakayama *et al*., 2000). *C. albicans* is constitutively diploid. Therefore, we first deleted one *HSF1* allele in this strain, and then placed the second allele under the control of the *tet*_*p*_ promoter to create the *C. albicans hsf1/tet*_*p*_*-HSF1* mutant (CLM62-1).

**Fig. 1:**
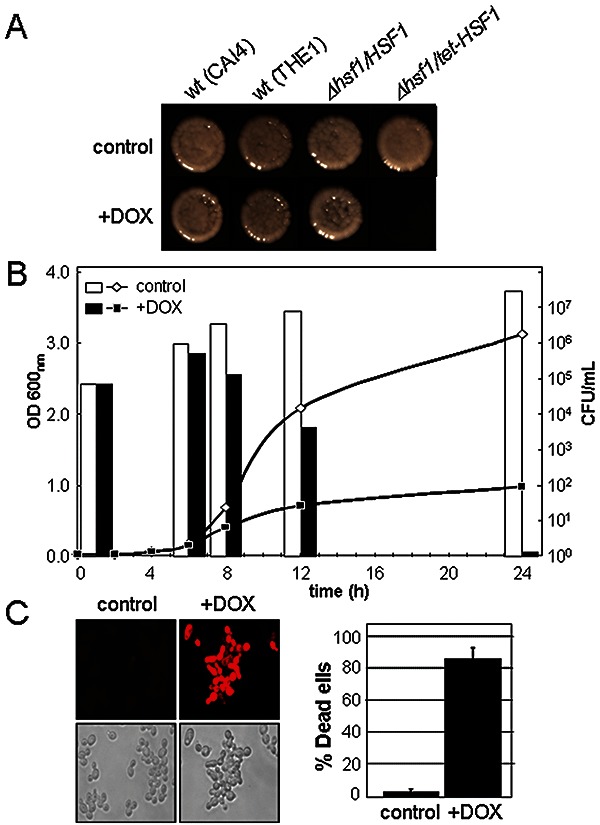
*C. albicans HSF1* is an essential gene. A. *C. albicans* cells were spotted onto YPD plates containing (+DOX) or lacking (control) 20 μg ml^−1^ doxycycline and incubated overnight at 30°C: wild type (wt, CAI4); wild type (wt, THE1); heterozygous *hsf1/HSF1* mutant (CLM60-1); conditional *hsf1/tet*_*p*_*-HSF1* mutant (CLM62-1) (Table 1). B. Growth (OD_600_ curves) and viability (cfu histograms) of *hsf1/tet*_*p*_*-HSF1* (CLM62-1) cells in the presence or absence of 20 μg ml^−1^ doxycycline added at *t* = 0: filled symbols, +DOX; open symbols, control lacking doxycycline. C. The conditional mutant (*hsf1/tet*_*p*_*-HSF1*: CLM62-1) was grown for 16 h in YPD in the presence or absence of 20 μg ml^−1^ doxycycline. Cells were then stained with propidium iodide to determine the proportion of metabolically active cells.

This conditional *hsf1/tet*_*p*_*-HSF1* mutant was grown to mid-exponential phase and plated alongside *HSF1/HSF1* (THE1) and *hsf1/HSF1* (CLM61-1) controls on yeast-peptone-dextrose (YPD) medium containing or lacking 20 μg ml^−1^ doxycycline, which downregulates the *tet*_*p*_ promoter. No significant growth was observed at 30°C for the *hsf1/tet*_*p*_*-HSF1* cells on the doxycycline-containing plates, indicating that *HSF1* is required for the growth of *C. albicans* (Fig. 1A).

These strains were also examined in liquid culture. *hsf1/tet*_*p*_*-HSF1* cells stopped growing after 6–8 h in YPD containing 20 μg ml^−1^ doxycycline at 30°C, while their growth continued in the absence of doxycycline (Fig. 1B). The viability of *hsf1/tet*_*p*_*-HSF1* cells decreased significantly after 6 h, dropping by over three orders of magnitude after 12 h. This was confirmed by propidium iodide staining, which indicated that < 85% of *hsf1/tet*_*p*_*-HSF1* cells grown in the presence of doxycycline were metabolically inactive (Fig. 1C). Therefore, *HSF1* executes an essential function(s) during the growth of *C. albicans* at normal temperatures, in addition to its predicted role(s) during stress responses.

### *HSF1* is required for the expression of HSPs in *C. albicans*

**Fig. 2:**
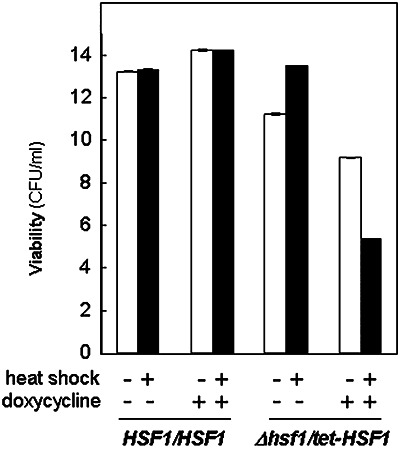
Hsf1 depletion causes heat shock sensitivity. Wild-type cells (*HSF1/HSF1*: THE1) and the conditional mutant (*hsf1/tet*_*p*_*-HSF1*: CLM62-1) were treated with 0 or 20 μg ml^−1^ doxycycline for 6 h, then subjected to a 30–45°C heat shock for 30 min, or maintained at 30°C, and cell viability assayed by plating on YPD. Means and standard deviations from triplicate experiments are shown.

We tested whether Hsf1 contributes to thermotolerance by measuring the impact of heat shock upon the viability of *hsf1/tet*_*p*_*-HSF1* cells treated with doxycycline for 6 h. At this stage their growth had ceased, but their viability remained high in the absence of a heat shock (Fig. 1B). After heat shock (30–45°C for 30 min), the viability of these doxycycline-treated *hsf1/tet*_*p*_*-HSF1* cells was reduced significantly compared with control cells not exposed to doxycycline (*P* < 0.001) (Fig. 2). Furthermore the viability of wild-type control cells was not affected by heat shock, whether exposed to doxycycline or not. Therefore, Hsf1 depletion renders *C. albicans* cells more sensitive to heat shock.

Hsf1 is known to regulate the expression of HSPs and chaperones in *S. cerevisiae* in response to heat shock (Ruis and Schuller, 1995; Morano *et al*., 1998). In *C. albicans*, the expression levels of some HSPs have been shown to be upregulated during heat stress conditions (Swoboda *et al*., 1995; Enjalbert *et al*., 2003). Therefore, to test whether Hsf1 is required for this upregulation, we examined the levels of the *HSP90*, *HSP70* and *HSP104* mRNAs by Northern blotting.

**Fig. 3:**
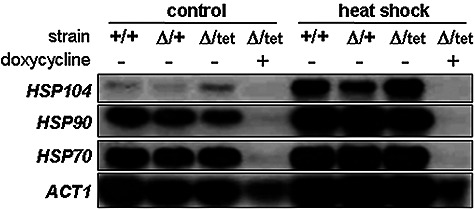
*HSF1* is required for basal expression and heat shock induction of *HSP* genes in *C. albicans*. *C. albicans* strains were grown at 30°C in YPD containing or lacking 20 μg ml^−1^ doxycycline for 6 h. Cells were subjected to a 30–45°C heat shock for 30 min, or maintained at 30°C: wild type, +/+ (THE1); heterozygous *hsf1/HSF1* mutant, Δ/+ (CLM60-1); conditional *hsf1/tet*_*p*_*-HSF1* mutant, Δ/tet (CLM62-1) (Table 1). RNA was isolated from these cells and subjected to Northern blotting. Filters were probed for the *HSP70*, *HSP90*, *HSP140* and *ACT1* mRNAs. The *HSP70* probe cross-reacted with transcripts from several *HSP70* family members: *HSP70*, *SSA2*, *SSB1*, *KAR2* and *SSCI* (not shown).

**Fig. 4:**
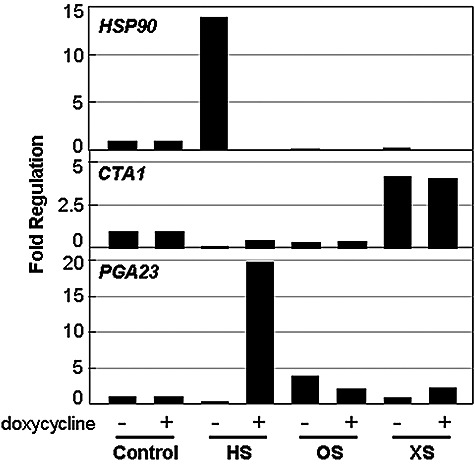
The conditional *hsf1/tet*_*p*_*-HSF1* mutant remains transcriptionally responsive to other stimuli after Hsf1 depletion. The conditional *hsf1/tet*_*p*_*-HSF1* mutant (CLM62-1) was grown at 30°C in YPD containing or lacking 20 μg ml^−1^ doxycycline for 6 h. Cells were then subjected to different stresses for 30 min: control, unstressed cells; HS, heat shock (30–45°C); OS, osmotic stress (1 M NaCl); XS, oxidative stress (5 mM H_2_O_2_). RNA was isolated from these cells, and *HSP90*, *CTA1*, *PGA23* and *ACT1* mRNA levels measured by real-time RT-PCR. mRNA levels were estimated relative to the internal *ACT1* control, and their fold regulation measured relative to the unstressed control. Similar results were obtained for two independent experiments.

RNA was extracted from wild-type cells (THE1), the heterozygote (CLM61-1) and the conditional mutant (CLM62-1) grown at 30°C or subjected to a 45°C heat shock for 30 min (Fig. 3). The *HSP90* and *HSP70* mRNAs were expressed at significant levels in all three strains even under basal conditions (at 30°C), but their expression increased further in response to the heat shock. Minimal *HSP104* expression was observed under basal conditions, but this mRNA was strongly upregulated by the heat shock (Fig. 3). No induction of these *HSP* mRNAs was observed in *hsf1/tet*_*p*_*-HSF1* cells treated with doxycycline for 6 h. At this stage most *hsf1/tet*_*p*_*-HSF1* cells remain viable (Fig. 1B). Furthermore these cells remain transcriptionally responsive to other stimuli. For example, doxycycline-treated *hsf1/tet*_*p*_*-HSF1* cells still respond to oxidative and osmotic stress by upregulating the *CTA1* and *PGA23* transcripts respectively (Fig. 4). Therefore, Hsf1 is required for the induction of the *HSP70*, *HSP90* and *HSP104* mRNAs in response to heat shock.

Hsp90 function is essential for viability in *S. cerevisiae* and is thought to be essential in *C. albicans* (Borkovich *et al*., 1989; Swoboda *et al*., 1995). Likewise, Hsp70 functions are essential in *S. cerevisiae* (Lindquist, 1986), and are presumed to be essential in *C. albicans.* As mentioned above, the *C. albicans HSP90* and *HSP70* mRNAs were expressed at significant levels under basal conditions. Interestingly, these basal expression levels were markedly reduced in doxycycline-treated *hsf1/tet*_*p*_*-HSF1* cells (Fig. 3). This indicates that Hsf1 is required for the basal expression of essential HSPs in *C. albicans*, even in the absence of heat shock.

### Genome-wide analysis of the role of Hsf1 during heat shock in *C. albicans*

**Fig. 5:**
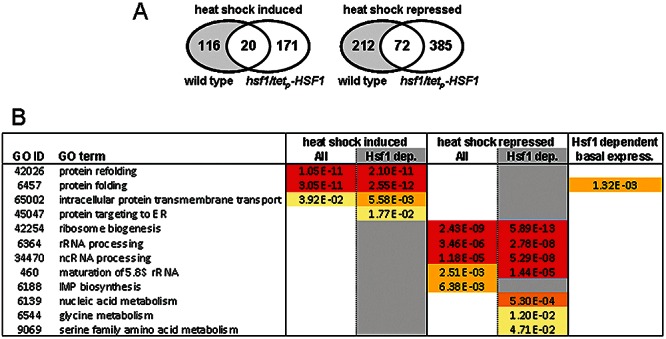
Impact of Hsf1 upon the *C. albicans* transcriptome under basal and heat shock conditions. A. Venn diagrams showing the number of *C. albicans* genes that are upregulated or downregulated in response to a 30–45°C heat shock in doxycycline-treated wild-type and *hsf1/tet*_*p*_*-HSF1* cells. Those gene subsets that were regulated in an Hsf1-dependent fashion are highlighted in grey (i.e. regulated in wild-type cells, but no longer regulated in doxycycline-treated *hsf1/tet*_*p*_*-HSF1* cells: see text). B. Gene ontology (GO) categories that were significantly enriched in specific subsets of *C. albicans* genes: All, categories that were heat shock-regulated in wild-type *C. albicans* cells; Hsf1-dependent genes (columns highlighted in grey), categories that were heat shock-regulated in wild-type cells, but not in doxycycline-treated *hsf1/tet*_*p*_*-HSF1* cells; Hsf1-dependent basal expression, categories that were downregulated in doxycycline-treated *hsf1/tet*_*p*_*-HSF1* cells in the absence of heat shock. The probabilities of the observed enrichment occurring at random are given.

As a platform for our microarray analyses of Hsf1 function, we examined the effects of a heat shock (30–45°C) upon the transcriptome of wild-type cells. To achieve this, the parental strain for our conditional *hsf1/tet*_*p*_*-HSF1* mutant (THE1; *HSF1/HSF1*) was subjected to a 30–45°C heat shock for 10 min, and this transcriptome compared to control cultures grown at 30°C. As expected, we found that *HSP* genes were strongly induced under these conditions (on average about sixfold;*Supporting information*). A total of 136 genes were reproducibly upregulated at least twofold by heat shock in wild-type *C. albicans* under our experimental conditions (Fig. 5A; *Supporting information*). This subset of heat shock-inducible genes was highly enriched in protein folding and refolding functions (Fig. 5B). Genes involved in intracellular protein transmembrane transport and protein targeting to the endoplasmic reticulum were also significantly enriched in the set of heat shock-inducible genes. Similar sets of genes are upregulated by heat shock in *S. cerevisiae* (Gasch *et al*., 2000; Causton *et al*., 2001; Hahn *et al*., 2004). Our data strengthen the view that the protection of protein folding and targeting is integral to heat shock adaptation in *C. albicans.*

Under our experimental conditions, a total of 284 genes were downregulated at least twofold by heat shock in wild-type *C. albicans* (Fig. 5A; *Supporting information*). These heat shock-inducible genes were highly enriched in ribosome biogenesis and RNA processing functions (Fig. 5B). This is consistent with observations in *S. cerevisiae* where heat shock leads to the downregulation of RNA processing functions and the disassembly of the nucleolus (Liu *et al*., 1996; Gasch *et al*., 2000; Causton *et al*., 2001). Indeed, RNA processing is particularly sensitive to heat shock in budding yeast, and specific HSPs are required to reactivate splicing (Vogel *et al*., 1995).

Malcolm Whiteway's laboratory previously examined the effects of a heat shock (23–37°C) upon the *C. albicans* transcriptome, and they also showed that several *HSP* genes were induced by their heat shock conditions (Enjalbert *et al*., 2003). Despite differences in the experimental conditions, the different *C. albicans* strains used (SC5314 versus THE1) and the different microarray platforms, there was reasonable overlap between this study and the current study with respect to the sets of *C. albicans* genes that were induced by heat shock (correlation coefficient = 0.52; *Supporting information*). Recently a third study compared the transcriptional responses of *C. albicans* SC5314 and *Candida dubliniensis* CD36 to a 30–42°C heat shock (Enjalbert *et al*., 2009). Our data were also consistent with this microarray study (correlation coefficient = 0.65), and there was also reasonable concordance between the two studies by Enjalbert *et al*. (2003; 2009) (correlation coefficient = 0.56).

A subset of 46 *C. albicans* genes was upregulated by heat shock in at least two of these three studies (*Supporting information*). These heat shock-inducible genes included classical heat shock genes (*HSP60*, *HSP70*, *HSP78*, *HSP90*, *HSP104*), as well as an array of chaperone-related functions (*KAR2*, *SBA1*, *SSA1*, *SSE1*, *STI1*, *YDJ1*), thereby reinforcing the view that protein folding is critical for heat shock adaptation in *C. albicans.* Stress-induced functions (*ASR1*, *CPR6*, *GRP2*, *RPN4*, *SBA1*, *SIS1*, *YDJ1*) and some transporters (*ALP1*, *HIP1*, *IFN1*, *ITR1*) were also included in this common set of heat shock-inducible genes.

Having defined the transcriptional response to heat shock in control cells, we then defined which *C. albicans* genes are dependent upon Hsf1 for their heat-shock regulation. To achieve this we took the set of heat shock-inducible genes in wild-type *C. albicans* cells. As described above, these genes were induced ≥ 2-fold by the 10 min 30–45°C heat shock in wild-type cells (THE1: *HSF1/HSF1*). Then to identify Hsf1-dependent genes, we compared the transcriptomes of doxycycline-treated and control *hsf1/tet*_*p*_*-HSF1* cells (CLM62-1) following an equivalent heat shock. Given that THE1 and CLM62-1 are isogenic, we reasoned that strain differences in their transcriptomes would be minimal, but that genes identified in *hsf1/tet*_*p*_*-HSF1* would also contain some that are affected by the doxycycline treatment used to achieve Hsf1 depletion. Therefore, control microarrays were performed to define which *C. albicans* genes were affected by doxycycline (*Supporting information*). The small number of genes whose expression was affected by doxycycline in THE1 cells was excluded from the list of Hsf1-dependent genes. To summarize, we defined Hsf1-dependent, heat shock-inducible genes in *C. albicans* as those that were: (i) upregulated ≥ 2-fold by heat shock in wild-type cells, but (ii) not significantly induced (< 2-fold regulation) by heat shock in *hsf1/tet*_*p*_*-HSF1* cells after Hsf1 depletion, and (iii) not significantly affected (< 2-fold regulation) by doxycycline treatment in wild-type cells (*Supporting information*).

**Fig. 6:**
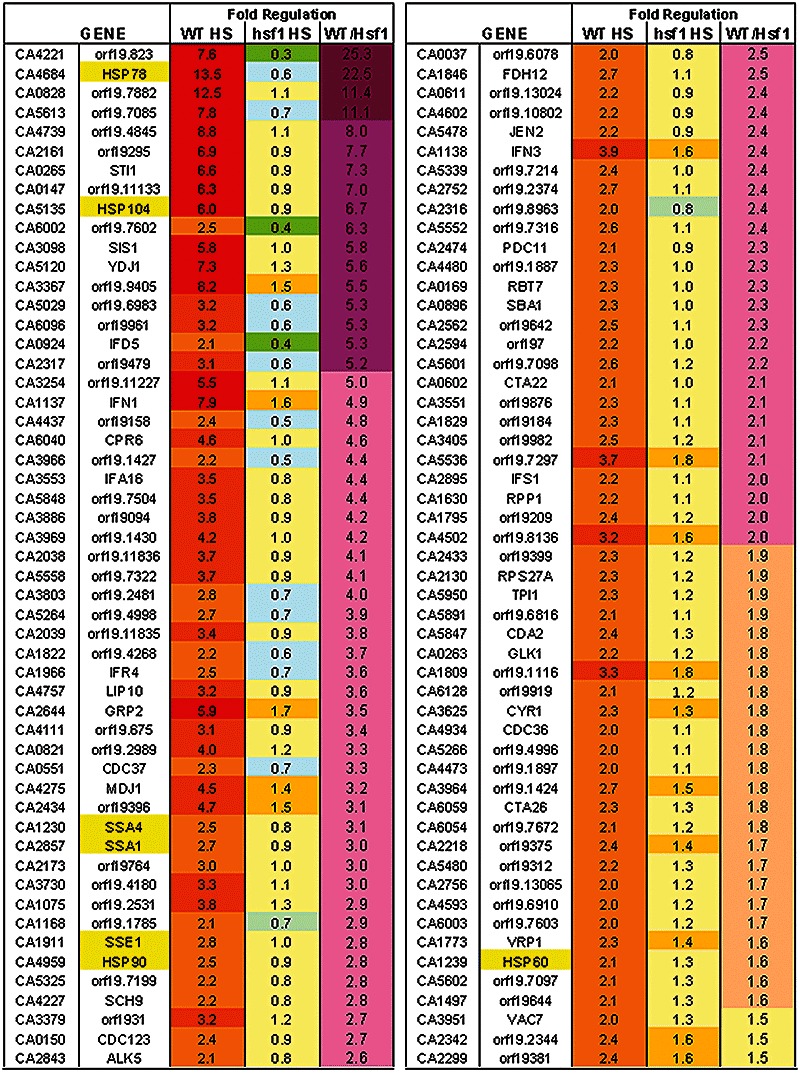
Hsf1-dependent heat shock-inducible genes in *C. albicans.* We list the subset of 116 *C. albicans* genes that displayed ≥ 2-fold upregulation in response to heat shock in wild-type cells, but not following Hsf1 depletion (i.e. the subset of upregulated genes highlighted in grey in Fig. 5A): WT HS, fold regulation in THE1 (*HSF1/HSF1*) cells in response to heat shock; hsf1 HS, fold regulation in doxycycline-treated *hsf1/tet*_*p*_*-HSF1* cells in response to heat shock; WT/Hsf1, ratio of fold regulation for a gene in THE1 cells compared with the fold regulation for that gene in doxycycline-treated *hsf1/tet*_*p*_*-HSF1* cells. *HSP* genes and *HSP70* family members are highlighted in yellow.

A large proportion of the heat shock-inducible genes in wild-type *C. albicans* cells were found to be dependent upon Hsf1 for their induction (116 of 136 genes; 86%) (Fig. 5A). This list of ‘Hsf1-dependent’ genes is presented in Fig. 6. Much of this Hsf1-dependent regulation might be indirect. We also note that the impact of Hsf1 upon the regulation of some of these genes was low (< 2-fold), but these genes were retained on the list because they conformed to our operational definition of Hsf1 dependence (i.e. significant heat shock induction in wild-type cells, but not in doxycycline-treated *hsf1/tet*_*p*_*-HSF1* cells). Nevertheless, Hsf1 had a large impact (≥ 2-fold) upon most of these genes (73%). Furthermore, genes encoding Hsp104, Hsp90 and members of the Hsp70 family were among those that displayed the strongest dependence upon Hsf1 for their heat shock induction (Fig. 6). This was entirely consistent with our Northern analyses (Fig. 3).

A significant proportion of the heat shock repressible genes were dependent upon Hsf1 for their downregulation (212 of 284 genes; 75%) (Fig. 5A). Not surprisingly therefore, the subsets of Hsf1-dependent genes displayed enrichment in similar cellular processes to the total subsets of heat shock-regulated genes, of which they are part (Fig. 5B). Again, much of this Hsf1-dependent regulation might be indirect. Nevertheless, these observations indicate that Hsf1 contributes significantly to the regulation of the heat shock transcriptome in *C. albicans*. The data also suggest that additional Hsf1-independent pathways contribute to the transcriptional response to heat shock. This is consistent with the situation in *S. cerevisiae*, where the general stress and cell wall integrity pathways also contribute to the regulation of the heat shock response (Gasch *et al*., 2000; Causton *et al*., 2001). However, it is already known that Msn2/4-like proteins do not contribute to the heat shock response in *C. albicans* (Nicholls *et al*., 2004). Therefore, other pathways must be involved in this pathogen.

A subset of 171 *C. albicans* genes was induced ≥ 2-fold by heat shock in doxycycline-treated *hsf1/tet*_*p*_*-HSF1* cells, but not in wild-type cells subjected to heat shock (Fig. 5). These genes were significantly enriched in functions involved in metabolism, ion transport and the processing of non-coding RNA (*Supporting information*). Similarly, 385 genes were downregulated ≥ 2-fold in response to heat shock in doxycycline-treated *hsf1/tet*_*p*_*-HSF1* cells, but were not downregulated in wild-type cells under equivalent conditions (Fig. 5). These genes displayed enrichment only in a single functional category: disaccharide metabolism (*Supporting information*). Most doxycycline-treated *hsf1/tet*_*p*_*-HSF1* cells were viable at the time of transcript profiling (Fig. 1). Therefore this transcriptional response appears to represent the impact of heat shock upon cells that are depleted of Hsf1 and hence are unable to mount the normal protective response that includes increased chaperone synthesis.

Our Northern analyses of the *HSP70*, *HSP90* and *HSP104* mRNAs also indicated that Hsf1 contributes to the basal expression of some *C. albicans* genes in the absence of heat shock (Fig. 3). Therefore, further microarray experiments were performed to ask which *C. albicans* genes are expressed in an Hsf1-dependent fashion under basal conditions. To achieve this we compared the transcriptomes of the conditional *hsf1/tet*_*p*_*-HSF1* mutant (CLM62-1) grown at 30°C in the presence or absence of doxycycline. Once again doxycycline-sensitive genes were excluded from the list of Hsf1-dependent genes (*Supporting information*). This revealed that Hsf1 contributes to the basal expression of 75 *C. albicans* genes even in the absence of heat shock. Significantly, this subset of Hsf1-dependent genes displayed significant enrichment in a single cellular process – protein folding [gene ontology (GO) term 4657; Fig. 5B]. This strongly supports the view that Hsf1 plays a key role in the modulation of protein folding-related functions in *C. albicans* even in the absence of stress.

### Hsf1 activates transcription through the HSE in *C. albicans*

In *S. cerevisiae*, Hsf1 stimulates the transcription of heat shock genes via multiple copies of the canonical HSE in their promoters ([nGAAn]_3_; Sorger and Pelham, 1988; Kirk and Piper, 1991; Santoro *et al*., 1998). In *C. albicans*, the promoters of some heat-inducible genes have been reported to contain sequences related to HSE (Swoboda *et al*., 1995; Sandini *et al*., 2002; Zenthon *et al*., 2006). However, the functionality of these HSE-like sequences has not yet been tested in *C. albicans.* Some regulatory elements are functionally conserved between *S. cerevisiae* and *C. albicans* in this yeast, such as the GCRE and YRE elements (Tripathi *et al*., 2002; Nicholls *et al*., 2004; Enjalbert *et al*., 2006). Therefore, it was attractive to predict that HSE-like sequences might mediate heat shock activation in *C. albicans*. It has also been reported that transcriptional activation by Hsf1 in *S. cerevisiae* can also be mediated by a second, non-standard HSE (nHSE: nGAAn[n]_5_nGAAn[n]_5_nGAAn; Yamamoto *et al*., 2005). Therefore, we tested the functionality of both the standard and non-standard HSE in *C. albicans*.

**Fig. 7:**
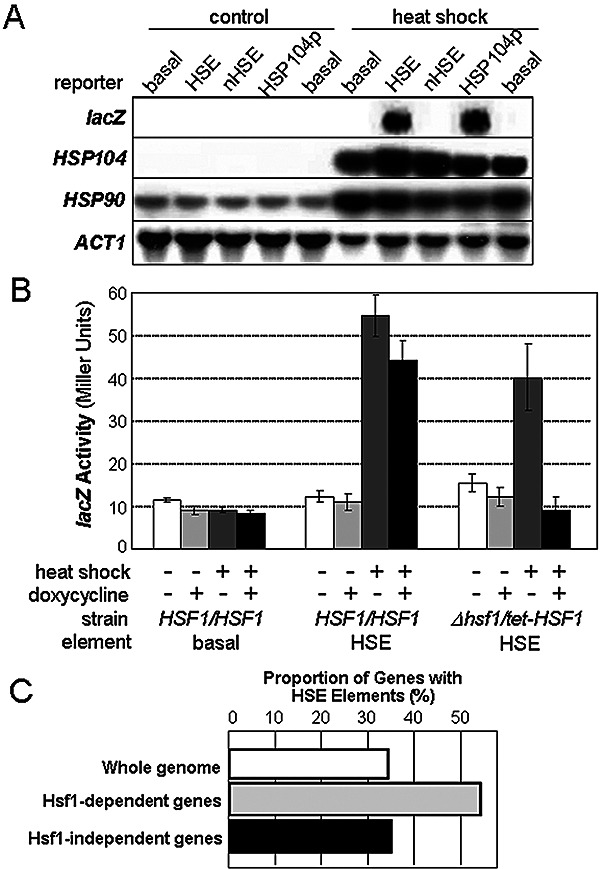
Hsf1 activates transcription in response to heat shock via the HSE in *C. albicans*. A. Northern analysis of the *lacZ*, *HSP90* and *HSP104* mRNAs in heat-shocked or control *C. albicans* cells containing the basal-*lacZ*, HSE-*lacZ*, nHSE-*lacZ* or *HSP104-lacZ* reporters (Table 1). B. β-Galactosidase activities displayed by heat-shocked or control *C. albicans* cells containing the HSE-*lacZ* reporter following growth with or without doxycycline: wild type, *HSF1*/*HSF1* (THE1); conditional *hsf1/tet*_*p*_*-HSF1* mutant (CLM62-1) (Table 1). C. Proportion of *C. albicans* genes that are induced by heat shock in an Hsf1-dependent and Hsf1-independent manner that contain HSEs in their promoters, compared with the *C. albicans* genome as a whole.

HSE-*lacZ* and nHSE-*lacZ* reporters were constructed by inserting HSE and nHSE oligonucleotides into the basal promoter region of our basal-*lacZ* reporter, which acted as our negative control. Also an *HSP104* promoter*-lacZ* reporter was made to act as a positive control. These reporters were transformed into *C. albicans* CAI4 (*HSF1/HSF1*) and their activity tested by Northern blotting under basal conditions and following heat shock (Fig. 7A). The blots were probed for the *ACT1* mRNA as a loading control. The blots were also probed for the wild-type *HSP104* and *HSP90* mRNAs, thereby confirming that the cells had been exposed to a bona fide heat shock. Our analyses of *lacZ* transcripts on these same blots revealed that, as expected, the *HSP104-lacZ* construct was induced by heat shock whereas the basal-*lacZ* reporter was not induced under these conditions. Significantly the *lacZ* reporter with the standard HSE was activated by heat shock, but the reporter containing the non-standard HSE was not. These data, which were confirmed by β-galactosidase assays (not shown), indicated that the standard HSE is functional in *C. albicans*, but suggested that the non-standard HSE is not. The non-standard HSE was not studied further.

To test whether this transcriptional activation via the HSE in *C. albicans* is dependent upon Hsf1, the HSE-*lacZ* reporter plasmids were transformed into the conditional *hsf1/tet*_*p*_*-HSF1* mutant (CLM62-1), and the control strains THE1 (*HSF1/HSF1*) and CLM61-1 (*hsf1/HSF1*). In medium lacking doxycycline, the HSE-*lacZ* reporter was induced in response to heat shock in all three strains, while the basal-*lacZ* reporter remained unresponsive (Fig. 7B). This reconfirmed the differential heat shock responsiveness of these reporters in these new strains. Following doxycycline addition, the HSE-*lacZ* reporter no longer responded to heat shock in *hsf1/tet*_*p*_*-HSF1* cells, but was still activated in the control strains. Therefore, the transcriptional activation mediated by the HSE in response to heat shock is dependent upon Hsf1.

If the HSE mediates transcriptional activation by Hsf1, one would expect this element to be enriched in the promoters of Hsf1-dependent genes. Therefore we examined the proportion of HSE-containing genes in the subsets of Hsf1-dependent and Hsf1-independent genes identified by transcript profiling. The promoters of *C. albicans* genes that were downregulated by heat shock displayed no significant HSE enrichment relative to the genome as a whole, whether this regulation was Hsf1-dependent or not. This suggested that any regulation of heat shock-repressed genes by Hsf1 is probably indirect. Similarly, the promoters of genes that were upregulated in response to heat shock in an Hsf1-independent manner showed no significant enrichment of the HSE (Fig. 7C). In contrast the HSE was over-represented in the promoters of genes that were induced by heat shock in an Hsf1-dependent manner (Fig. 7C). This reinforces the idea that these elements mediate transcriptional activation by Hsf1 in this yeast.

### *C. albicans* Hsf1 is activated in response to heat shock

**Fig. 8:**
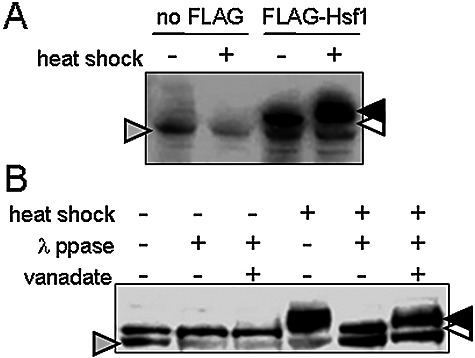
*C. albicans* Hsf1 is activated by phosphorylation in response to heat shock. A. Mid-exponential *C. albicans* cells were subjected to a 30–45°C heat shock or maintained at 30°C. Protein extracts were prepared and subjected to Western blotting with an anti-FLAG antibody: un-tagged cells (THE1; Table 1); cells containing FLAG-tagged Hsf1 (SN180). B. Control and heat-shocked extracts containing FLAG-tagged Hsf1 were treated with λ phosphatase in the presence or absence of the phosphatase inhibitor, sodium orthovanadate to confirm that the band shift was mediated by Hsf1 phosphorylation. Bands corresponding to inactive and activated (phosphorylated) Hsf1 are highlighted with white and black arrows, respectively, on the right. A background band observed in cells lacking the FLAG epitope is highlighted with the grey arrow on the left.

In *S. cerevisiae*, Hsf1 is activated in response to heat shock via hyperphosphorylation, which can be detected by mobility shifts on polyacrylamide gels (Sorger and Pelham, 1988). Our attempts to generate a specific anti-*C. albicans* Hsf1 antibody using recombinant Hsf1 or an Hsf1 peptide were unsuccessful. Therefore, we epitope-tagged Hsf1. The carboxy-terminal region of *S. cerevisiae* Hsf1 is important for its regulation (Hashikawa *et al*., 2006), and therefore we FLAG-tagged *C. albicans* Hsf1 at its amino-terminus, expressing the epitope-tagged construct from the *ACT1* promoter on pACT1-FLAG-HSF1 (*Experimental procedures*). Protein extracts were prepared from heat-shocked and control cells, and subjected to Western blotting with an anti-FLAG antibody. This revealed a specific FLAG-Hsf1 band in unstressed *C. albicans* cells, which shifted in mobility following heat shock (Fig. 8A). Treatment with λ phosphatase confirmed that this mobility shift was caused by phosphorylation (Fig. 8B). We conclude that *C. albicans* Hsf1 is activated by phosphorylation in response to heat shock.

### Do other stress conditions activate the HSF-HSE module in *C. albicans*?

**Fig. 9:**
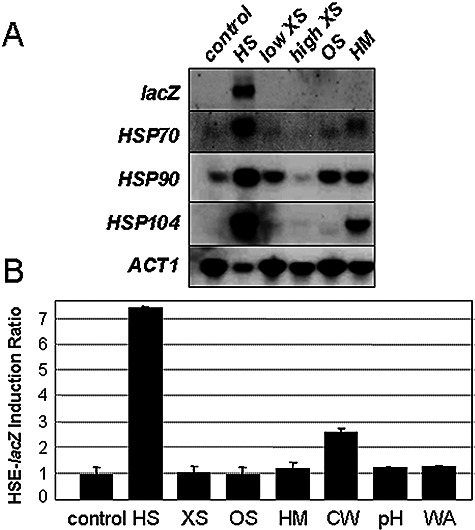
Activation of Hsf1 and HSE-*lacZ* by other stress conditions in *C. albicans. C. albicans* SN2 cells (CAI4 containing the HSE-*lacZ* reporter; Table 1) were grown in YPD at 30°C, and subjected to range of stress conditions for 30 min: control untreated cells; HS, 30–45°C heat shock; (high) XS, oxidative stress with 5 mM H_2_O_2_; low XS, oxidative stress with 0.4 mM H_2_O_2_; OS, osmotic stress with 1 M NaCl; HM, heavy metal stress with 0.5 mM CdSO_4_; CW, cell wall stress with 0.1% SDS; pH, pH stress at pH 3; and WA, weak acid stress with 20 mM acetic acid at pH 3. A. *lacZ* mRNA levels were examined by Northern analysis. B. HSE-*lacZ* induction ratio assayed by measuring β-galactosidase levels relative to the basal-*lacZ* control under equivalent conditions.

In *S. cerevisiae*, Hsf1 is activated in response to oxidative stress and glucose starvation, as well as by heat shock (Liu and Thiele, 1996; Hahn and Thiele, 2004). Furthermore, our previous work has suggested that additional, Hog1- and Cap1-independent oxidative stress signalling pathways remain to be discovered in *C. albicans* (Enjalbert *et al*., 2006). Therefore, to test whether the *C. albicans* Hsf1-HSE regulon is activated in response to other stresses, we examined HSE-*lacZ* expression levels following the exposure of wild-type *C. albicans* cells to a range of different stresses (Fig. 9). Measurements of both *lacZ* mRNA and β-galactosidase levels indicated that the HSE-*lacZ* reporter is unresponsive to osmotic, oxidative, heavy metal, weak acid and pH stresses. Slight induction of the HSE-*lacZ* reporter was observed for the cell wall stress (0.1% SDS), but the strongest induction was observed for heat shock. We conclude that the Hsf1-HSE regulon is essentially specific for heat shock.

We noted that the *HSP70*, *HSP90* and *HSP104* mRNAs were induced by stresses other than heat shock (Fig. 9). These genes may be controlled by other regulatory modules, in addition to the Hsf1-HSE regulon. Neither the oxidative stress induction of *CTA1* nor the osmotic stress induction of *PGA23* was blocked by Hsf1 depletion (Fig. 4). Nevertheless, we cannot exclude the possibility that Hsf1 might influence other stress modules as Hsf1 depletion reproducibly exerted unexpected effects upon *PGA23* regulation in response to heat shock and oxidative stress (Fig. 4).

### Modulation of the Hsf1-HSE regulon by growth temperature

**Fig. 10:**
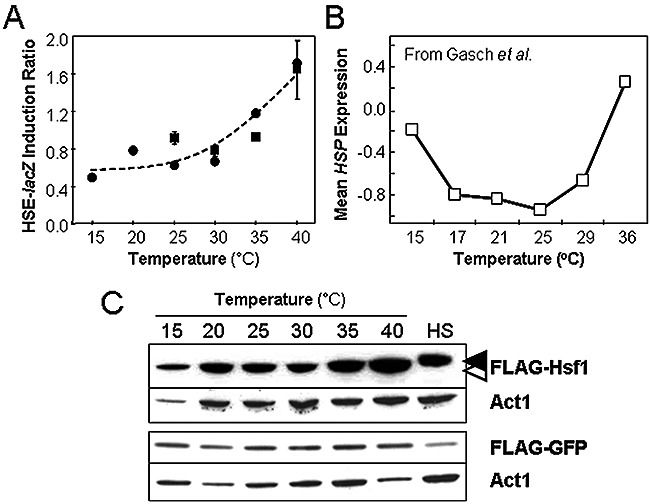
Activation of Hsf1 and HSE-*lacZ* in *C. albicans* at different growth temperatures. A. β-Galactosidase levels in *C. albicans* SN1 (HSE*-lacZ*) cells (Table 1) grown at different incubation temperatures in YPD (circles) or SD (squares). B. Impact of growth temperature on *HSP* transcript levels in *S. cerevisiae*. Supplementary data from Gasch *et al*. (2000) were used to calculate the median fold change in *S. cerevisiae HSP* transcript levels at each growth temperature examined in their study (units are fold changes measured on microarrays comprising PCR products on glass slides). C. FLAG-Hsf1 levels were examined by Western blotting of SN180 cells (pACT1-FLAG-HSF1; Table 1) grown in YPD at different temperatures. As a control, FLAG-GFP levels were examined in ML258 cells (pACT1-FLAG-GFP) grown under equivalent conditions. Act1 protein levels were examined in both cases as an internal control. Bands corresponding to inactive and activated (phosphorylated) Hsf1 are highlighted with white and black arrows respectively. Similar results were obtained in three independent experiments.

Most experiments that have examined yeast Hsf1-HSE regulons have focused on the effects of heat shock. However, sudden acute temperature upshifts, such as those imposed in the laboratory, probably occur rarely in the wild, especially for an obligate pathogen of warm-blooded animals. Therefore, we reasoned that in *C. albicans*, the Hsf1-HSE regulon might play a role in cellular adaptation to growth temperature, as well as in responses to temperature transitions. To test this we assayed HSE-*lacZ* expression levels during exponential growth at different growth temperatures (Fig. 10A). *C. albicans* cultures were grown at defined temperatures for over 2 days to mid-exponential phase (OD_600_ = 0.6–0.8), whereupon cells were harvested for analysis. We grew these *C. albicans* cultures in both rich (YPD) and minimal (SD) media to test whether medium composition affected the outcome of these experiments. HSE-*lacZ* expression levels increased with growth temperature, irrespective of the growth medium (Fig. 10). Although the different incubation temperatures affected the growth rate of these cultures, no correlation was observed between HSE-*lacZ* activity and doubling time (not shown), suggesting that the HSE-*lacZ* reporter does not respond to growth rate.

We then examined the impact of growth temperature on Hsf1 (Fig. 10). *C. albicans* SN180 (pACT1-FLAG-HSF1) and ML258 (pACT1-FLAG-GFP) were grown at different temperatures and FLAG-Hsf1 and FLAG-GFP levels measured by Western blotting. These blots were reprobed for the Act1 protein as an internal control. In contrast to FLAG-GFP levels, which remained relatively constant, FLAG-Hsf1 levels increased in response to growth temperature. *HSF1* transcript levels are not significantly affected by temperature (*Supporting information*; Nantel *et al*., 2002; Enjalbert and Whiteway, 2005), raising the possibility that Hsf1 levels might be regulated at a post-transcriptional level. FLAG-Hsf1 mobility was not altered in response to growth temperature (Fig. 10C), and control experiments confirmed that FLAG-Hsf1 was not phosphorylated in cells grown at 40°C (not shown). Taken together, these data suggest that while *C. albicans* Hsf1 phosphorylation is regulated by acute heat shock, Hsf1 levels may be modulated by growth temperature.

## Discussion

In this article we describe the identification of the *C. albicans* heat shock transcription factor (Hsf1) and demonstrate that Hsf1 regulates the expression of *C. albicans* genes through the canonical HSE ([nGAAn]_3_). Six observations support this view. First, Hsf1 was required for the transcriptional activation of an HSE reporter in heat-shocked *C. albicans* cells (Fig. 7B). Second, Hsf1 was activated in response to heat shock (Fig. 8). Third, HSE-containing oligonucleotides are able to form specific and temperature-responsive DNA–protein complexes with *C. albicans* extracts *in vitro* (Sandini *et al*., 2002). Although these authors presumed that these complexes were formed by the heat shock transcription factor, this was not confirmed experimentally. Fourth, the promoters of heat shock genes whose expression is dependent upon Hsf1 (Fig. 6) contain multiple HSEs (Swoboda *et al*., 1995; Sandini *et al*., 2002). Fifth, our genome-wide microarray analyses defined a subset of Hsf1-dependent genes in *C. albicans*, and revealed that the promoters of these genes display significant enrichment of the HSE (Fig. 7C). Sixth, 13 *S. cerevisiae* orthologues of Hsf1-dependent genes in *C. albicans* are bound directly by Hsf1 (as shown by chromatin immunoprecipitation in *S. cerevisiae*; Hahn *et al*., 2004). Of the 13 *C. albicans* genes that were induced by heat shock in an Hsf1-dependent fashion and whose *S. cerevisiae* orthologues are Hsf1 target genes, 11 encode HSPs, and the remaining two are induced by other stresses (*Supporting information*). Of these 11 *C. albicans HSP* genes, all contain HSEs in their promoters.

An additional, non-canonical HSE has been defined in *S. cerevisiae* (nGAAn[n]_5_nGAAn[n]_5_nGAAn; Yamomoto *et al*., 2005). However, in *C. albicans*, Hsf1 does not appear to activate transcription through this non-canonical HSE, at least in response to heat shock (Fig. 7).

Our microarray analyses identified those *C. albicans* genes that are induced in response to a 30–45°C heat shock. This gene set correlated closely with that defined in a previous study which examined a 23–37°C heat shock in *C. albicans* (Enjalbert *et al*., 2003), confirming that a bona fide heat shock response does exist in this pathogenic yeast. Taken together, these data sets show that in response to an acute temperature upshift, *C. albicans* induces classical heat shock genes and some other stress-regulated genes (Enjalbert *et al*., 2003, 2009; *Supporting information*). Furthermore, our microarray analyses extended these observations by defining Hsf1-dependent genes in *C. albicans* (Figs 5 and 6). Two main classes of Hsf1-dependent genes were identified. The first class was dependent upon Hsf1 for their induction in response to heat shock. This set of genes was strongly enriched in protein folding and refolding functions (Fig. 5). Not surprisingly therefore, Hsf1 depletion rendered *C. albicans* more sensitive to heat shock (Fig. 2). The second class was dependent upon Hsf1 for their basal expression in the absence of heat shock, a phenomenon that was confirmed by Northern blotting (Fig. 3). These genes were highly enriched for protein folding functions (Fig. 5). Significantly, they include chaperone-encoding*HSP70*, *HSP90* and *HSP104* genes, some of which are thought to be essential for viability in *C. albicans* (Swoboda *et al*., 1995). Clearly, Hsf1 plays critical roles in the modulation of protein folding under basal conditions as well as in response to heat stress. *C. albicans* cells that lack Hsf1 appear unable to express essential protein chaperones even in the absence of heat stress.

This probably explains why *HSF1* has been evolutionarily conserved in *C. albicans* and why Hsf1 is essential for the viability of this pathogen (Fig. 1). However, it does not explain why *C. albicans* has retained a heat shock response. *C. albicans* is viewed as an obligatory animal saprophyte (Do Carmo-Sousa, 1969; Odds, 1988), and as such would rarely be exposed to the acute temperature upshifts that are generally used to impose experimental heat shocks. Why then has *C. albicans* retained the ability to induce gene expression in response to heat shock during its co-evolution with warm-blooded mammalian hosts? We tested several possible explanations for this.

First, we reasoned that Hsf1-HSE activation might have been retained because it contributes to adaptive responses to other medically relevant stresses. For example, oxidative stress responses contribute to the pathogenicity of *C. albicans* and are activated during disease progression (Wysong *et al*., 1998; Hwang *et al*., 2002; Lorenz *et al*., 2004; Martchenko *et al*., 2004; Fradin *et al*., 2005; Enjalbert *et al*., 2006). In *S. cerevisiae* Hsf1 is activated by oxidative as well as thermal stresses (Liu and Thiele, 1996). Therefore, we tested whether the HSE-*lacZ* reporter and Hsf1 are activated by oxidative stress. We also examined weak acid and pH stresses because *C. albicans* cells might experience these stresses in the stomach and gastrointestinal tract. In addition we looked at cationic and osmotic stresses because *C. albicans* cells may be exposed to such stresses during infection of the oral cavity or kidney and during phagocytic attack by innate immune cells (Reeves *et al*., 2002). However, none of these stresses led to Hsf1-HSE activation (Fig. 9). Nevertheless, it is possible that this was due to the nature of the HSE sequence used in our *lacZ* reporter.

Weak HSE-*lacZ* induction was observed with a cell wall stress. This was interesting because Hsp90 is known to modulate the activity of the cell integrity pathway in *S. cerevisiae* (Duina *et al*., 1998), and cell wall stresses increase the resistance of *C. albicans* to antifungal therapy with echinocandins (Walker *et al*., 2008). Nevertheless, low levels of HSE-*lacZ* activation were observed for the cell wall stress in comparison with heat shock activation. Also, *HSP* genes are not significantly activated during the unfolded protein response in *C. albicans* (Wimalasena *et al*., 2008). We conclude that the Hsf1-HSE regulon is primarily involved in thermal stress responses. Therefore, the heat-shock responsiveness of Hsf1 does not appear to have been evolutionarily conserved because it contributes to other medically relevant stress responses.

We then sought an alternative explanation for this evolutionary conservation. We reasoned that the Hsf1-HSE regulon might play an important role in the thermal adaptation of *C. albicans* under normal growth conditions, in addition to its role during acute thermal transitions. Indeed, precedents for the involvement of heat shock transcription factor in thermal adaptation exist in the animal kingdom (Feder and Hofmann, 1999). For example, comparisons of ant and fly species that inhabit relatively cool and warm climes have revealed differences in the thresholds of activation for the heat shock transcription factor and in the expression levels for HSPs (Gehring and Wehner, 1995; Garbuz *et al*., 2003). Also, *S. cerevisiae HSP* gene expression is affected by growth temperature (Gasch *et al*., 2000). Therefore, we tested whether HSE-*lacZ* expression levels in *C. albicans* respond to growth temperature. This was the case, whether *C. albicans* cells were cultured in rich or minimal growth media. A strong correlation was observed between HSE-*lacZ* expression level and growth temperature, particularly when cells were grown at temperatures above 30°C (Fig. 10A), i.e. at temperatures that are physiologically relevant for this human pathogen. This behaviour is not dissimilar to the behaviour of *S. cerevisiae HSP* genes, although the expression of *S. cerevisiae HSP* genes is elevated at low temperatures (Fig. 10C; Gasch *et al*., 2000).

It was conceivable that the Hsf1-HSE activation was mediated by growth rate rather than temperature because *C. albicans* growth rate is influenced by incubation temperature. Furthermore, *HSP* gene expression levels have been correlated with growth rate in *S. cerevisiae* (Regenberg *et al*., 2006). These authors found a strong inverse correlation between growth rate and *HSP* gene expression in this model yeast. This is thought to be mediated through cAMP-PKA signalling, which downregulates the general stress response in *S. cerevisiae* (Gasch *et al*., 2000; Causton *et al*., 2001). Therefore, heat shock genes are generally expressed at higher levels in slower-growing *S. cerevisiae* cells. However, no such correlation was observed for *C. albicans* (*Supporting information*), possibly because the general stress response has diverged significantly in *C. albicans* (Enjalbert *et al*., 2006). We conclude that the Hsf1-HSE regulon contributes to long-term thermal adaptation in *C. albicans*, as well as to acute responses to sudden thermal transitions.

Several aspects of Hsf1-mediated regulation suggest a degree of transcriptional rewiring in *C. albicans* compared with *S. cerevisiae*. First, the Hsf1-HSE regulon appears to respond to heat shock in *C. albicans* (Fig. 9), whereas it responds to other stresses in *S. cerevisiae* (Liu and Thiele, 1996; Hahn and Thiele, 2004). Second, expression of the Hsf1-HSE regulon does not correlate inversely with doubling time in *C. albicans*, in contrast to the situation in *S. cerevisiae* (Regenberg *et al*., 2006; *Supporting information*). This is consistent with other well-documented examples of transcriptional rewiring in *C. albicans* that include Rfg1, a2, Gal4 and Msn2/4-like proteins (Kadosh and Johnson, 2001; Nicholls *et al*., 2004; Tsong *et al*., 2006; Martchenko *et al*., 2007), which in *C. albicans* are involved in cellular morphogenesis, cell type specification, metabolism and stress responses. However, the primary role of Hsf1, namely its central role in transcriptional regulation during thermal adaptation, has been conserved in *C. albicans.*

Taken together, our data suggest that the Hsf1-HSE regulon plays a crucial role in tuning chaperone levels to growth temperature by modulating the expression levels of genes such as *HSP70*, *HSP90* and *HSP104*. In the context of *C. albicans* infection, this routine homeostatic control of chaperone levels would facilitate adaptation of *C. albicans* to fluctuating growth temperatures, for example in febrile patients. We suggest that this scenario might be more relevant to this pathogen than the acute activation of repair mechanisms in response to sudden temperature shifts.

## Experimental procedures

### Strains and growth conditions

*Candida albicans* stains are listed in Table 1. Strains were grown in YPD medium, synthetic complete (SC) medium or SD minimal medium containing the appropriate supplements (Sherman, 1991). The expression of tetracycline-regulatable alleles was downregulated by addition of doxycycline to a final concentration of 20 μg ml^−1^. To heat stress *C. albicans*, cells were grown in YPD at 30°C for at least 6 h to mid-exponential phase, and then rapidly transferred to pre-warmed flasks at the desired temperature.

**Table 1:** *C. albicans* strains

Strain	Genotype	Source
SC5314	Clinical isolate	Gillum *et al*. (1984)
CAI4	*ura3::λ imm434/ura3::λ imm434*	Fonzi and Irwin (1993)
BWP17	*ura3::λ imm434/ura3::λ imm434, his1::hisG/his1::hisG, arg4::hisG/arg4::hisG*	Enloe *et al*. (2000)
THE1	*ade2::hisG/ade2::hisG, ura3::λ imm434/ura3::λ imm434,* *ENO1/eno1::ENO1-tetR-ScHAP4AD-3XHA-ADE2*	Nakayama *et al*. (2000)
CLM60-1	*ade2::hisG/ade2::hisG, ura3::λ imm434/ura3::λ imm434,* *ENO1/eno1::ENO1-tetR-ScHAP4AD-3XHA-ADE2, hsf1*::*hisG-URA3-hisG/HSF1*	This study
CLM61-1	*ade2::hisG/ade2::hisG, ura3::λ imm434/ura3::λ imm434,* *ENO1/eno1::ENO1-tetR-ScHAP4AD-3XHA-ADE2, hsf1*::*hisG/HSF1*	This study
CLM62-1	*ade2::hisG/ade2::hisG, ura3::λ imm434/ura3::λ imm434,* *ENO1/eno1::ENO1-tetR-ScHAP4AD-3XHA-ADE2, hsf1*::*hisG/URA3-tet*_*p*_*-HSF1*	This study
SN1	*ura3::λ imm434/ura3::λ imm434, pBasal-lacZ(URA3)*	This study
SN2	*ura3::λ imm434/ura3::λ imm434, pHSE-lacZ(URA3)*	This study
SN3	*ura3::λ imm434/ura3::λ imm434, pnHSE-lacZ(URA3)*	This study
SN4	*ura3::λ imm434/ura3::λ imm434, pPoly lacZ(URA3)*	This study
SN5	*ura3::λ imm434/ura3::λ imm434, pYER67p-lacZ(URA3)*	This study
SN6	*ura3::λ imm434/ura3::λ imm434, pHSP104p-lacZ(URA3)*	This study
SN7	*ade2::hisG/ade2::hisG, ura3::λ imm434/ura3::λ imm434,* *ENO1/eno1::ENO1-tetR-ScHAP4AD-33HA-ADE2, pPOLY-lacZ(URA3)*	This study
SN8	*ade2::hisG/ade2::hisG, ura3::λ imm434/ura3::λ imm434,* *ENO1/eno1::ENO1-tetR-ScHAP4AD-33HA-ADE2, pHSP104p-lacZ(URA3)*	This study
SN9	*ade2::hisG/ade2::hisG ura3::λ imm434/ura3::λ imm434,* *ENO1/eno1::ENO1-tetR-ScHAP4AD-33HA-ADE2, pBasal-lacZ(URA3)*	This study
SN10	*ade2::hisG/ade2::hisG ura3::λ imm434/ura3::λimm434,* *ENO1/eno1::ENO1-tetR-ScHAP4AD-33HA-ADE2, pHSE-lacZ(URA3)*	This study
SN11	*ade2*::*hisG/ade2*::*hisG, ura3*::*λ imm434/ura3*::*λimm434,ENO1/eno1*::*ENO1-tetR-ScHAP4AD-3*3*HA-ADE2hsf1*::*hisG/URA3-tet*_*p*_*-HSF1, pBasal-lacZ(NAT1)*	This study
SN12	*ade2*::*hisG/ade2*::*hisG, ura3*::*λimm434/ura3*::*λimm434,ENO1/eno1*::*ENO1-tetR-ScHAP4AD-3*3*HA-ADE2hsf1*::*hisG/URA3-tet*_*p*_*-HSF1, pHSE-lacZ(NAT1)*	This study
SN55	*ade2*::*hisG/ade2*::*hisG, ura3*::*λimm434/ura3*::*λimm434,ENO1/eno1*::*ENO1-tetR-ScHAP4AD-3*3*HA-ADE2, pnHSE-lacZ(URA3)*	This study
SN180	*ade2::hisG/ade2::hisG, ura3::λ imm434/ura3::λ imm434,* *ENO1/eno1::ENO1-tetR-ScHAP4AD-3XHA-ADE2 HSF1/HSF1,* *pACT1-FLAG-HSF1*	This study
ML258	*ura3::λ imm434/ura3::λ imm434, his1::hisG/his1::hisG,* *arg4::hisG/arg4::hisG, HSF1/HSF1, pACT1-FLAG-*GFP	This study

### Strain construction

To generate doxycycline-conditional *C. albicans HSF1* mutants, the first *HSF1* allele was deleted using the mini Ura-blaster cassette as described previously (Wilson *et al*., 2000). Briefly, the *hsf1*::*hisG-URA3-hisG* disruption cassette was created by PCR amplification with the primers Hsf1-3DR and Hsf1-5DR (*Supporting information*). Following transformation into *C. albicans* THE1, this resulted in the deletion of codons 12–746 of the 762 codon *HSF1* open reading frame. This generated heterozygous Δ*hsf1/HSF1* mutants (e.g. CLM60-1) (Table 1). The *Ura3-*minus segregant CLM61-1 was then selected by growth on media containing 5-fluoroorotic acid (Wilson *et al*., 2000). Then the doxycycline-regulatable *tet*_*p*_ promoter was inserted upstream of the remaining *HSF1* open reading frame. To achieve this, a *URA3-tet*_*p*_*-HSF1* cassette was created by PCR amplification using primers described in *Supporting information* (Nakayama *et al*., 2000) and transformed into CLM61-1 to create the conditional Δ*hsf1/tet*_*p*_*-HSF1* mutant, CLM62-1 (Table 1). The genotype of each strain was confirmed by PCR diagnosis and by Southern analysis (not shown).

Hsf1 was amino-terminally tagged with the FLAG epitope by replacing the GFP open reading frame in pACT1-FLAG-GFP with the *HSF1* coding region to create pACT1-FLAG-HSF1. pACT1-FLAG-GFP was made by inserting a double-stranded oligonucleotide encoding three copies of the FLAG epitope (*Supporting information*) into the HindIII site in pACT1-GFP, creating a new BamHI site (Barelle *et al*., 2004). pACT1-FLAG-GFP and pACT1-FLAG-HSF1 were then transformed into *C. albicans* to create ML258 and SN180 respectively (Table 1). FLAG-GFP and FLAG-Hsf1 are expressed from the *C. albicans ACT1* promoter in these cells.

Reporter constructs were based on the *Streptococcus thermophilus lacZ* gene (Uhl and Johnson, 2001). To create *HSE-lacZ* reporters, synthetic oligonucleotides containing standard or non-standard HSE sequences (HSE-T, HSE-B, nHSE-T and nHSE-B; *Supporting information*) were cloned into the SalI site upstream of the basal *ADH1* promoter in the CIp10-based *URA3* plasmid, pLacBasal (Nicholls *et al*., 2004). The *basal-lacZ*, *HSE-lacZ* and *nHSE-lacZ* constructs were transformed into *C. albicans* CAI4, THE1 and CLM61-1 cells using the *URA3* marker (Murad *et al*., 2000). The *NAT1* marker was cloned into these *lacZ* plasmids to select nourseothricin-resistant transformants in CLM62-1 cells (Reuss *et al*., 2004). Correct integration was confirmed by PCR diagnosis with the oligonucleotides RPS1-GEN and LacZ-F (*Supporting information*).

### Southern analysis and mRNA quantification

Published methods were used for RNA and DNA preparation, Southern blotting and Northern analyses (Wicksteed *et al*., 1994; Brown *et al*., 2001). A non-radioactive kit was used for detection of the probe (ECL Direct™ Nucleic Acid Labelling and Detection Systems, Amersham, UK). *ACT1*, *HSP90* and *HSP104* probes were specific for their corresponding mRNAs on Northern blots. However, Southern analysis of gene-specific PCR products showed that the *HSP70* probe cross-reacted with transcripts from several *HSP70* family members: *HSP70*, *SSA2*, *SSB1 KAR2* and *SSC1* (not shown).

In some experiments real-time RT-PCR was used to measure the levels of the *ACT1*, *EFB1*, *HSP90*, *CTA1* and *PGA23* transcripts using the primers listed in *Supporting information*. RNA samples (2 μg) were treated in a 20 μl reaction mix with 1.5 μl of DNase I, 1.5 μl of RNase OUT, 2 μl of DNase I buffer (Invitrogen) at room temperature for 15 min. cDNA was prepared using Superscript II (Invitrogen) as per the manufacturer's protocol. Real-time RT-PCR SYBR green (Roche) assays were carried out as per the manufacturer's instructions using The LightCycler® 480 Real-Time PCR System (Roche).

### Transcript profiling

Transcript profiling of *C. albicans* strains THE1 (*HSF1/HSF1*) and CLM62-1 (*hsf1/tet*_*p*_*-HSF1*) was performed on exponential cells growing in YPD medium. Where appropriate, doxycycline was added to cultures for at least 6 h before heat shock. Heat stress was imposed by rapidly shifting cells from 30°C to 45°C and incubating for 10 min. Cells were frozen rapidly in liquid nitrogen, sheared mechanically using a microdismembrator (Braun, Melsungen, Germany) and RNA prepared by extraction with Trizol Reagent (GibcoBRL, Grand Island, NY) as described previously (Hauser *et al*., 1998). Cy3- and Cy5-labelled cDNAs were prepared from total RNA, and the probes were hybridized with whole-genome oligo-based microarrays containing 6000 *C. albicans* genes (Eurogentec, Seraing, Belgium) using published methods (Enjalbert *et al*., 2006). Slides were scanned using a proScanArray HT (PerkinElmer Life Sciences, Beaconsfield, UK) and quantified using ScanArray Express (version 4). Data were normalized and analysed using GeneSpring (Silicon Genetics, Redwood City, CA), and statistical analyses were performed using SAM (Significance Analysis of Microarrays; Tusher *et al*., 2001). Data from at least three independent biological replicates were used for each analysis, and the SAM False Discovery Rate was set at 10% (Enjalbert *et al*., 2006). The complete data set is available in a MIAME-compliant format at ArrayExpress (Accession No. E-MEXP-2044 and E-MEXP-1369).

Expression ratios were calculated by comparing stressed cells with the corresponding unstressed control, or by comparing doxycycline-treated cells with the corresponding untreated control. For example, heat shock-regulated genes were defined as those whose transcript levels were affected at least twofold by heat shock in wild-type (THE1) cells (compared with unstressed THE1 cells). Of these heat shock-regulated genes, those whose heat shock regulation was dependent upon Hsf1 were then defined as those whose transcript levels were no longer regulated at least twofold by heat shock in doxycycline-treated *tet*_*p*_*-HSF1* cells (compared with unstressed doxycycline-treated *tet*_*p*_*-HSF1* cells). Finally, genes whose basal expression levels were dependent upon Hsf1 were defined as those whose transcript levels were: (i) affected at least twofold by the addition of doxycycline to *tet*_*p*_*-HSF1* (CLM62-1) cells in the absence of heat stress (compared with untreated CLM62-1 cells in the absence of stress), and (ii) not affected in wild-type (THE1) cells under equivalent experimental conditions.

Functional categories for *C. albicans* genes were assigned using GO resources at CGD (http://www.candidagenome.org/cgi-bin/GO/goTermFinder), and on the basis of MIPS functional assignments for *S. cerevisiae* homologues (http://mips.gsf.de/proj/yeast/CYGD/db/index.html), as described previously (Yin *et al*., 2004). Promoter analyses were performed using GeneSpring.

### Reporter assays

*lacZ* expression levels were assayed in quadruplicate on independent transformants as described previously (Rupp, 2002). Briefly, *C. albicans* cells were grown for at least 6 h to exponential phase. Half of each culture was subjected to a stress for 30 min and the other half acted as the untreated control. Cells were harvested and re-suspended in 1 ml of Z buffer (60 mM Na_2_HPO_4_, 40 mM NaH_2_PO_4_, 10 mM KCl, 1 mM MgSO_4_, 50 mM β-mercaptoethanol), and then 50 μl of chloroform and 20 μl of 1% SDS were added. Samples were equilibrated at 37°C for 10 min, and then reactions started by addition of 200 μl of pre-warmed OPNG (4 mg ml^−1^). Samples were incubated until a yellow colour developed whereupon the reaction was stopped by addition of 0.4 ml of 1 M Na_2_CO_3_. β-Galactosidase activities were measured in Miller units.

### Protein extraction and Western blots

Total soluble protein was extracted and subjected to Western blotting using published protocols (Smith *et al*., 2004). Briefly, cells were re-suspended in 250 μl of lysis buffer (0.1 M Tris-HCl, pH 8, 10% glycerol, 1 mM DTT, pepstatin A, Protease Inhibitor Cocktail) and sheared with glass beads in a Mini-bead beater (6 × 30 s with 1 min intervals on ice). Lysates were centrifuged at 13000 r.p.m. for 10 min at 4°C. Protein extracts (15 μg) were subjected to SDS-PAGE electrophoresis, blotted for 2 h at 30 V, and membranes blocked for at least 1 h at room temperature using 5% BSA. Membranes were probed overnight with a rabbit anti-FLAG HRP-conjugated primary antibody (diluted 1/200 000: Sigma), washed and signals detected with an HRP Western blotting kit (Amersham, UK).
