# Supplementary material for: Role of the heat shock transcription factor, Hsf1, in a major fungal pathogen that is obligately associated with warm-blooded animals
Source: Mol Microbiol. 2009 Oct 8;74(4):844–61. doi: 10.1111/j.1365-2958.2009.06883.x (PMC3675641; doi:10.1111/j.1365-2958.2009.06883.x)
Supplement: Supplementary file 1 [file mmi0074-0844-SD1.pdf]

Summary of Supporting information

	**Title**	**Description**
1	All Data	Complete transcript profiling dataset
2	Heat shock overlap - 1	Overlapping subsets of heat shock genes from three microarray studies
3	Heat shock overlap - 2	Fold regulation of heat shock induced genes from three microarray studies
4	Hsf1 regulated genes	*C. albicans* genes that were up-regulated in response heat shock in an Hsf1-dependent manner
5	Hsf1 basal genes	*C. albicans* genes that displayed Hsf1-dependence under basal conditions
6	Up only in hsf1	*C. albicans* genes that were up-regulated by heat shock only following Hsf1 depletion
7	Down only in hsf1	*C. albicans* genes that were down-regulated by heat shock only following Hsf1 depletion
8	Dox genes	Genes that were sensitive to doxycycline in wild type *C. albicans* cells
9	ChIP comparison	Comparison of heat shock inducible, Hsf1-dependent genes in *C. albicans* with Hsf1-dependent genes in *S. cerevisiae* identified by chromatin immunoprecipitation
10	*S. cerevisiae* data	Impact of Temperature and Growth Rate upon *HSP* gene expression in *S. cerevisiae*
11	Oligos	Oligonucleotides used in this study

1) Complete Microarray Dataset

The complete dataset is available in a MIAME-compliant format at ArrayExpress at

http://www.ebi.ac.uk/microarray-as/ae/

Accession numbers:

E-MEXP-2044

E-MEXP-1369

2) Overlapping subsets of heat shock genes from three microarray studies

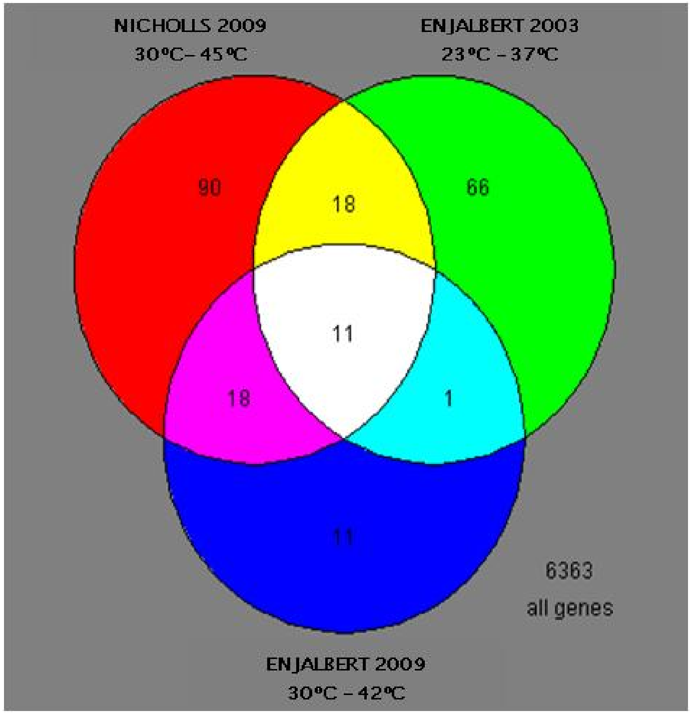

CA3415	FRE30.53	orf19.6140	Strong similarity to ferric reductase, internal fra	CA4220	IPF8762	orf19.822	unknown function
CA1734	IPF4988	orf19.2529	unknown function				
CA0183	CAP1	orf19.9191	transcriptional acivator Cap1 [Candida albican	CA5613	IPF525	orf19.7085	unknown function
CA6159	IPF1837	orf19.5877	unknown function	CA1230	SSA4	orf19.4980	cahsp70 mRNA for heat shock
CA2615	CTA241.E>	orf19.5700	transcriptional activator, exon 1	CA4111	IPF3964	orf19.675	unknown function
CA3851	CTA21	orf19.6112	transcriptional activation	CA0828	IPF17186	orf19.7882	unknown function

CA3796	SGT2	orf19.5823	small glutamine-rich tetratricopeptide repeat cc	CA1137	IFN1	orf19.1979	glycerophosphoinositol transporter (by homolo
CA0050	CTA25	orf19.362	transcriptional activation	CA2342	IPF13836	orf19.2344	probable heat shock protein (by homology)
CA4804	ARO8	orf19.2098	aromatic amino acid aminotransferase I (by ho	CA0896	SBA1	orf19.5749	Hsp90 (Ninety) Associated Co-chaperone (by I
CA2059	CDC4	orf19.2559	CANAL CELL DIVISION CONTROL PROTEIN	CA3098	SIS1	orf19.3861	heat shock protein (by homology)
CA4265	GAP6	orf19.6659	General amino acid permease (by homology)	CA0602	CTA22	orf19.3074	Protein with putative transcription activation do
				CA6002	IPF661	orf19.7602	unknown function
**CA0495**	IPF20056	orf19.2659	unknown function	CA2038	IPF17510	orf19.1183	unknown function
CA1943	IPF11759						
CA5547	IPF5981	orf19.7310	similar to Saccharomyces cerevisiae Gin3p (by	CA3367	IPF4667	orf19.9405	unknown Function
CA2075	IFE2	orf19.5288	Unknown function	CA2644	GRP2	orf19.4309	Reductase (by homology)
CA5526	IPF2857	orf19.7284	unknown function	CA3254	IPF4728	orf19.1122	unknown Function
CA4779	IPF3351	orf19.4012	unknown function	CA5891	IPF2400	orf19.6816	putative aldehyde reductase (by homology)
CA0005	IPF14994	orf19.2414	unknown function	CA0558	GPX2	orf19.85	glutathione peroxidase (by homology)
CA0782	IPF16253	orf19.1141	unknown function	CA4437	IPF13867	orf19.5158	unknown function
CA2167	IPF4553	orf19.909	unknown function	CA5339	IPF885	orf19.7214	glucan 1,3-beta-glucosidase (by homology)
CA0386	IPF4065	orf19.1862	unknown function	CA0037	IPF17652.3	orf19.6078	reverse transcriptase, 3-prime end (by homolo
CA3945	CHA12	orf19.9548	L-serine/L-threonine deaminase (by homology)	CA0263	GLK1	orf19.1408	aldohexose specific glucokinase (by homology
CA2392	IPF15672	orf19.1013	unknown function	CA0394	IPF12758.5	orf19.7966	unknown function, 5-prime end
CA0943	YHB1	orf19.3707	flavohemoglobin (by homology)	CA1075	IPF4991	orf19.2531	putative membrane prc putative membrane pro
CA5864	IPF364	orf19.7522	transaminase type I (by homology)	CA2712	SPE2	orf19.8199	by homology to S. cerevisiae adenosylmethion
CA2913	IPF11205	orf19.4513	unknown function	CA3886	IPF12963	orf19.5094	ubiquitin-mediated protein degradation (by hon
CA4184	SNZ1	orf19.2947	stationary phase protein by homology	CA6059	CTA26	orf19.7680	transcriptional activation
CA2738	STF2		ATP synthase regulatory factor (by homology)	CA1497	IPF9683	orf19.3644	unknown function
CA6080	IPF21	orf19.5980	unknown function	CA5817	IPF2511	orf19.7468	unknown function
CA4158	DLD3	orf19.5805	D-lactate ferrycytochrome C oxidoreductase (b	CA2299	IPF8222	orf19.5381	unknown function
CA0617	MET2	orf19.2618	Homoserine O-acetyltransferase	HSP104	Heat shock	orf19.13747	
CA5179	MAK3	orf19.4617	N-acetyltransferase (by homology)				
CA1429	IPF15123	orf19.1317	unknown function	CA4684	HSP78.5F	orf19.882	heat shock protein of c heat shock protein 78 [
CA2697	IPF9167	orf19.2737	unknown function	CA6040	CPR6	orf19.7654	cyclophylin (by homology)
CA0875	ERG251	orf19.1210	C-4 sterol methyl oxidase (by homology)	CA4683	HSP78.3F	orf19.884	heat shock protein of clpb family of ATP-deper
CA5277	CTA29.EX(	orf19.7127	Protein with putative transcription activation dc	CA0821	IPF16795	orf19.2989	glycerate/formate-dehy YNL274C homologue [

CA2473	CBF1	orf19.2876	putative centromere binding factor 1	CA0265	STI1	orf19.1070:	stress-induced protein (by homology)	
CA5968	IPF946	orf19.7561	unknown function	CA5120	YDJ1	orf19.6408	Mitochondrial and ER import protein (by homol	
CA2834	IPF12093	orf19.4786	unknown function	CA2857	SSA1	orf19.1065	Heat shock protein of HSP70 family	
CA3105	IPF12083	orf19.6553	unknown function	CA1239	HSP60	orf19.717	Heat Shock Protein 60 (HSP60)	
CA1751	ARO3.EXC	orf19.1517	3-deoxy-D-arabinoheptulosonate-7-phosphate	CA4502	IPF10391	orf19.8136	Similar to dnaJ proteins	
CA3099	LST8	orf19.3862	required for transport of permeases from	CA4959	HSP90	orf19.6515	heat shock protein	heat shock protein 90 [
CA2649	IPF9057	orf19.1189	unknown function	CA0915	KAR2	orf19.9564	dnaK-type molecular chaperone (by homology)	
CA3771	SOU1	orf19.2896	Sorbitol utilization protein Sou1p [Candida albi	CA4162	MET1	orf19.5811	siroheme synthase (by homology)	
CA3255	IPF4729	orf19.3743	unknown Function	CA2854	RPN4	orf19.1069	26S proteasome subunit (by homology)	
CA1834	PFK1	orf19.3967	6-phosphofructokinase, alpha subunit	CA0863	ITR2	orf19.3526	Myo-inositol transporter (by homology)	
CA3141	IPF9211.3F	orf19.3712	unknown function, 3-prime end	CA3708	HIP1	orf19.4940	Histidine permease (by homology)	
CA1099	IPF12312	orf19.1253(	unknown function	CA1911	SSE1	orf19.2435	heat shock protein of HSP70 family (by homolo	
CA4024	IPF7182	orf19.3439	unknown function	CA3334	ALP1	orf19.2337	amino-acid permease (by homology)	
CA0356	IPF2532	orf19.1395:	unknown function	CA2474	PDC11	orf19.2877	Pyruvate decarboxylase (by homology)	
CA0458	IPF15601	orf19.2433	unknown function					
CA2659	NDH2	orf19.5713	NADH dehydrogenase (by homology)					
CA2679	IFU5	orf19.2568	Unknown function					
CA0748	TFS1	orf19.1974	cdc25-dependent nutrient- and ammonia-response cell-cycle regulator (by homology)					
CA5572	BGL22	orf19.7339	endo-beta-1,3-glucanase (by homology)					
CA5874	CTA23	orf19.7544	transcriptional activation					
CA0916	LYS2	orf19.2970	L-aminoadipate-semialdehyde dehydrogenase, large subunit					
CA4085	IPF5015	orf19.6641	unknown function					
CA1367	IPF16755	orf19.4966	unknown function					
CA4164	IPF9302	orf19.1323!	unknown function					
CA1880	IPF13316	orf19.2269	unknown function					
CA3727	HIS5.3F	orf19.4177	Histidinol-phosphate aminotransferase, 3-prime end (by homology)					
CA6166	CTP1	orf19.1329:	Citrate transport protein (by homology)					
CA2846	PAD1	orf19.5731	phenylacrylic acid decarboxylase (by homology)					
CA0854	IPF8854	orf19.4900	similar to Saccharomyces cerevisiae Mnn1p alpha-1,3-mannosyltransferase (by homology)					
CA4039	SKS1	orf19.3669	serine/threonine kinase by homology					
CA4183	SNO1	orf19.1046!	hisH-like protein (by homology)					

CA5007	IPF12579	orf19.5239	putative phospholipase A2 (by homology)	
CA5658	IPF1242	orf19.7360	unknown function	
CA4266	IPF2283	orf19.6660	unknown function	
CA2131	IDP1	orf19.5211	isocitrate dehydrogenase, cytosolic (by homology)	
CA3528	GLY1	orf19.8601	L-threonine aldolase	
CA2190	AOX1	orf19.4774	alternative oxidase (by homology)	
CA2439	IPF7514	orf19.2197	unknown function	
CA3329	IPF12162	orf19.2670	Unknown function	
CA5926	PRO2	orf19.6779	Proline biosynthetic enzyme (by homology)	
CA3119	IPF5360.3		unknown function, 3-prime end	
CA1882	IPF19934	orf19.9755	unknown function	
CA4739	IPF4498	orf19.4845	unknown function	
CA4221	IPF8760	orf19.823	unknown function	
CA2161	IPF11876	orf19.5295	unknown function	
CA0147	IPF9690	orf19.1113[illegible]	unknown function	
CA2434	IPF9484	orf19.3396	unknown function	conserved hypothetical protein [Candida albicans] Length = 151
CA4275	MDJ1	orf19.6672	Heat shock protein - chaperone (by homology)	
CA3969	IPF9379	orf19.1430	unknown function	
CA1138	IFN3	orf19.1978	glycerophosphoinositol transporter (by homology)	
CA2968	BMR1	orf19.5604	benomyl/methothrexate resistance protein (by homology)	
CA5558	IPF1969	orf19.7322	unknown function	
CA5536	IPF2837	orf19.7297	putative cystathionine gamma-synthase (by homology)	
CA3553	IFA16.5	orf19.3878	unknown function, 5-prime end	
CA5848	IPF407	orf19.7504	unknown function	
CA2039	IPF12947	orf19.1183[illegible]	unknown function	
CA1809	IPF6325	orf19.1116	unknown function	
CA3730	IPF11900	orf19.4180	unknown function	
CA5341	HSP10.3		10 kDa mitochondrial heat shock chaperonin, 3-prime end (by homology)	
CA5029	IPF2997	orf19.6983	unknown function	
CA6096	IPF56	orf19.5961	similar to Saccharomyces cerevisiae Nas6p subunit of 26S proteasome (by homology)	

CA3379	IPF8350	orf19.331	putative methyltransferase (by homology)
CA4757	LIP10	orf19.4822	Secretory lipase
CA1631	IPF19582	orf19.1028	unknown function
CA2317	IPF13586	orf19.5479	unknown function
CA2173	IPF18527	orf19.3764	unknown function
CA1786	IPF7629	orf19.1168	unknown function
CA4463	PIS1	orf19.6860	CDP diacylglycerol--inositol 3-phosphatidyltransferase (by homology)
CA3803	IPF12407	orf19.2481	unknown function
CA2752	IPF6238	orf19.2374	GAG protein of retrotransposon pCal
CA5264	IPF1798	orf19.4998	unknown function
CA3964	IPF9370	orf19.1424	unknown function
CA1846	FDH12	orf19.638	Formate dehydrogenase (by homology)
CA4894	IPF1617	orf19.6586	unknown function
CA5227	IPF3087	orf19.7046	unknown function
CA3685	IPF19540	orf19.723	unknown function
CA5601	IPF556	orf19.7098	transcriptional regulator (by homology)
CA5552	IPF5971	orf19.7316	unknown function
CA3405	IPF8644	orf19.3982	maltase (by homology)
CA3545	BPT1.5F	orf19.6383	membrane transporter of the ATP-binding cassette (ABC) superfamily, 5-prime end (by homology)
CA1966	IFR4	orf19.2394	unknown function
CA2562	IPF19953	orf19.5642	unknown function
CA4581	IPF2232	orf19.6899	unknown function
CA2218	IPF18508	orf19.5375	unknown function
CA1795	IPF11261	orf19.5209	unknown function
CA0150	CDC123	orf19.1023[illegible]	similar to Saccharomyces cerevisiae Cdc123p cell cycle regulator (by homology)
CA5847	CDA2	orf19.7503	chitin deacetylase (by homology)
CA4348	IPF3638	orf19.6723	unknown function
CA5950	TPI1	orf19.6745	Triose phosphate isom triose phosphate isomerase [Candida albicans] Length = 248
CA1829	IPF17177.[illegible]	orf19.5184	similar to Saccharomyc regulator of chromosome condensation [Candida albicans] Length = 492
CA4480	IPF6464	orf19.1887	putative triacylglycerol lipase (by homology)
CA1773	VRP1	orf19.2190	verprolin (by homology)

CA1908	APL3	orf19.2786	AP-2 complex subunit, alpha-adaptin (by homology)	
CA2433	IPF12959	orf19.3399	unknown function	
CA3551	IPF13229	orf19.3876	unknown function	
CA0551	CDC37	orf19.5531	Cell division control prc	Cdc37 [Candida albicans] Length = 508
CA2361	IPF8950	orf19.1236	unknown function	
CA3625	CYR1.3F	orf19.1261	adenylate cyclase, 3-pr	adenylyl cyclase [Candida albicans] Length = 1690
CA2130	RPS27A		ribosomal protein S27.e (by homology)	
CA0169	RBT7	orf19.1019	repressed by TUP1	
CA2594	IPF12824	orf19.57	unknown function	
CA2895	IFS1	orf19.2461	Unknown function	
CA0611	IPF17542	orf19.1302	unknown function	
CA4602	IPF6231	orf19.1080	unknown function	
CA4227	SCH9	orf19.829	strong similarity to S.pombe sck1	protein kinase (by homology)
CA5480	IPF8210	orf19.5312	unknown function	
CA1822	IPF9525	orf19.4268	unknown function	
CA5478	JEN2	orf19.1276	carboxylic acid transporter protein (by homology)	
CA1630	RPP1	orf19.1029	required for processing of tRNA and 35S rRNA (by homology)	
CA5325	IPF2138	orf19.7199	unknown function	
CA3966	IPF9376	orf19.1427	unknown function	
CA5846	IPF409	orf19.7502	unknown function	
CA6128	IPF152	orf19.5919	unknown function	
CA6054	IPF4952	orf19.7672	unknown function	
CA2843	ALK5.5F	orf19.5728	n-alkane-inducible cytochrome P-450, 5-prime end	
CA0924	IFD5	orf19.1048	Putative aryl-alcohol dehydrogenase (by homology)	
CA0498	CTA211.3F	orf19.2661	transcriptional activator, 3-prime end (by homology)	
CA1168	IPF13017	orf19.1785	unknown function	
CA2874	IPF4999	orf19.5843	unknown function	
CA5602	IPF554	orf19.7097	RNA binding protein (by homology)	
CA2316	IPF14542	orf19.8963	unknown function	
CA2338	NIT3	orf19.2351	nitrilase (by homology)	
CA0796	ALR1	orf19.9175	divalent cation transpo	unnamed protein product [Candida albicans] Length = 922

CA4593	IPF2214	orf19.6910	unknown function	
CA4473	IPF6447	orf19.1897	unknown function	
CA1399	IPF8069	orf19.3698	unknown function	
CA6003	IPF660	orf19.7603	unknown function	
CA5266	IPF1787.3P	orf19.4996	unknown function, 3-prime end	
CA3951	VAC7.3	orf19.1409	Vacuolar protein, 3-prime end (by homology)	
CA4934	CDC36	orf19.6075	transcription factor (by homology)	
CA2756	IPF18418	orf19.1306[illegible]	unknown function	hypothetical protein [Candida albicans] Length = 302

3) Fold regulation of heat shock induced genes from three microarray studies

1. Enjalbert *et al.* (2003). Molec. Biol. Cell. 14, 1460-1467.
2. Enjalbert et al. (2009) Under revision.
3. This study

Heat shock (°C)		**23-37**	**30-42**	**30-45**
HSP12	**CA0627**	**1.7**	**1.9**	**9.8**
HSP30	**CA1507**	**0.8**	**1.0**	**1.2**
HSP70	**CA1230**	**5.1**	**11.4**	**2.5**
HSP78	**CA4684**	**1.9**	**6.9**	**13.5**
HSP90	**CA4959**	**1.4**	**2.7**	**2.5**
HSP104	**CA5135**	**7.5**	**1.0**	**8.4**
	Median	**1.8**	**4.8**	**6.1**

		**Heat Shock (°C)**			
		1	*2*	*3*	*Reference*
**Gene**		**23-37**	**30-42**	**30-45**	**Function**
CA4220	IPF8762	10.9	19.0	25.0	unknown function
CA4684	HSP78	1.9	6.9	13.5	heat shock protein of clpb family of ATP-dependent proteases
CA0828	IPF17186	2.7	3.8	12.5	unknown function
CA5135	HSP104	7.5	1.0	8.4	Heat shock protein (by homology)
CA1137	IFN1	2.6	3.2	7.9	glycerophosphoinositol transporter (by homology)
CA5613	IPF525	8.0	8.5	7.8	unknown function
CA5120	YDJ1	1.9	3.3	7.3	Mitochondrial and ER import protein (by homology)
CA0863	ITR2	1.5	2.5	6.8	Myo-inositol transporter (by homology)
CA0265	STI1		3.4	6.6	stress-induced protein (by homology)
CA2644	GRP2	4.2	1.2	5.9	Reductase (by homology)
CA3098	SIS1	2.2	3.1	5.8	heat shock protein (by homology)

CA3254	IPF4728	4.0	1.1	5.5	unknown Function
CA0558	GPX2	3.2	1.5	4.8	glutathione peroxidase (by homology)
CA6040	CPR6		5.1	4.6	cyclophylin (by homology)
CA0821	IPF16795	1.4	3.4	4.0	glycerate/formate-dehydrogenase (by homology)
CA0394	IPF12758	2.2	1.2	3.8	unknown function, 5-prime end
CA3886	IPF12963	2.1	1.7	3.8	ubiquitin-mediated protein degradation (by homology)
CA1075	IPF4991	2.2	1.0	3.8	putative membrane protein
CA2038	IPF17510	2.0	2.0	3.7	unknown function
CA4502	IPF10391	1.2	3.0	3.2	Similar to dnaJ proteins
CA4111	IPF3964	4.9	5.9	3.1	unknown function
CA1911	SSE1		2.2	2.8	heat shock protein of HSP70 family (by homology)
CA4162	MET1	1.3	2.6	2.7	siroheme synthase (by homology)
CA2857	SSA1		3.3	2.7	Heat shock protein of HSP70 family
CA4959	HSP90	1.4	2.7	2.5	heat shock protein
CA6002	IPF661	2.0	3.3	2.5	unknown function
CA1230	SSA4	5.1	11.4	2.5	cahsp70 mRNA for heat shock
CA3708	HIP1	0.8	2.4	2.4	Histidine permease (by homology)
CA4437	IPF13867	3.1	1.1	2.4	unknown function
CA2299	IPF8222	1.9	1.1	2.4	unknown function
CA5339	IPF885	3.0	1.0	2.4	glucan 1,3-beta-glucosidase (by homology)
CA2342	IPF13836	2.5	2.2	2.4	probable heat shock protein (by homology)
CA0915	KAR2		2.7	2.3	dnaK-type molecular chaperone (by homology)
CA6059	CTA26	2.1	1.9	2.3	transcriptional activation
CA2712	SPE2	2.2	1.4	2.3	Homologous to S. cerevisiae adenosylmethionine decarboxylase precurser
CA0896	SBA1	2.5	3.1	2.3	Hsp90 (Ninety) Associated Co-chaperone (by homology)
CA3334	ALP1	1.6	2.1	2.2	amino-acid permease (by homology)
CA0263	GLK1	2.1	0.7	2.2	aldohexose specific glucokinase (by homology)
CA1239	HSP60	1.4	3.3	2.1	Heat Shock Protein 60 (HSP60)
CA2474	PDC11	1.5	2.0	2.1	Pyruvate decarboxylase (by homology)
CA2854	RPN4	0.8	2.5	2.1	26S proteasome subunit (by homology)
CA5891	IPF2400	3.5	0.8	2.1	putative aldehyde reductase (by homology)

CA5817	IPF2511	1.3	1.0	2.1	unknown function
CA1497	IPF9683	2.1	1.1	2.1	unknown function
CA0602	CTA22	2.2	2.3	2.1	Protein with putative transcription activation domain
CA0037	IPF17652	2.6	0.9	2.0	reverse transcriptase, 3-prime end (by homology)
CA3367	IPF4667	8.2	1.3	1.1	Aromatic decarboxylase

4) *C. albicans* genes that were up-regulated in response heat shock in an Hsf1-dependent manner

Mean fold change is shown from >3 independent experiments

GENE			Fold Regulation			
			WT HS	hsf1 HS	WT/Hsf1	Function
CA4221	IPF8760	orf19.823	7.6	0.3	25.3	unknown function
CA4683	HSP78.3F	orf19.884	13.5	0.6	22.5	heat shock protein of clpb family of ATP-dependent proteases, mitochondrial, 3-prime end (by
CA4684	HSP78.5F	orf19.882	13.5	0.6	22.5	heat shock protein of clpb family of ATP-dependent proteases, mitochondrial, 5-prime end (by
CA0828	IPF17186	orf19.7882	12.5	1.1	11.4	unknown function
CA5613	IPF525	orf19.7085	7.8	0.7	11.1	unknown function
CA4739	IPF4498	orf19.4845	8.8	1.1	8.0	unknown function
CA2161	IPF11876	orf19.5295	6.9	0.9	7.7	unknown function
CA0265	STI1	orf19.10702	6.6	0.9	7.3	stress-induced protein (by homology)
CA0147	IPF9690	orf19.11133	6.3	0.9	7.0	unknown function
CA5135	HSP104	orf19.13747	6.0	0.9	6.7	Heat shock protein (by homology)
CA6002	IPF661	orf19.7602	2.5	0.4	6.3	unknown function
CA3098	SIS1	orf19.3861	5.8	1.0	5.8	heat shock protein (by homology)
CA5120	YDJ1	orf19.6408	7.3	1.3	5.6	Mitochondrial and ER import protein (by homology)
CA3367	IPF4667	orf19.9405	8.2	1.5	5.5	unknown Function
CA5029	IPF2997	orf19.6983	3.2	0.6	5.3	unknown function
CA6096	IPF56	orf19.5961	3.2	0.6	5.3	similar to Saccharomyces cerevisiae Nas6p subunit of 26S proteasome (by homology)
CA0924	IFD5	orf19.1048	2.1	0.4	5.3	Putative aryl-alcohol dehydrogenase (by homology)
CA2317	IPF13586	orf19.5479	3.1	0.6	5.2	unknown function
CA3254	IPF4728	orf19.11227	5.5	1.1	5.0	unknown Function
CA1137	IFN1	orf19.1979	7.9	1.6	4.9	glycerophosphoinositol transporter (by homology)
CA4437	IPF13867	orf19.5158	2.4	0.5	4.8	unknown function
CA6040	CPR6	orf19.7654	4.6	1.0	4.6	cyclophylin (by homology)
CA3966	IPF9376	orf19.1427	2.2	0.5	4.4	unknown function
CA3553	IFA16.5	orf19.3878	3.5	0.8	4.4	unknown function, 5-prime end
CA5848	IPF407	orf19.7504	3.5	0.8	4.4	unknown function
CA3886	IPF12963	orf19.5094	3.8	0.9	4.2	ubiquitin-mediated protein degradation (by homology)

CA3969	IPF9379	orf19.1430	4.2	1.0	4.2	unknown function
CA2038	IPF17510	orf19.11836	3.7	0.9	4.1	unknown function
CA5558	IPF1969	orf19.7322	3.7	0.9	4.1	unknown function
CA3803	IPF12407	orf19.2481	2.8	0.7	4.0	unknown function
CA5264	IPF1798	orf19.4998	2.7	0.7	3.9	unknown function
CA2039	IPF12947	orf19.11835	3.4	0.9	3.8	unknown function
CA1822	IPF9525	orf19.4268	2.2	0.6	3.7	unknown function
CA1966	IFR4	orf19.2394	2.5	0.7	3.6	unknown function
CA4757	LIP10	orf19.4822	3.2	0.9	3.6	Secretory lipase
CA2644	GRP2	orf19.4309	5.9	1.7	3.5	Reductase (by homology)
CA4111	IPF3964	orf19.675	3.1	0.9	3.4	unknown function
CA0821	IPF16795	orf19.2989	4.0	1.2	3.3	glycerate/formate-dehydrogenase (by homology)
CA0551	CDC37	orf19.5531	2.3	0.7	3.3	Cell division control protein (by homology)
CA4275	MDJ1	orf19.6672	4.5	1.4	3.2	Heat shock protein - chaperone (by homology)
CA2434	IPF9484	orf19.3396	4.7	1.5	3.1	unknown function
CA1230	SSA4	orf19.4980	2.5	0.8	3.1	cahsp70 mRNA for heat shock
CA2173	IPF18527	orf19.3764	3.0	1.0	3.0	unknown function
CA2857	SSA1	orf19.1065	2.7	0.9	3.0	Heat shock protein of HSP70 family
CA3730	IPF11900	orf19.4180	3.3	1.1	3.0	unknown function
CA1075	IPF4991	orf19.2531	3.8	1.3	2.9	putative membrane protein
CA1168	IPF13017	orf19.1785	2.1	0.7	2.9	unknown function
CA1911	SSE1	orf19.2435	2.8	1.0	2.8	heat shock protein of HSP70 family (by homology)
CA4959	HSP90	orf19.6515	2.5	0.9	2.8	heat shock protein
CA5325	IPF2138	orf19.7199	2.2	0.8	2.8	unknown function
CA4227	SCH9	orf19.829	2.2	0.8	2.8	strong similarity to S.pombe sck1 protein kinase (by homology)
CA3379	IPF8350	orf19.331	3.2	1.2	2.7	putative methyltransferase (by homology)
CA0150	CDC123	orf19.10236	2.4	0.9	2.7	similar to Saccharomyces cerevisiae Cdc123p cell cycle regulator (by homology)
CA2843	ALK5.5F	orf19.5728	2.1	0.8	2.6	n-alkane-inducible cytochrome P-450, 5-prime end
CA2854	RPN4	orf19.1069	2.1	0.8	2.6	26S proteasome subunit (by homology)
CA3545	BPT1.5F	orf19.6383	2.5	1.0	2.6	membrane transporter of the ATP-binding cassette (ABC) superfamily, 5-prime end (by homo
CA1908	APL3	orf19.2786	2.3	0.9	2.6	AP-2 complex subunit, alpha-adaptin (by homology)
CA2361	IPF8950	orf19.12363	2.3	0.9	2.6	unknown function

CA0915	KAR2	orf19.9564	2.3	0.9	2.6	dnaK-type molecular chaperone (by homology)
CA0037	IPF17652.3	orf19.6078	2.0	0.8	2.5	reverse transcriptase, 3-prime end (by homology)
CA1846	FDH12	orf19.638	2.7	1.1	2.5	Formate dehydrogenase (by homology)
CA0611	IPF17542	orf19.13024	2.2	0.9	2.4	unknown function
CA4602	IPF6231	orf19.10802	2.2	0.9	2.4	unknown function
CA5478	JEN2	orf19.12767	2.2	0.9	2.4	carboxylic acid transporter protein (by homology)
CA1138	IFN3	orf19.1978	3.9	1.6	2.4	glycerophosphoinositol transporter (by homology)
CA5339	IPF885	orf19.7214	2.4	1.0	2.4	glucan 1,3-beta-glucosidase (by homology)
CA2752	IPF6238	orf19.2374	2.7	1.1	2.4	GAG protein of retrotransposon pCal
CA2316	IPF14542	orf19.8963	2.0	0.8	2.4	unknown function
CA5552	IPF5971	orf19.7316	2.6	1.1	2.4	unknown function
CA2474	PDC11	orf19.2877	2.1	0.9	2.3	Pyruvate decarboxylase (by homology)
CA4480	IPF6464	orf19.1887	2.3	1.0	2.3	putative triacylglycerol lipase (by homology)
CA0169	RBT7	orf19.10196	2.3	1.0	2.3	repressed by TUP1
CA0896	SBA1	orf19.5749	2.3	1.0	2.3	Hsp90 (Ninety) Associated Co-chaperone (by homology)
CA2562	IPF19953	orf19.5642	2.5	1.1	2.3	unknown function
CA2594	IPF12824	orf19.57	2.2	1.0	2.2	unknown function
CA5601	IPF556	orf19.7098	2.6	1.2	2.2	transcriptional regulator (by homology)
CA0602	CTA22	orf19.3074	2.1	1.0	2.1	Protein with putative transcription activation domain
CA3551	IPF13229	orf19.3876	2.3	1.1	2.1	unknown function
CA1829	IPF17177.3F	orf19.5184	2.3	1.1	2.1	similar to Saccharomyces cerevisiae Srm1p regulator of chromosome condensation, 3-prime
CA3405	IPF8644	orf19.3982	2.5	1.2	2.1	maltase (by homology)
CA5536	IPF2837	orf19.7297	3.7	1.8	2.1	putative cystathionine gamma-synthase (by homology)
CA2895	IFS1	orf19.2461	2.2	1.1	2.0	Unknown function
CA4502	IPF10391	orf19.8136	3.2	1.6	2.0	Similar to dnaJ proteins
CA1795	IPF11261	orf19.5209	2.4	1.2	2.0	unknown function
CA1630	RPP1	orf19.1029	2.2	1.1	2.0	required for processing of tRNA and 35S rRNA (by homology)
CA2433	IPF12959	orf19.3399	2.3	1.2	1.9	unknown function
CA2130	RPS27A		2.3	1.2	1.9	ribosomal protein S27.e (by homology)
CA5950	TPI1	orf19.6745	2.3	1.2	1.9	Triose phosphate isomerase
CA5891	IPF2400	orf19.6816	2.1	1.1	1.9	putative aldehyde reductase (by homology)
CA5847	CDA2	orf19.7503	2.4	1.3	1.8	chitin deacetylase (by homology)

CA0263	GLK1	orf19.1408	2.2	1.2	1.8	aldohexose specific glucokinase (by homology)
CA1809	IPF6325	orf19.1116	3.3	1.8	1.8	unknown function
CA6128	IPF152	orf19.5919	2.1	1.2	1.8	unknown function
CA3625	CYR1.3F	orf19.12617	2.3	1.3	1.8	adenylate cyclase, 3-prime end
CA4934	CDC36	orf19.6075	2.0	1.1	1.8	transcription factor (by homology)
CA5266	IPF1787.3F	orf19.4996	2.0	1.1	1.8	unknown function, 3-prime end
CA4473	IPF6447	orf19.1897	2.0	1.1	1.8	unknown function
CA3964	IPF9370	orf19.1424	2.7	1.5	1.8	unknown function
CA6059	CTA26	orf19.7680	2.3	1.3	1.8	transcriptional activation
CA6054	IPF4952	orf19.7672	2.1	1.2	1.8	unknown function
CA2218	IPF18508	orf19.5375	2.4	1.4	1.7	unknown function
CA5480	IPF8210	orf19.5312	2.2	1.3	1.7	unknown function
CA2756	IPF18418	orf19.13065	2.0	1.2	1.7	unknown function
CA4593	IPF2214	orf19.6910	2.0	1.2	1.7	unknown function
CA6003	IPF660	orf19.7603	2.0	1.2	1.7	unknown function
CA1773	VRP1	orf19.2190	2.3	1.4	1.6	verprolin (by homology)
CA1239	HSP60	orf19.717	2.1	1.3	1.6	Heat Shock Protein 60 (HSP60)
CA5602	IPF554	orf19.7097	2.1	1.3	1.6	RNA binding protein (by homology)
CA1497	IPF9683	orf19.3644	2.1	1.3	1.6	unknown function
CA3951	VAC7.3	orf19.1409	2.0	1.3	1.5	Vacuolar protein, 3-prime end (by homology)
CA2342	IPF13836	orf19.2344	2.4	1.6	1.5	probable heat shock protein (by homology)
CA2299	IPF8222	orf19.5381	2.4	1.6	1.5	unknown function
CA0796	ALR1	orf19.9175	2.8	1.9	1.4	divalent cation transporter (by homology)
CA4581	IPF2232	orf19.6899	2.4	1.8	1.3	unknown function
CA2338	NIT3	orf19.2351	2.0	1.5	1.3	nitrilase (by homology)
CA5846	IPF409	orf19.7502	2.1	1.8	1.2	unknown function
CA1399	IPF8069	orf19.3698	2.0	1.9	1.1	unknown function

5) *C. albicans* genes that displayed Hsf1-dependence under basal conditions

Mean fold change is shown from ≥3 independent experiments

GENE			Reg	Function
CA2191	IPF9417	orf19.4775	0.2	similar to Saccharomyces cerevisiae Hsf1p heat shock transcription factor (by homology)
CA0828	IPF17186	orf19.7882	0.2	unknown function
CA4030	EBP4	orf19.3433	0.3	NADPH dehydrogenase (by homology)
CA0583	GSH1.3f	orf19.12526	0.3	gamma-glutamylcysteine synthetase, exon 2 (by homology)
CA6002	IPF661	orf19.7602	0.4	unknown function
CA0584	GSH1.5f	orf19.12527	0.4	Gamma-glutamylcysteine synthetase, exon 1 (by homology)
CA6040	CPR6	orf19.7654	0.4	cyclophylin (by homology)
CA5029	IPF2997	orf19.6983	0.4	unknown function
CA1290	IPF16212	orf19.2710	0.5	unknown function
CA2644	GRP2	orf19.4309	0.5	Reductase (by homology)
CA1333	MRF1	orf19.8742	0.5	mitochondrial respiratory function protein (by homology)
CA4921	IPF1416	orf19.6061	0.5	unknown function
CA2554	CIRT4B	orf19.2839	0.5	probable transposase (by homology)
CA4940	CRN1.3f	orf19.6535	0.5	actin-binding protein, 3-prime end (by homology)
CA3756	IPF8884	orf19.3422	0.5	unknown function
CA0551	CDC37	orf19.5531	0.5	Cell division control protein (by homology)
CA1593	MIG1	orf19.4318	0.5	transcriptional regulator
CA3367	IPF4667	orf19.9405	0.5	unknown Function
CA3098	SIS1	orf19.3861	0.5	heat shock protein (by homology)
CA2522	GCV1	orf19.5519	0.5	glycine cleavage T protein (by homology)
CA5973	IPF931	orf19.7567	0.5	unknown function
CA2349	IFC3	orf19.3749	0.5	Unknown function
CA2314	IPF14545	orf19.1381	0.5	unknown function
CA0265	STI1.3f	orf19.10702	0.5	stress-induced protein (by homology)
CA4516	IPF18207	orf19.489	0.5	unknown function

CA1971	PKH2	orf19.5224	0.5	Ser/Thr protein kinase(by homology)
CA3637	IPF9255	orf19.5136	0.5	unknown function
CA4683	HSP78	orf19.884	0.5	heat shock protein of clpb family of ATP-dependent proteases, mitochondrial, 3-prime enc
CA1956	ERG3	orf19.767	0.5	C5,6 desaturase
CA0945	IPF10278	orf19.8853	0.5	DNA-J - like protein (by homology)
CA3257	IFC1	orf19.3746	0.5	Unknown Function
CA4163	IPF9301	orf19.5812	0.5	unknown function
CA0210	IPF4328	orf19.12971	0.5	unknown function
CA2938	IPF8321	orf19.3325	0.5	similar to Saccharomyces cerevisiae Glg2p self-glucosylating initiator of glycogen synthes
CA5201	IPF2349	orf19.7016	0.6	similar to human sphingomyelin
CA1113	IPF9996	orf19.2285	0.6	unknown function
CA5749	IPF2908	orf19.7459	0.6	unknown function
CA3014	IPF11915	orf19.13605	0.6	similar to Saccharomyces cerevisiae Pcl7p cyclin like protein interacting with Pho85p (by [illegible]
CA0086	IPF16598	orf19.7781	0.6	unknown function
CA0548	PHO87	orf19.2454	0.6	Member of the phosphate permease family (by homology)
CA4261	PDX3	orf19.550	0.6	pyridoxamine-phosphate oxidase (by homology)
CA4772	CCT3	orf19.4004	0.6	Chaperonin (by homology)
CA3578	IPF11105	orf19.2262	0.6	probable quinone oxidoreductase
CA6135	CMK1	orf19.5911	0.6	Ca2+/calmodulin-dependent ser/thr protein kinase (by homology)
CA3061	IPF2965	orf19.11763	0.6	unknown function
CA5984	CUS1	orf19.7581	0.6	spliceosome associated protein (by homology)
CA3029	IPF13825	orf19.223	0.6	similarity to serine/threonine protein kinases (by homology)
CA2822	IPF14550	orf19.1314	0.6	unknown function
CA3564	IPF7817	orf19.3131	0.6	putative NADH-dependent flavin oxidoreductase (by homology)
CA1548	IPF8746	orf19.4279	0.6	putative alpha-1,3-mannosyltransferase (by homology)
CA4437	IPF13867	orf19.5158	0.6	unknown function
CA3918	VTC2	orf19.4381	0.6	putative polyphosphate synthetase (by homology)
CA6057	IPF4959	orf19.7676	0.6	D-xylulose reductase (by homology)
CA0984	IPF7400	orf19.1802	0.6	unknown function
CA2948	GDS1	orf19.1963	0.6	nam9-1 suppressor (by homology)
CA0147	IPF9690	orf19.11133	0.6	unknown function

CA0724	IPF13398	orf19.7708	0.6	protein kinase (by homology)
CA2597	IFQ3	orf19.54	0.6	unknown function
CA5824	NTH1	orf19.7479	0.6	Neutral trehalase
CA3078	IPF19970	orf19.4816	0.6	unknown function
CA1865	IPF7646	orf19.861	0.6	putative transcription factor (by homology)
CA3260	IPF7968	orf19.2693	0.6	unknown function
CA4010	MAK10.3f	orf19.1624	0.6	glucose-repressible protein, 3-prime end (by homology)
CA4780	IPF3352	orf19.4013	0.6	unknown function
CA5907	SSN6	orf19.6798	0.6	transcriptional repressor (by homology)
CA5135	HSP104	orf19.13747	0.6	Heat shock protein (by homology)
CA0353	YAK1.3f	orf19.147	0.6	serine/threonine protein kinase, 3-prime end (by homology)
CA1111	RIB3.5f	orf19.12693	0.6	3,4-dihydroxy-2-butanone 4-phosphate synthase (by homology)
CA1439	IPF19602	orf19.4488	0.6	similar to Saccharomyces cerevisiae Swi3p transcription regulatory protein (by homology)
CA1353	ERG1	orf19.406	0.6	squalene epoxidase
CA4621	IPF5753	orf19.3312	0.6	unknown function
CA4553	CYB1	orf19.1446	0.6	G2/Mitotic-specific cyclin
CA1642	NHP1.5f	orf19.1730	0.6	unknown function, 5-prime end
CA3869	IPF8831	orf19.400	0.6	unknown function
CA1851	IPF14629	orf19.642	0.6	Cell cycle protein, interacts with Sit4 (by homology)

6) *C. albicans* genes that were up-regulated by heat shock only following Hsf1 depletion

Functional categories significantly enriched in this subset of 171 *C. albicans* genes

GO ID	GO_term	Corrected P-value
6790	sulfur metabolic process	3.00E-04
9092	homoserine metabolic process	2.66E-03
9067	aspartate family amino acid biosynthetic process	4.12E-02
6811	ion transport	5.80E-02
34470	ncRNA processing	8.45E-02

Gene		WT HS	hsf1 HS	Function	orf #
CA6036	IPF585	1.9	5.1	unknown function	orf19.7646
CA1760	IPF17914	1.0	4.4	unknown function	orf19.5124
CA5404	MET14	1.0	3.9	Adenylylsulfate kinase (by homology)	orf19.946
CA4693	IPF3952	0.9	3.6	unknown function	orf19.872
CA2205	SEO2	0.7	3.4	suppressor of sulfoxyde ethionine resi	orf19.700
CA4598	IPF20019	1.2	3.4	unknown function	orf19.6917
CA1568	IPF7615	1.5	3.3	unknown function	orf19.1158
CA1666	SRP40	1.3	3.3	RNA I and II supressor (by homology)	orf19.2859
CA1694	TRM3	0.9	3.2	2 -O-ribose methyltransferase (by hom	orf19.5038
CA1848	IPF11128	1.1	3.2	unknown function	orf19.639.1
CA2440	IPF7513	1.5	3.2	unknown function	orf19.2196
CA5161	IPF983	0.7	3.2	unknown function	orf19.4600
CA1365	IPF11849	1.4	3.1	unknown function	orf19.4459
CA0384	PCL1	1.3	3.0	cyclin, G1/S-specific (by homology)	orf19.2649
CA5370	IPF5248	1.7	3.0	unknown function	orf19.7254
CA5943	IPF3490	0.9	3.0	unknown function	orf19.6754
CA2335	LYS21	1.3	2.9	homocitrate synthase (by homology)	orf19.4506
CA3160	ZRT2	1.6	2.9	zinc transport protein (by homology)	orf19.1585
CA5238	MET3	1.2	2.9	ATP sulfurylase	orf19.5025
CA6028	IPF607	1.2	2.9	unknown function	orf19.7634
CA1963	IPF14040	0.7	2.8	probable transporter (by homology)	orf19.2397
CA0136	IFH1	1.1	2.7	Dioxygenase (by homology)	orf19.1639
CA1751	ARO3	1.7	2.7	3-deoxy-D-arabinoheptulosonate-7-ph	orf19.1517
CA2072	IPF6970	1.2	2.7	unknown function	orf19.2639
CA2791	IMP4	0.9	2.7	Ribonucleoprotein (by homology)	orf19.603
CA2946	IPF6296	1.0	2.7	putative methyltransferase (by homolo	orf19.1966
CA4114	IPF7704	1.4	2.7	unknown function	orf19.679
CA4183	SNO1	1.0	2.7	hisH-like protein (by homology)	orf19.2948
CA5115	IPF1331	1.3	2.7	unknown function	orf19.6416
CA5670	IPF1266	1.8	2.7	Probable transcription factor	orf19.7372
CA1228	IPF16081	1.3	2.6	unknown function	orf19.4161
CA1501	IPF13217	1.2	2.6	unknown function	orf19.1698
CA1569	IPF7616	1.0	2.6	putative homoserine O-acetyltransfera	orf19.1159
CA1747	IPF10884	1.0	2.6	unknown function	orf19.5049
CA1806	IPF6329	1.9	2.6	unknown function	orf19.1113
CA2004	POL12	1.0	2.6	DNA-directed DNA polymerase alpha	orf19.2796
CA4461	IPF8470	1.6	2.6	unknown function	orf19.6858
CA4825	IPF1205	1.3	2.6	unknown function	orf19.2071
CA5471	IPF302	1.3	2.6	short chain dehydrogenase/reductase	orf19.3283
CA5668	IPF1261	1.1	2.6	unknown function	orf19.7370
CA0123	MXR1	1.5	2.5	methionine sulfoxide reductase	orf19.2028
CA0167	IPF19160	1.1	2.5	unknown function	orf19.1075
CA1226	IPF15660	0.9	2.5	putative mitochondrial carrier (by hom	orf19.4159
CA4429	DBF4	1.5	2.5	regulatory subunit for Cdc7 by homolo	orf19.5166
CA4612	PPM2	1.0	2.5	carboxy methyl transferase	orf19.3303
CA4966	IPF3923	0.6	2.5	unknown function	orf19.6507
CA5328	RER1	1.5	2.5	Required for correct localization of Se	orf19.7202
CA5827	IPF2485	1.9	2.5	unknown function	orf19.7482
CA5919	IPF8926.3	1.4	2.5	unknown function, 3-prime end	orf19.6786
CA0309	IPF16935	1.0	2.4	unknown function	orf19.1091
CA1097	IPF7575	0.9	2.4	putative endo-exonuclease (by homolo	orf19.4365

CA1921	IPF7475	1.6	2.4	similar to Saccharomyces cerevisiae C	orf19.2444
CA2144	IPF12802	1.4	2.4	unknown function	orf19.2513
CA2484	IPF9846	1.2	2.4	unknown function	orf19.3202
CA3131	IPF8910	0.9	2.4	unknown function	orf19.1362
CA4404	MET16	1.6	2.4	3 -phosphoadenylylsulfate reductase (	orf19.3106
CA5844	PXA1	0.9	2.4	long chain fatty acid ABC transporter (	orf19.7500
CA0234	IFA3	1.7	2.3	unknown function	orf19.154
CA0889	IPF11767	1.3	2.3	mitochondrial carrier protein (by homo	orf19.4733
CA1461	IPF6338	1.3	2.3	unknown function	orf19.1109
CA1580	TRP5	1.7	2.3	tryptophan synthase (by homology)	orf19.4718
CA1658	IPF19924	0.9	2.3	unknown function	orf19.4324
CA1693	IPF17054	0.9	2.3	unknown function	orf19.5037
CA1808	GUK1	1.4	2.3	Guanylate kinase (by homology)	orf19.1115
CA2358	IPF8953	1.1	2.3	unknown function	orf19.4895
CA2565	MET15	1.7	2.3	O-acetylhomoserine O-acetylserine su	orf19.5645
CA2684	IFU3	1.4	2.3	Unknown function	orf19.2575
CA4039	SKS1	0.8	2.3	serine/threonine kinase by homology	orf19.3669
CA4323	IPF2096	0.7	2.3	putative acyltransferase (by homology)	orf19.4096
CA5127	CYS3	1.5	2.3	cystathionine gamma-lyase by homolo	orf19.6402
CA5133	IPF5129	1.5	2.3	unknown function	orf19.6392
CA5673	IPF1272	1.3	2.3	unknown function	orf19.7376
CA5708	MRPL39	1.1	2.3	Mitochondrial 60S ribosomal protein (b	orf19.7409.1
CA0011	IPF17430	1.0	2.2	possible zinc protease (by homology)	orf19.73
CA0027	RCL1	0.8	2.2	RNA 3 -terminal phosphate cyclase (b	orf19.1886
CA0302	MEP3	1.6	2.2	low affinity high capacity ammonium p	orf19.1614
CA0512	VMA16	1.2	2.2	H+-ATPase 23 KD subunit, vacuolar (b	orf19.4954
CA1369	IPF15968	1.7	2.2	unknown function	orf19.4964
CA1649	LAB1	1.4	2.2	Lipoate biosynthesis by homology	orf19.5566
CA2117	SNG3	0.9	2.2	Drug transporter (by homology)	orf19.1333
CA2824	IPF17888	1.0	2.2	unknown function	orf19.1318
CA2913	IPF11205	1.7	2.2	unknown function	orf19.4513
CA3046	IPF7295	1.1	2.2	unknown function	orf19.2417
CA3270	LYS5	1.2	2.2	L-aminoadipate-semialdehyde dehydr	orf19.6304
CA3363	FRE32	0.9	2.2	ferric reductase (by homology)	orf19.1932
CA3611	IPF3853	1.1	2.2	unknown function	orf19.28
CA4097	GIN4	1.2	2.2	ser/thr protein kinase (by homology)	orf19.663
CA4116	IDI1	1.1	2.2	isopentenyl-diphosphate delta-isomera	orf19.2775
CA4174	IPF4119.5	1.3	2.2	unknown function, 5-prime end	orf19.2959.1
CA4714	IPF5912	1.8	2.2	unknown function	orf19.6950
CA4779	IPF3351	1.9	2.2	unknown function	orf19.4012
CA4814	SAS2	1.3	2.2	Zinc finger protein involved in silencing	orf19.2087
CA4914	IPF1428	1.6	2.2	Similar to ubiquitination protein Bul1p (	orf19.6054
CA4945	FLX1	0.9	2.2	MITOCHONDRIAL FAD CARRIER by	orf19.6532
CA5118	IPF1321	1.3	2.2	unknown function	orf19.6413
CA5344	PRY2	1.1	2.2	putative pathogen related proteins (by	orf19.7218
CA6042	IPF4924	1.1	2.2	unknown function	orf19.7657
CA6043	RFC4	1.1	2.2	DNA replication factor C (by homology	orf19.7658
CA6116	TOP3	1.2	2.2	DNA topoisomerase III (by homology)	orf19.5934
CA0067	IPF13904	1.4	2.1	farnesyl cysteine carboxyl-methyltrans	orf19.120
CA0964	IPF9141	1.1	2.1	similar to Saccharomyces cerevisiae C	orf19.6247
CA1019	IPF13485	1.1	2.1	unknown function	orf19.3769
CA1161	GRX3	1.7	2.1	glutaredoxin-like protein	orf19.2727

CA1540	IPF1047	1.0	2.1	unknown function	orf19.4563
CA1566	DPP2	1.2	2.1	Diacylglycerol pyrophosphate phospha	orf19.1155
CA1616	IPF14538	1.6	2.1	unknown function	orf19.3219
CA1724	MRP10	1.1	2.1	Mitochondrial ribosomal protein (by ho	orf19.2650.1
CA1746	IPF10886	1.2	2.1	unknown function	orf19.5050
CA2167	IPF4553	1.0	2.1	unknown function	orf19.909
CA2183	IPF6872	1.2	2.1	serine/threonine protein kinase (by hor	orf19.4252
CA2295	HOS2	1.1	2.1	putative histone deacetylase (by homo	orf19.5377
CA2449	ENP1	1.0	2.1	Essential nuclear protein (by homology	orf19.5507
CA2945	IMG1	1.4	2.1	Ribosomal protein, mitochondrial (by h	orf19.1967
CA3139	YHB3	1.0	2.1	flavohemoglobin (by homology)	orf19.3710
CA3303	FRE42	1.3	2.1	ferric reductase (by homology)	orf19.2312
CA3340	ADA2	1.3	2.1	general transcriptional adaptor or co-a	orf19.2331
CA3432	MRPL17	0.9	2.1	ribosomal protein of the large subunit	orf19.585
CA3558	IPF7760	1.0	2.1	unknown function	orf19.3887
CA3996	IPF10440	1.0	2.1	unknown function	orf19.1643
CA4320	ECM17	0.8	2.1	Putative sulfite reductase (by homolog	orf19.4099
CA4400	IPF14362	1.2	2.1	unknown function	orf19.3110
CA4627	DUT1	1.5	2.1	dUTP pyrophosphatase	orf19.3322
CA4715	DPL1	1.7	2.1	dihydrosphingosine phosphate lyase (	orf19.6951
CA4809	IPF1162	1.7	2.1	Cystathionine beta-lyase (by homology	orf19.2092
CA4962	TRL1	1.4	2.1	tRNA ligase	orf19.6511
CA4968	IPF3928	1.1	2.1	unknown function	orf19.6503
CA5004	TRP3	1.4	2.1	Anthranilate synthase / indole glycerol	orf19.5243
CA5347	IPF864	1.1	2.1	unknown function	orf19.7222
CA5474	IPF16944.3EOC	1.0	2.1	unknown function, 3-prime end	orf19.3287
CA0083	PHO84	1.4	2.0	high-affinity inorganic phosphate/H+ sy	orf19.655
CA0161	RMS1	1.3	2.0	(putative) transcriptional regulator (by	orf19.2654
CA0197	IPF9787	0.9	2.0	similar to Saccharomyces cerevisiae H	orf19.1934
CA0275	NTA1	1.8	2.0	Amino-terminal amidase (by homology	orf19.850
CA0361	IPF2326	0.8	2.0	unknown function	orf19.1124
CA0381	IPF16047	1.3	2.0	unknown function	orf19.2825
CA0534	IPF15607	0.8	2.0	unknown function	orf19.5066
CA0617	MET2	1.4	2.0	Homoserine O-acetyltransferase	orf19.2618
CA0627	HSP12	9.8	2.0	Heat shock protein (by homology)	orf19.3160
CA0723	PRI2	0.8	2.0	DNA-directed DNA polymerase alpha	orf19.2885
CA0774	IPF11974	0.8	2.0	unknown function	orf19.855
CA1058	DOT6	1.0	2.0	involved in derepression of telomeric	orf19.2545
CA1153	IPF10455	1.5	2.0	unknown function	orf19.104
CA1648	IPF16662	1.1	2.0	unknown function	orf19.5567
CA1701	CFL11	1.3	2.0	Ferric reductase (by homology)	orf19.701
CA1745	IPF10888	1.1	2.0	unknown function	orf19.5051
CA2005	IPF11802	1.2	2.0	unknown function	orf19.2797
CA2266	HEM1	1.0	2.0	5-aminolevulinic acid synthase	orf19.2601
CA2374	FCY23	1.2	2.0	Putative purine-cytosine transport prot	orf19.1832
CA2545	RRP9	1.2	2.0	U3 small nucleolar ribonucleoprotein-a	orf19.2830
CA2629	IPF10911	0.8	2.0	unknown function	orf19.4760
CA3213	IPF8160	1.6	2.0	unknown function	orf19.6205
CA3349	IPF19588	1.3	2.0	unknown function	orf19.2318.1
CA3497	FEN11	1.1	2.0	Fatty acid elongase required for sphin	orf19.6343
CA3939	HNM1	0.8	2.0	Choline permease (by homology)	orf19.2003
CA4062	IPF5933	1.1	2.0	similar to Saccharomyces cerevisiae M	orf19.439

CA4574	IPF9430	1.6	2.0	similar to Saccharomyces cerevisiae S	orf19.6885
CA4613	IPF7950	1.1	2.0	similar to Saccharomyces cerevisiae F	orf19.3304
CA4952	IPF13609	1.0	2.0	unknown function	orf19.6526
CA5044	GTT2	1.0	2.0		orf19.6998
CA5153	IPF1003	1.9	2.0	unknown function	orf19.4592
CA5198	LPA4	1.2	2.0	Similar to ribosomal protein S16, mitoc	orf19.7012
CA5223	IPF3080	1.4	2.0	unknown function (by homology)	orf19.7042
CA5320	IPF2147	1.5	2.0	unknown function	orf19.7194
CA5472	IPF16948	1.3	2.0	unknown function	orf19.3285
CA5526	IPF2857	1.9	2.0	unknown function	orf19.7284
CA5545	IPF5986	0.9	2.0	similar to cytochrome-b5- and nitrate r	orf19.7307
CA5710	OAC1	0.8	2.0	Mitochondrial oxaloacetate transport p	orf19.7411
CA5786	IPF1134	1.5	2.0	unknown function	orf19.5433
CA6044	IPF4928	1.2	2.0	similar to Saccharomyces cerevisiae S	orf19.7660
CA6083	IPF26	1.2	2.0	unknown function	orf19.5976
CA6104	IPF65	1.0	2.0	unknown function	orf19.5952

7) *C. albicans* genes that were down-regulated by heat shock only following Hsf1 depletion

Functional categories significantly enriched in this subset of 385 *C. albicans* genes

GO ID	GO_term	Corrected P-value
5984	disaccharide metabolic process	9.19E-02

Gene		WT HS	hsf1 HS	Function	orf #
CA0413	ALS12.3f	0.9	0.1	agglutinin-like protein, 3-prime en	orf19.1097
CA1471	ALS2	0.9	0.1	agglutinin-like protein, 3-prime en	orf19.1097
CA2832	CRD1	1.1	0.1	Cu-transporting P1-type ATPase	orf19.4784
CA0495	IPF20056	0.7	0.2	unknown function	orf19.1354
CA1244	IPF12767	0.8	0.2	unknown function	orf19.2624
CA1528	ALS4.3f	0.9	0.2	agglutinin-like protein, 3-prime en	orf19.4555
CA2261	RAS1	0.6	0.2	GTP-binding protein (by homolog	orf19.1760
CA2738	STF2	1.0	0.2	ATP synthase regulatory factor (I	orf19.2107.1
CA2822	IPF14550	1.0	0.2	unknown function	orf19.1314
CA4220	IPF8762	25.0	0.2	unknown function	orf19.822
CA4266	IPF2283	1.3	0.2	unknown function	orf19.6660
CA0127	HXK2	1.0	0.3	hexokinase II, 3-prime end (by ho	orf19.542
CA0212	IPF4325	0.9	0.3	unknown function	orf19.5523
CA0553	IPF16653	0.7	0.3	unknown function	orf19.5534
CA0601	MSN5.3f	0.7	0.3	Importin-beta family member req	orf19.2665
CA0692	PGM2	0.9	0.3	Phosphoglucomutase (by homolo	orf19.2841
CA0824	GPD2	0.7	0.3	Glycerol 3-phosphate dehydroge	orf19.691
CA0943	YHB1	0.7	0.3	flavohemoglobin (by homology)	orf19.3707
CA1069	HXT5.3f	0.8	0.3	sugar transporter, 3-prime end	orf19.2021
CA1431	FET34	0.3	0.3	Sec9 interacting protein (by hom	orf19.4215
CA1542	ROT11	1.9	0.3	Putative membrane protein	orf19.4566
CA1574	PXP2	0.9	0.3	acyl-CoA oxidase peroxisomal (b	orf19.1655
CA1618	RRP45	0.7	0.3	Protein component of the exosor	orf19.4078
CA1810	FDH2	1.1	0.3	Formate dehydrogenase (by hom	orf19.1117
CA2215	PFK26	0.7	0.3	6-phosphofructose-2-kinase (by I	orf19.4753
CA2334	ADH3.3f	1.0	0.3	probable alcohol dehydrogenase	orf19.4505
CA2554	Cirt4	0.9	0.3	probable transposase (by homolo	orf19.2839
CA2555	IPF12105	0.8	0.3	unknown function	orf19.5633
CA2855	GPM2	0.7	0.3	phosphoglycerate mutase (by ho	orf19.1067
CA2920	FET5	0.3	0.3	multicopy oxidase (by homology)	orf19.4215
CA3679	IPF20142	0.8	0.3	unknown function	orf19.729.1
CA4069	MHP1	0.6	0.3		orf19.6621
CA4123	AMS1	0.8	0.3	alpha-mannosidase (by homolog	orf19.2768
CA4221	IPF8760	7.6	0.3	unknown function	orf19.823
CA4611	IPF10727	0.8	0.3	unknown function	orf19.3302
CA4850	Cirt2	1.4	0.3	Transposase	orf19.3820
CA4940	CRN1	0.7	0.3	actin-binding protein, 3-prime en	orf19.6534.2
CA5143	IPF1020	1.3	0.3	Weak similarity to N. crassa hypo	orf19.4580
CA5467	GSY1	0.8	0.3	UDP glucose--starch glucosyltrar	orf19.3278
CA5788	RHR2	3.2	0.3	DL-glycerol phosphatase	orf19.5437
CA5912	IPF5966	1.1	0.3	unknown function	orf19.6793
CA6057	IPF4959	1.9	0.3	D-xylulose reductase (by homolo	orf19.7676
CA6102	SPO70	1.2	0.3	involved in meiosis and sporulati	orf19.5954
CA0069	IPF19295	1.0	0.4	unknown function, internal fragm	orf19.6469
CA0070	IPF19295	1.0	0.4	unknown function, 3-prime end	orf19.6469
CA0071	IPF19290	0.9	0.4	unknown function, 3-prime end	orf19.6469
CA0175	NPL4	1.4	0.4	nuclear protein localization factor	orf19.2434
CA0338	IPF13252	1.0	0.4	unknown function	orf19.3378
CA0341	XKS1	1.2	0.4	xylulokinase (by homology)	orf19.1290
CA0386	IPF4065	0.7	0.4	unknown function	orf19.1862

CA0442	IFC4	0.8	0.4	unknown function	orf19.176
CA0516	IPF11526	1.1	0.4	unknown function	orf19.2804
CA0517	HEM13	1.0	0.4	by homology S. cerev.: copropor	orf19.2803
CA0917	RAD16	1.0	0.4	nucleotide excision repair protein	orf19.2969
CA0924	IFD5	2.1	0.4	Putative aryl-alcohol dehydrogen	orf19.1048
CA1255	IPF9030	0.7	0.4	unknown function	orf19.1776
CA1334	IPF19912	0.9	0.4	unknown function	orf19.1148
CA1353	ERG1	1.2	0.4	squalene epoxidase	orf19.406
CA1359	IPF11842	0.8	0.4	unknown function	orf19.4405
CA1446	YAL011	1.2	0.4	mitochondrial transit peptide (by	orf19.190
CA1879	SMF12	1.4	0.4	manganese transporter (by homo	orf19.2270
CA1898	PGA38	0.8	0.4	unknown function	orf19.2758
CA1956	ERG3	0.7	0.4	C5,6 desaturase	orf19.767
CA1957	IPF3887	1.1	0.4	similar to Saccharomyces cerevis	orf19.768
CA1958	IFE1	1.1	0.4	Unknown function	orf19.769
CA1962	IPF14285	0.8	0.4	unknown function	orf19.2398
CA2017	IPF3414	0.9	0.4	putative serine/threonine protein	orf19.846
CA2075	IFE2	0.9	0.4	Unknown function	orf19.5288
CA2210	MSL1	0.9	0.4	U2 snRNA-associated protein (by	orf19.4748
CA2225	SUR2	2.3	0.4	Hydroxylation of C-4 of the sphin	orf19.5818
CA2315	YEA4	1.4	0.4	Golgi uridine diphosphate-N-acet	orf19.1382
CA2601	IPF11181	1.0	0.4	unknown function	orf19.50
CA2873	AQY1	0.9	0.4	similarity to plasma membrane a	orf19.2849
CA2875	MEI5	1.1	0.4	meiotic protein (by homology)	orf19.5844
CA3112	PFK2	2.0	0.4	6-phosphofructokinase, beta sub	orf19.6540
CA3598	SEC2	0.7	0.4	GDP/GTP exchange factor (by h	orf19.4928
CA3643	IPF4124	1.1	0.4	unknown function	orf19.2034
CA3684	IPF13613	1.1	0.4	unknown function	orf19.725
CA3720	IFL3	0.8	0.4	Unknown function	orf19.4170
CA3745	MAF1	0.8	0.4	nuclear protein by homology	orf19.2173
CA3766	IPF18298	0.9	0.4	unknown function, 3-prime end	orf19.2903
CA3895	CDR4	0.7	0.4	Multidrug resistance protein	orf19.5079
CA4189	IPF7527	1.1	0.4	unknown function	orf19.4530.1
CA4442	IPF3537	0.7	0.4	unknown function	orf19.6838
CA4444	IPF3533	0.7	0.4	putative GDP/GTP exchange fac	orf19.6842
CA4570	IPF9550	1.6	0.4	similar to Saccharomyces cerevis	orf19.6882
CA4716	IPF8666	0.7	0.4	unknown function	orf19.6952
CA4735	IPF7726	0.8	0.4	unknown function	orf19.4850
CA4827	SMF2	1.0	0.4	Manganese transporter (by homo	orf19.2069
CA5066	TPS2	1.0	0.4	Threalose-6-phosphate phospha	orf19.3038
CA5268	SEC18.3f	1.1	0.4	vesicular fusion protein, 3-prime	orf19.4993
CA5270	SEC18.5f	1.1	0.4	vesicular fusion protein by homol	orf19.4993
CA5322	PRB1	0.7	0.4	Protease B, vacuolar (by homolo	orf19.7196
CA5337	APL1	1.1	0.4	AP-2 complex subunit, beta2-ada	orf19.7212
CA5694	IPF3311	1.3	0.4	unknown function	orf19.7396
CA5766	IPF1097	1.0	0.4	serine/threonine protein kinase (l	orf19.5408
CA5824	NTH1	1.0	0.4	Neutral trehalase	orf19.7479
CA6002	IPF661	2.5	0.4	unknown function	orf19.7602
CA6008	PGA11	0.8	0.4	unknown function	orf19.7609
CA6084	IPF29	0.9	0.4	zinc finger protein (by homology)	orf19.5975
CA0005	IPF14994	1.2	0.5	unknown function	orf19.2414
CA0052	IPF19567	0.6	0.5	Unknown function	orf19.475

CA0106	IPF16308	0.9	0.5	unknown function	orf19.3851
CA0185	PLB4.5f	0.7	0.5	Phospholipase, 5-prime end (by	orf19.1442
CA0262	IPF20054	1.5	0.5	unknown function	orf19.6117
CA0282	IPF17417	1.0	0.5	Unknown function	orf19.4691
CA0295	IPF15033.5eoc	1.1	0.5	unknown function	orf19.1595
CA0303	IPF17507	1.4	0.5	putative glutathione S-transferas	orf19.720
CA0397	FRE31	1.2	0.5	Ferric reductase (by homology)	orf19.1930
CA0546	IPF3250	1.5	0.5	unknown function	orf19.2870
CA0571	CDC43	1.2	0.5	geranylgeranyltransferase I	orf19.1803
CA0609	CDR11.3f	1.0	0.5	multidrug resistance protein, 3-pr	orf19.918
CA0610	CDR11.5f	1.0	0.5	multidrug resistance protein, 5-pr	orf19.918
CA0616	PHO11	1.4	0.5	Secreted acid phosphatase	orf19.2619
CA0644	IPF17131	0.9	0.5	unknown function	orf19.69
CA0860	IPF17272.3f	0.9	0.5	unknown function	orf19.3522
CA0867	IPF14773.3f	1.0	0.5	unknown function, exon 2	orf19.3586
CA0882	PHR3	1.3	0.5	surface glycoprotein (by homolog	orf19.377
CA0914	SRA1	1.3	0.5	cAMP dependent protein kinase,	orf19.2014
CA1009	IPF14084	1.0	0.5	unknown function	orf19.6255
CA1020	IPF4824	0.8	0.5	unknown function	orf19.3355
CA1111	RIB3.5f	0.9	0.5	3,4-dihydroxy-2-butanone 4-phos	orf19.5228
CA1164	ARP8	0.8	0.5	actin-related protein (by homolog	orf19.3359
CA1193	MSY1	0.9	0.5	tyrosyl-tRNA synthetase 8by hom	orf19.109
CA1196	IPF10394	0.8	0.5	unknown function	orf19.3364
CA1199	IPF10404	0.7	0.5	unknown function	orf19.3369
CA1333	MRF1	1.1	0.5	mitochondrial respiratory function	orf19.1149
CA1400	IPF8067	0.9	0.5	unknown function	orf19.3697
CA1426	ALS9.3f	1.6	0.5	agglutinin-like protein, 3-prime en	orf19.5742
CA1458	IPF6342	1.5	0.5	unknown function	orf19.1106
CA1477	YME1	0.8	0.5	family of ATPases	orf19.1252
CA1621	IFF6	0.8	0.5	unknown function	orf19.4072
CA1625	IPF6156	1.1	0.5	similar to C.elegans LIM homeob	orf19.1034
CA1787	IFH3	1.2	0.5	Dioxygenase (by homology)	orf19.1167
CA1897	IPF12002	0.9	0.5	unknown function	orf19.2757
CA1943	IPF11759	0.8	0.5		orf19.4737
CA1951	HEM14	0.8	0.5	Mitochondrial protoporphyrinoge	orf19.4747
CA2032	IPF13112	0.7	0.5	unknown function	orf19.3466
CA2151	ERC2	0.9	0.5	ethionine resistance protein (by h	orf19.6023
CA2223	PLB5	0.9	0.5	putative phospholipase B precurs	orf19.5102
CA2231	IPF10934	0.9	0.5	similar to Saccharomyces cerevis	orf19.3926
CA2314	IPF14545	1.1	0.5	unknown function	orf19.1381
CA2411	IFI3.3f	0.9	0.5	Unknown function, 3-prime end	orf19.4482
CA2475	PGA15	1.2	0.5	unknown function	orf19.2878
CA2496	IPF3468	0.9	0.5	unknown function	orf19.4055
CA2527	IPF4331	1.1	0.5	unknown function	orf19.5527
CA2856	IPF7062	0.9	0.5	unknown function	orf19.1066
CA2938	IPF8321	0.8	0.5	similar to Saccharomyces cerevis	orf19.3325
CA3014	IPF11915	0.7	0.5	similar to Saccharomyces cerevis	orf19.6225
CA3172	IPF7676	0.8	0.5	unknown function	orf19.1400
CA3218	IPF10005	1.0	0.5	unknown function	orf19.6196
CA3221	IPF10000	0.9	0.5	unknown function, 5-prime end	orf19.6193
CA3359	IPF9566	0.7	0.5	unknown function	orf19.1939
CA3377	CLF1	0.8	0.5	pre-mRNA splicing factor (by hor	orf19.332

CA3381	NPR2	1.0	0.5	nitrogen permease regulator (by	orf19.328
CA3503	IPF15890	1.3	0.5	cytoskeletal binding protein (by h	orf19.6349
CA3578	IPF11105	1.1	0.5	probable quinone oxidoreductase	orf19.2262
CA3590	ARE2	1.6	0.5	acyl-CoA sterol acyltransferase-l	orf19.2248
CA3656	IPF7456	0.9	0.5	unknown function	orf19.2047
CA3657	IPF7459	0.9	0.5	unknown function	orf19.2049
CA3706	PSA2	1.1	0.5	mannose-1-phosphate guanyltra	orf19.4943
CA3756	IPF8884	0.8	0.5	unknown function	orf19.3422
CA3931	IPF12719	3.0	0.5	unknown function	orf19.5103
CA3966	IPF9376	2.2	0.5	unknown function	orf19.1427
CA3985	POB3	0.9	0.5	Binds DNA polymerase delta (by	orf19.1560
CA4040	GAL1	0.7	0.5	galactokinase	orf19.3670
CA4084	TPS1	1.7	0.5	TREHALOSE-6-PHOSPHATE S	orf19.6640
CA4175	IFX1	1.0	0.5	unknown function	orf19.2958
CA4245	PGA21	0.9	0.5	unknown function	orf19.532
CA4246	PGA20	1.0	0.5	unknown function	orf19.535
CA4276	HEX1	0.7	0.5	ß-N-acetylglucosaminidase	orf19.6673
CA4284	DPH52	0.8	0.5	Diphthamide methyltransferase,	orf19.1124.2
CA4437	IPF13867	2.4	0.5	unknown function	orf19.5158
CA4488	MEK1	1.1	0.5	serine/threonine protein kinase, 3	orf19.1874
CA4576	IPF9425	1.1	0.5	unknown function, 3-prime end	orf19.6888
CA4608	CCH1	0.8	0.5	Calcium channel protein (by hom	orf19.3298
CA4619	IPF5756	1.0	0.5	unknown function	orf19.3310
CA4674	HEM2	1.0	0.5	Porphobilinogen synthase (by ho	orf19.898
CA4823	ERC3	0.8	0.5	ethionine resistance protein (by h	orf19.2073
CA4836	PGA3	0.8	0.5	Similar to superoxide dismutase	orf19.2060
CA5144	IPF1019	1.2	0.5	unknown function	orf19.4581
CA5157	IPF993	0.7	0.5	unknown function	orf19.4595
CA5168	IPF6032	0.7	0.5	unknown function	orf19.4607
CA5206	GPH1	1.2	0.5	Glycogen phosphorylase (by hon	orf19.7021
CA5353	IML2	0.9	0.5	unknown function	orf19.7229
CA5555	SUC1	0.7	0.5	Putative zinc finger protein Suc1	orf19.7319
CA5559	CBP1	0.9	0.5	Corticosteroid binding protein	orf19.7323
CA5612	KAP114	0.7	0.5	putative RAN-binding protein/imp	orf19.7086
CA5773	DOT5	1.3	0.5	Derepression of telomeric silenci	orf19.5417
CA5825	IPF2489	1.0	0.5	unknown function	orf19.7480
CA5906	IPF5949	0.9	0.5	unknown function	orf19.6800
CA5908	IPF5960	0.7	0.5	unknown function	orf19.6797
CA5915	IPF10735	1.0	0.5	similar to Saccharomyces cerevis	orf19.6790
CA5973	IPF931	1.5	0.5	unknown function	orf19.7567
CA6060	IPF8301	0.9	0.5	unknown function	orf19.6008
CA0009	IPF11382	1.0	0.6	unknown function	orf19.634
CA0038	CYC1	0.9	0.6	cytochrome-c isoform 1	orf19.1770
CA0085	IPF16201	0.8	0.6	unknown function	orf19.693
CA0103	IPF17515	0.6	0.6	unknown function	orf19.48
CA0109	IPF13723	1.1	0.6	unknown function	orf19.260
CA0112	IPF17358	1.1	0.6	unknown function	orf19.5754
CA0113	AUT2	1.4	0.6	anchor protein mediateing attach	orf19.2401
CA0217	MNN41	1.0	0.6	regulates the mannosylphosphor	orf19.849
CA0227	PHO23	1.3	0.6	Involved in transcriptional regulat	orf19.1759
CA0229	IPF8147	1.3	0.6	unknown function	orf19.6200
CA0328	IPF15217	0.5	0.6	WD-repeat protein, 3-prime end (	orf19.3778

CA0354	YAK1	0.8	0.6	serine/threonine protein kinase, 5	orf19.147
CA0400	IPF7423	0.8	0.6	similar to Saccharomyces cerevis	orf19.6222
CA0439	SCRC1	1.0	0.6	unknown function	orf19.5569
CA0440		1.0	0.6	unknown function	orf19.5569
CA0484	IPF19622	1.0	0.6	unknown function	orf19.115
CA0543	IPF3255	0.8	0.6	similar to Saccharomyces cerevis	orf19.2867
CA0548	PHO87	1.1	0.6	Member of the phosphate perme	orf19.2454
CA0556	IPF15920	1.2	0.6	zinc-finger containing protein (by	orf19.4972
CA0576	NPR1	1.0	0.6	nitrogen permease reactivator pr	orf19.6232
CA0651	ALK8	0.8	0.6	n-alkane inducible cytochrome P	orf19.10
CA0680	IPF14511	0.8	0.6	unknown function, 5-prime end	orf19.4699
CA0779	IPF20065	0.8	0.6	similar to Saccharomyces cerevis	orf19.1135
CA0851	IPF16670	0.9	0.6	unknown function	orf19.1746
CA0890	IPF11766	1.0	0.6	unknown function	orf19.4734
CA0899	IPF4905	1.1	0.6	unknown function	orf19.411
CA0921	IPF12992	0.7	0.6	unknown function	orf19.1043
CA1035	IFI2.3f	1.0	0.6	unknown function, 3-prime end	orf19.1037
CA1071	IPF16624	0.7	0.6	unknown function	orf19.2534
CA1123	IPF19723	1.3	0.6	similar to Saccharomyces cerevis	orf19.6261
CA1165	IPF19902	0.9	0.6	unknown function	orf19.3360
CA1270	IPF13504	0.9	0.6	unknown function	orf19.3156
CA1388	IPF16514	0.7	0.6	unknown function	orf19.921
CA1408	IPF10513	0.8	0.6	unknown function	orf19.2883
CA1434	SNI2	1.0	0.6	similar to Saccharomyces cerevis	orf19.1203
CA1487	GPT1	0.8	0.6	polyamine transporter	orf19.4063
CA1514	IPF7539	1.0	0.6	unknown function	orf19.4886
CA1573	IPF15087	0.8	0.6	unknown function	orf19.1653
CA1594	IPF11051	0.9	0.6	unknown function	orf19.4321
CA1611	SPO72	0.9	0.6	required for sporulation (by homo	orf19.4119
CA1668	IPF11396	0.9	0.6	unknown function	orf19.2853
CA1688	COX15	1.2	0.6	cytochrome oxidase assembly fa	orf19.3656
CA1689	IPF13030	1.0	0.6	unknown function	orf19.3655
CA1692	IPF17055	1.1	0.6	unknown function, 3-prime end	orf19.1297
CA1822	IPF9525	2.2	0.6	unknown function	orf19.4268
CA1827	IPF9520	1.0	0.6	unknown function	orf19.4264
CA1834	PFK1	2.2	0.6	6-phosphofructokinase, alpha su	orf19.3967
CA1835	CRH12	1.1	0.6	Cell wall protein (by homology)	orf19.3966
CA1926	IPF13407	0.9	0.6	Unknown function	orf19.753
CA1947	IPF10637	1.3	0.6	starvation protein -like (by homol	orf19.4742
CA1954	IPF3897	1.0	0.6	unknown function, 5-prime end	orf19.764
CA1955	IPF3897	1.0	0.6	unknown function, 3-prime end	orf19.764
CA1975	PLB1	1.6	0.6	phospholipase B	orf19.689
CA2025	IPF9618	1.1	0.6	unknown function	orf19.4911
CA2076	ERO1	1.4	0.6	Required for protein disulfide bor	orf19.4871
CA2093	IPF3406	0.8	0.6	unknown function	orf19.841
CA2115	IPF8030	1.2	0.6	unknown function	orf19.1331
CA2143	IPF12803	0.9	0.6	unknown function	orf19.2512
CA2214	IPF9939	0.9	0.6	similar to Saccharomyces cerevis	orf19.4752
CA2237	IPF10032	0.8	0.6	unknown function, 3-prime end	orf19.3916
CA2238	IPF10032	0.8	0.6	unknown function, 5-prime end	orf19.3916
CA2257	SHR5	1.3	0.6	RAS suppressor (by homology)	orf19.1955
CA2262	IPF6857	1.6	0.6	putative transcriptional regulator	orf19.1757

CA2289	RBF1	1.3	0.6	RPG-BOX-BINDING FACTOR, 3	orf19.5558
CA2300	PMA1	0.7	0.6	plasma membrane H+-transporti	orf19.5383
CA2317	IPF13586	3.1	0.6	unknown function	orf19.5479
CA2356	IPF12282	0.8	0.6	unknown function	orf19.4893
CA2363	IPF10590	1.0	0.6	unknown function	orf19.1285
CA2380	IPF4072	0.8	0.6	unknown function	orf19.1860.1
CA2445	IPF13885	1.1	0.6	unknown function	orf19.5503
CA2450	IPF2589	0.9	0.6	unknown function	orf19.5508
CA2476	IFF5	1.0	0.6	unknown function	orf19.2879
CA2528	MOB1	0.9	0.6	required for completion of mitosis	orf19.5528
CA2611	IPF5453	1.6	0.6	unknown function	orf19.5692
CA2618	SNG2	1.0	0.6	drug transporter (by homology)	orf19.2812
CA2622	PER3	0.8	0.6	peroxisomal import protein, exon	orf19.2805
CA2623	PER3	0.8	0.6	peroxisomal import protein, exon	orf19.2805
CA2650	STV1	1.1	0.6	H+-ATPase V0 domain (by homo	orf19.1190
CA2721	IPF4799	1.1	0.6	unknown Function	orf19.3342
CA2782	IPF3806	1.1	0.6	unknown function	orf19.285
CA2868	IPF20118	1.3	0.6	unknown function	orf19.2846
CA2896	IPF14981	0.9	0.6	unknown function	orf19.3483
CA2948	GDS1	0.8	0.6	nam9-1 suppressor (by homolog	orf19.1963
CA2949	IPF14506	0.8	0.6	unknown function	orf19.1961
CA3036	IPF13443	0.9	0.6	unknown function	orf19.211
CA3062	IPF2968	0.8	0.6	unknown function	orf19.4286
CA3105	IPF12083	0.9	0.6	unknown function	orf19.6553
CA3124	IPF5353	0.8	0.6	unknown function, 3-prime end	orf19.3003
CA3159	PLC3	0.8	0.6	phosphatidylinositol phospholipa	orf19.1586
CA3220	IPF10001	1.1	0.6	unknown function	orf19.6194
CA3237	SPB4	0.8	0.6	ATP-dependent RNA helicase of	orf19.6298
CA3240	IPF10559	0.7	0.6	myosin-like protein (by homology	orf19.4683
CA3253	PGA23	0.8	0.6	unknown Function	orf19.3740
CA3259	IPF7970	1.4	0.6	unknown function	orf19.2691
CA3329	IPF12162	0.9	0.6	Unknown function	orf19.2670
CA3337	IPF13583	1.1	0.6	unknown function	orf19.2334
CA3346	IPF13356	1.1	0.6	molybdopterin biosynthesis (by h	orf19.2324
CA3358	IPF19984	0.8	0.6	similar to Saccharomyces cerevis	orf19.1940
CA3436	NRD1	1.2	0.6	Involved in regulation of nuclear	orf19.581
CA3489	IPF3195	0.9	0.6	unknown function	orf19.3567
CA3525	DJP1	0.9	0.6	DnaJ-like protein involved in pero	orf19.991
CA3526	MRPS5	0.8	0.6	Probable ribosomal protein S5, m	orf19.989
CA3529	IPF9929	1.4	0.6	unknown function	orf19.985
CA3638	IPF9252	0.7	0.6	unknown function	orf19.5134
CA3658	TGL1	0.7	0.6	Triacylglycerol lipase (by homolo	orf19.2050
CA3667	IPF16323	0.7	0.6	similar to Saccharomyces cerevis	orf19.6460
CA3737	BLM3	0.9	0.6	bleomycin resistance (by homolo	orf19.2182
CA3768	IPF13467	1.2	0.6	Putative peroxisomal 2,4-dienoyl	orf19.2899
CA3818	IPF6695	1.0	0.6	unknown function	orf19.5773
CA3971	IPF6498	1.0	0.6	unknown function	orf19.1543
CA4016	GFA1	0.8	0.6	glutamine:fructose-6-phosphate a	orf19.1618
CA4034	HSP31	0.7	0.6	heat shock protein (by homology	orf19.3664
CA4050	IPF20007	1.0	0.6	unknown function, exon 2	orf19.425
CA4155	RNR21	0.8	0.6	ribonucleoside-diphosphate redu	orf19.5801
CA4193	IPF7535	1.4	0.6	unknown function	orf19.4534

CA4198	IPF8055	0.7	0.6	unknown function	orf19.4539
CA4205	IFA10	1.1	0.6	unknown function	orf19.4549
CA4215	SSY1	0.8	0.6	Regulator of transporters (by hor	orf19.814
CA4255	FUM11	0.8	0.6	fumarate hydratase	orf19.543
CA4261	PDX3	1.3	0.6	pyridoxamine-phosphate oxidase	orf19.550
CA4304	IPF6649	0.9	0.6	unknown function	orf19.6704
CA4324	IPF2095	0.8	0.6	unknown function	orf19.4095
CA4346	IPF3634	0.8	0.6	unknown function	orf19.6720
CA4381	PGA10	1.5	0.6	unknown function	orf19.5674
CA4436	IPF13868	0.9	0.6	unknown function	orf19.5159
CA4479	URH1	0.8	0.6	Uridine ribohydrolase (by homolo	orf19.1888
CA4501	QDR1	1.0	0.6	putative antibiotic resistance prot	orf19.508
CA4560	IPF8038	1.0	0.6	unknown function	orf19.6871
CA4587	IPF2223	0.9	0.6	unknown function	orf19.6905
CA4615	IPF7955	0.8	0.6	DNA binding protein (by homolog	orf19.3306
CA4618	IPF5757	1.0	0.6	unknown function	orf19.3309
CA4622	IPF5751	1.3	0.6	TRAPP subunit of 20 kDa involve	orf19.3314
CA4683	HSP78	13.5	0.6	heat shock protein of clpb family	orf19.882
CA4684	HSP78	13.5	0.6	heat shock protein of clpb family	orf19.882
CA4722	SAC1	0.9	0.6	integral membrane protein localiz	orf19.4865
CA4728	VPS41	0.8	0.6	required for the vacuolar assemb	orf19.4858
CA4729	VPS41	0.8	0.6	required for the vacuolar assemb	orf19.4858
CA4743	IPF4491	0.8	0.6	unknown function	orf19.4839
CA4763	IPF9645	1.0	0.6	similar to Saccharomyces cerevis	orf19.3995
CA4819	IPF1194	0.8	0.6	Similar to clathrin coat proteins	orf19.2078
CA4845	IPF6945	1.1	0.6	unknown function, 5-prime end	orf19.3813
CA4905	DLD1	0.8	0.6	D-lactate ferricytochrome C oxido	orf19.6043
CA4949	CDC34	1.5	0.6	Ubiquitin-conjugating enzyme (by	orf19.6529
CA4951	IPF13607	1.1	0.6	unknown function	orf19.6527
CA4987	IPF2045	1.2	0.6	unknown function	orf19.5262
CA5020	IPF7556	1.0	0.6	similar to Saccharomyces cerevis	orf19.6973
CA5029	IPF2997	3.2	0.6	unknown function	orf19.6983
CA5039	GAP2	1.6	0.6	general amino acid permease (by	orf19.6993
CA5100	IPF1372	0.8	0.6	unknown function	orf19.6440
CA5105	PEX19	0.8	0.6	Required for biogenesis of perox	orf19.6434
CA5112	PGA13	0.7	0.6	Similarity to mucin proteins (by h	orf19.6420
CA5130	IFH2	1.2	0.6	Dioxygenase (by homology)	orf19.6398
CA5131	IPF5124	1.5	0.6		orf19.6396
CA5132	GTS1	1.3	0.6	Transcription factor by homology	orf19.6393
CA5172	CPS2.5f	0.7	0.6	Carboxypeptidase YSCS precurs	orf19.4610
CA5176	IPF6045	1.0	0.6	unknown function	orf19.4614
CA5186	HRT2	0.8	0.6	Similar to ScHRT2 (by homology	orf19.4624
CA5234	IPF3014	0.9	0.6	weak similarity to S. cerevisiae D	orf19.5030
CA5271	IPF1777	1.0	0.6	similar to Saccharomyces cerevis	orf19.4991
CA5294	IPF1952	1.0	0.6	unknown function	orf19.7157
CA5358	IPF846	0.8	0.6	WD-repeat protein, beta-transduc	orf19.7235
CA5362	IPF836.3	0.7	0.6	regulation of G-protein function, 3	orf19.7239
CA5368	IPF5257	0.9	0.6	unknown function	orf19.7250
CA5442	IPF18105	1.4	0.6	unknown function, 3-prime end	orf19.3245
CA5443	IPF18105	1.4	0.6	unknown function, 3-prime end	orf19.3245
CA5505	TPS3	0.8	0.6	alpha,alpha-trehalose-phosphate	orf19.5346
CA5538	IPF2830	0.9	0.6	unknown function	orf19.7300

CA5543	IPF5988	0.8	0.6	unknown function	orf19.7305
CA5564	IPF1980	0.9	0.6	unknown function	orf19.7328
CA5565	QRI8	1.2	0.6	E2 ubiquitin-conjugation enzyme	orf19.7329
CA5577	IPF12537	1.0	0.6	unknown function	orf19.7344
CA5579	IPF5661	1.0	0.6	unknown function	orf19.7125
CA5705	IPF2645	1.3	0.6	unknown function	orf19.7405
CA5761	IFF11	1.0	0.6	unknown function	orf19.5399
CA5763	IFF10.5f	0.9	0.6	unknown function, 5-prime end	orf19.5404
CA5772	ESA1	1.0	0.6	Histone acetyltransferase (by hor	orf19.5416
CA5778	IPF1119	1.0	0.6	unknown function	orf19.5423
CA5804	IPF4160	1.1	0.6	unknown function	orf19.5459
CA5849	IPF404	0.9	0.6	unknown function, 5-prime end	orf19.7506
CA5890	FCR1	0.7	0.6	Zinc cluster transcription factor	orf19.6817
CA5936	IPF2795	0.9	0.6	unknown function	orf19.6763
CA5939	IPF3484	1.4	0.6	aldo/keto reductase (by homolog	orf19.6758
CA5940	IPF3485	1.3	0.6	aldo/keto reductase (by homolog	orf19.6757
CA5955	IPF3510	1.3	0.6	unknown function	orf19.6739
CA5985	IPF907	1.3	0.6	unknown function	orf19.7583
CA6034	APC11	1.1	0.6	subunit of the anaphase promotir	orf19.7644
CA6073	HRP1	1.1	0.6	Nuclear polyadenylated RNA-bin	orf19.5989
CA6091	UFD2	0.9	0.6	Ubiquitin fusion degradation prot	orf19.5965
CA6096	IPF56	3.2	0.6	similar to Saccharomyces cerevis	orf19.5961
CA6100	IPF66	1.3	0.6	unknown function	orf19.5956

8) Genes that were sensitive to doxycycline in wild type *C. albicans* cells

Mean fold change is shown from >3 independent experiments

UP-REGULATED

GENE			Reg	Function
CA4381	PGA10	orf19.5674	5.4	unknown function
CA3061	IPF2965	orf19.11763	4.9	unknown function
CA6057	IPF4959	orf19.7676	4.8	D-xylulose reductase (by homology)
CA3878	IPF7289	orf19.391	4.8	similar to Saccharomyces cerevisiae Upc2p RNA polymerase II transcription factor
CA2391	ADH5	orf19.2608	4.6	probable alcohol dehydrogenase (by homology)
CA3895	CDR4	orf19.5079	4.6	Multidrug resistance protein
CA5505	TPS3.3	orf19.5348	4.6	alpha,alpha-trehalose-phosphate synthase, regulatory subunit, 3-prime end (by homology)
CA0262	IPF20054	orf19.1407	4.4	unknown function
CA0386	IPF4065	orf19.1862	4.3	unknown function
CA0263	GLK1	orf19.1408	4.1	aldohexose specific glucokinase (by homology)
CA5467	GSY1	orf19.3278	4.0	UDP glucose--starch glucosyltransferase, glycogen synthase (by homology)
CA2938	IPF8321	orf19.3325	3.9	similar to Saccharomyces cerevisiae Glg2p self-glucosylating initiator of glycogen synthesis (by homology)
CA4516	IPF18207	orf19.489	3.8	unknown function
CA5322	PRB1	orf19.7196	3.7	Protease B, vacuolar (by homology)
CA3880	CAF16	orf19.388	3.7	ABC ATPase (by homology)
CA0273	IPF19066	orf19.1433	3.6	unknown function
CA2490	MUM2	orf19.4044	3.6	ubiquitin C-terminal hydrolase (by homology)
CA0824	GPD2	orf19.691	3.6	Glycerol 3-phosphate dehydrogenase (by homology)
CA2832	CRD1	orf19.4784	3.5	Cu-transporting P1-type ATPase
CA2216	IPF6235	orf19.5372	3.5	Candida albicans Tca2 retrotransposon
CA1528	ALS4.3F	orf19.4556	3.4	agglutinin-like protein, 3-prime end
CA0413	ALS12.3F	orf19.2122	3.4	agglutinin-like protein, 3-prime end
CA0706	IPF7715	orf19.8300	3.3	unknown function
CA4266	IPF2283	orf19.6660	3.3	unknown function

CA4570	IPF9550	orf19.6882	3.3	similar to Saccharomyces cerevisiae Osm1p osmotic growth protein
CA4492	RNR22	orf19.1868	3.2	ribonucleoside-diphosphate reductase (by homology)
CA4059	PRD1	orf19.8064	3.2	Proteinase (by homology)
CA4084	TPS1	orf19.6640	3.2	TREHALOSE-6-PHOSPHATE SYNTHASE
CA4745	URA1	orf19.4836	3.2	dihydroorotate dehydrogenase
CA2558	RBT5	orf19.5636	3.1	repressed by TUP1 protein 5
CA2697	IPF9167	orf19.2737	3.1	unknown function
CA5144	IPF1019	orf19.4581	3.1	unknown function
CA4852	SCS7	orf19.3822	3.0	Required for hydroxylation of ceramide (by homology)
CA3579	HSH49	orf19.2261	3.0	spliceosome-associated essential protein [Candida albicans]
CA0357	FCY22	orf19.333	2.9	purine-cytosine permease (by homology)
CA3656	IPF7456	orf19.2047	2.9	unknown function
CA2493	IPF7227	orf19.4048	2.9	putative fatty acid desaturase (by homology)
CA4127	IPF6629	orf19.2762	2.9	unknown function
CA2225	SUR2	orf19.5818	2.8	Hydroxylation of C-4 of the sphingoid moiety of ceramide by homology
CA1688	COX15	orf19.3656	2.8	cytochrome oxidase assembly factor (by homology)
CA4250	IPF4292	orf19.539	2.8	bleomycin Hydrolase
CA4220	IPF8762	orf19.822	2.8	unknown function
CA4780	IPF3352	orf19.4013	2.8	unknown function
CA0917	RAD16	orf19.2969	2.8	nucleotide excision repair protein (by homology)
CA1411	IPF11858	orf19.1277	2.8	unknown function
CA4926	IPF1404	orf19.6065	2.8	unknown function
CA5100	IPF1372	orf19.6440	2.7	unknown function
CA5824	NTH1	orf19.7479	2.7	Neutral trehalase
CA2263	GPD1	orf19.1756	2.7	Glycerol-3-phosphate dehydrogenase (by homology)
CA2214	IPF9939	orf19.4752	2.7	similar to Saccharomyces cerevisiae Msn4p transcriptional activator (by homology)
CA4827	SMF2	orf19.2069	2.7	Manganese transporter (by homology)
CA0892	PHO8.5	orf19.4736	2.7	repressible alkaline phosphatase, 5-prime end (by homology)
CA4081	IPF2523	orf19.6637	2.7	unknown function
CA2314	IPF14545	orf19.1381	2.7	unknown function
CA4823	ERC3	orf19.2073	2.6	ethionine resistance protein (by homology)

CA1957	IPF3887	orf19.768	2.6	similar to Saccharomyces cerevisiae Syg1p plasma membrane protein of the major facilitator superfamily (I
CA4951	IPF13607	orf19.6527	2.6	unknown function
CA5761	IFF11	orf19.5399	2.6	unknown function
CA1975	PLB1	orf19.689	2.6	phospholipase B
CA3257	IFC1	orf19.3746	2.6	Unknown Function
CA5788	RHR2	orf19.5437	2.5	DL-glycerol phosphatase
CA2280	DUR1,2	orf19.780	2.5	urea amidolyase (by homology)
CA4153	DNLI	orf19.5798	2.5	CANAL DNA LIGASE (POLYDEOXYRIBONUCLEOTIDE SYNTHASE [ATP])
CA3387	MUP3	orf19.7953	2.5	Very low affinity methionine permease
CA0270	PRB2	orf19.9783	2.5	Protease B, vacuolar (by homology)
CA3590	ARE2	orf19.2248	2.5	acyl-CoA sterol acyltransferase-like (by homology)
CA5076	IPF15301	orf19.3051	2.5	unknown function
CA3160	ZRT2	orf19.1585	2.5	zinc transport protein (by homology)
CA2714	IFF2	orf19.575	2.5	unknown function
CA0984	IPF7400	orf19.1802	2.5	unknown function
CA2302	IPF6518	orf19.1691	2.5	unknown function
CA5559	CBP1	orf19.7323	2.5	Corticosteroid binding protein
CA2560	PEX5	orf19.5640	2.4	peroxisomal targeting signal receptor
CA4874	IPF1674	orf19.6559	2.4	putative transcription initiation factor (by homology)
CA4587	IPF2223	orf19.6905	2.4	unknown function
CA5170	C12.EXO	orf19.4608	2.4	Pyruvate decarboxylase I, exon 2 (by homology)
CA1111	RIB3	orf19.12693	2.4	3,4-dihydroxy-2-butanone 4-phosphate synthase (by homology)
CA1951	HEM14	orf19.12209	2.4	Mitochondrial protoporphyrinogen oxidase (by homology)
CA3931	IPF12719	orf19.5103	2.4	unknown function
CA1834	PFK1	orf19.3967	2.4	6-phosphofructokinase, alpha subunit
CA4657	IPF3094	orf19.4444	2.4	4-nitrophenyl phosphatase (by homology)
CA0526	BUB3	orf19.2655	2.4	cell cycle arrest protein (by homology)
CA2075	IFE2	orf19.5288	2.4	Unknown function
CA1261	F9887.3E	orf19.1240	2.4	unknown function, 3-prime end
CA2574	ATH1	orf19.6214	2.4	acid trehalase, vacuolar
CA4970	IPF4696	orf19.5282	2.3	unknown Function

CA5940	IPF3485	orf19.6757	2.3	aldo/keto reductase (by homology)
CA1191	CAN2	orf19.111	2.3	amino acid permease (by homology)
CA6080	IPF21	orf19.5980	2.3	unknown function
CA1737	LYS12	orf19.2525	2.3	homo-isocitrate dehydrogenase (by homology)
CA3743	IPF19998	orf19.2175	2.3	unknown function
CA1426	ALS11.3F	orf19.13168	2.3	agglutinin-like protein, 3-prime end
CA1330	IPF11713	orf19.8744	2.3	unknown function
CA3336	IPF19983	orf19.2335	2.3	unknown function
CA5072	IPF3598	orf19.3047	2.3	similar to Saccharomyces cerevisiae Sip3p protein which interacts with Snf1p protein kinase (by homology)
CA0081	IPF11379	orf19.633	2.3	unknown function
CA1148	CAN1	orf19.97	2.3	amino acid permease (by homology)
CA5955	IPF3510	orf19.6739	2.3	unknown function
CA6135	CMK1	orf19.5911	2.3	Ca2+/calmodulin-dependent ser/thr protein kinase (by homology)
CA4915	IPF1427	orf19.6055	2.3	Similar to ubiquitination protein Bul1p (by homology)
CA5766	IPF1097	orf19.5408	2.2	serine/threonine protein kinase (by homology)
CA2650	STV1	orf19.1190	2.2	H+-ATPase V0 domain (by homology)
CA3637	IPF9255	orf19.5136	2.2	unknown function
CA0692	PGM2	orf19.10359	2.2	Phosphoglucomutase (by homology)
CA1625	IPF6156	orf19.1034	2.2	similar to C.elegans LIM homeobox protein
CA0684	IPF4450	orf19.12173	2.2	unknown function
CA4949	CDC34	orf19.6529	2.2	Ubiquitin-conjugating enzyme (by homology)
CA5704	CHA11	orf19.7404	2.2	L-serine/L-threonine deaminase (by homology)
CA1944	IPF10645	orf19.4738	2.2	unknown function
CA5337	APL1	orf19.7212	2.2	AP-2 complex subunit, beta2-adaptin (by homology)
CA1123	IPF19723	orf19.6261	2.2	similar to Saccharomyces cerevisiae Bph1p involved in acetic acid export
CA2738	STF2	orf19.2107.1	2.2	ATP synthase regulatory factor (by homology)
CA3335	IPF8682	orf19.2336	2.2	unknown function
CA3679	IPF20142	orf19.730	2.2	unknown function
CA0691	CIRT4A	orf19.10357	2.1	Transposase (by homology)
CA1874	TPK2	orf19.2277	2.1	cAMP-dependent protein kinase 2 (by homology)
CA1902	OPT1	orf19.2602	2.1	oligopeptide transporter

CA0609	CDR11.3F	orf19.919	2.1	multidrug resistance protein, 3-prime end (by homology)
CA3757	MAE1	orf19.3419	2.1	mitochondrial malic enzyme (by homology)
CA4215	SSY1	orf19.8434	2.1	Regulator of transporters (by homology)
CA2017	IPF3414	orf19.846	2.1	putative serine/threonine protein kinase
CA4021	EBP2	orf19.3442	2.1	NADPH dehydrogenase (by homology)
CA6011	CTM1	orf19.7612	2.1	cytochrome c methyltransferase (by homology)
CA2870	OPT2.53F	orf19.2847	2.1	Oligopeptide transporter, internal fragment (by homology)
CA0627	HSP12	orf19.3160	2.1	Heat shock protein (by homology)
CA1465	RIM11	orf19.791	2.1	Ser/thr protein kinase (by homology)
CA4501	QDR1	orf19.8138	2.1	putative antibiotic resistance proteins (by homology)
CA5489	MDH12	orf19.5323	2.1	mitochondrial malate dehydrogenase (by homology)
CA1333	MRF1	orf19.8742	2.1	mitochondrial respiratory function protein (by homology)
CA4199	UBC8	orf19.4540	2.1	ubiquitin-conjugating enzyme (by homology)
CA4293	IFA8	orf19.6690	2.1	Unknown function
CA4748	MLS1	orf19.4833	2.1	malate synthase
CA3329	IPF12162	orf19.2670	2.1	Unknown function
CA0748	TFS1	orf19.1974	2.1	cdc25-dependent nutrient- and ammonia-response cell-cycle regulator (by homology)
CA4394	IPF12297	orf19.3117	2.1	mycelial surface antigen (by homology)
CA3870	YPK1	orf19.399	2.1	ser/thr-specific protein kinase (by homology)
CA5135	HSP104	orf19.13747	2.1	Heat shock protein (by homology)
CA0188	IPF15442	orf19.9467	2.0	unknown function
CA0109	IPF13723	orf19.7892	2.0	unknown function
CA5773	DOT5	orf19.5417	2.0	Derepression of telomeric silencing (by homology)
CA3745	MAF1	orf19.2173	2.0	nuclear protein by homology
CA4700	SAP9	orf19.6928	2.0	aspartyl proteinase 9 (by homology)
CA1592	IPF14662	orf19.4317	2.0	D-xylose reductase (by homology)
CA0214	SCT11	orf19.1289	2.0	Suppresses a choline-transport mutant
CA5728	IPF2471	orf19.7437	2.0	maltose acetyltransferase
CA3724	IPF6181	orf19.4174	2.0	similar to Saccharomyces cerevisiae Fun 26p nucleoside transporter (by homology)
CA2291	IPF9740	orf19.744	2.0	oligo-1,4 -1,4-glucantransferase / amylo-1,6-glucosidase (by homology)
CA4206	IPF9079	orf19.4550	2.0	Membrane transporter (by homology)

CA5866	IPF361	orf19.7527	2.0	unknown function
CA2076	ERO1	orf19.4871	2.0	Required for protein disulfide bond formation in the ER (by homology)
CA1373	AGP1	orf19.8784	2.0	asparagine and glutamine permease (by homology)
CA0232	IFA1	orf19.156	2.0	Unknown function

DOWN-REGULATED

GENE			Reg	Function
CA0472	STL1	orf19.13176	0.1	sugar transporter (by homology)
CA1945	MSS116	orf19.4739	0.3	RNA helicase of the DEAD box family (by homology)
CA1758	PF14559.3	orf19.5126	0.3	unknown function, 3-prime end
CA3909	CIT1	orf19.4393	0.3	Citrate synthase, exon 2
CA4894	IPF1617	orf19.6586	0.3	unknown function
CA0204	IKI3	orf19.1222	0.3	killer toxin insensitive protein 3 (by homology)
CA0089	MRPL3	orf19.5064	0.3	ribosomal protein of the large subunit, mitochondrial (by homology)
CA5621	FRE7	orf19.7077	0.3	Ferric reductase transmembrane component (by homology)
CA5063	IPF8493	orf19.3034	0.3	putative member of nontransporter group of ATP-binding cassette (ABC) superfamily (by homology)
CA1041	BMS1	orf19.2504	0.3	probable membrane protein involved in bud site selection (by homology)
CA0929	GUT1	orf19.558	0.3	Glycerol kinase (by homology)
CA6072	DBP10	orf19.5991	0.3	Putative ATP-dependent RNA helicase (by homology)
CA2930	IPF7840	orf19.4204	0.3	similar to Saccharomyces cerevisiae Pet123p ribosomal protein, mitochondrial (by homology)
CA1640	PF18641.3	orf19.1732	0.3	unknown function, exon 2
CA2898	IPF15646	orf19.3481	0.3	putative ATP-dependent RNA helicase (by homology)
CA1766	NOP4	orf19.5198	0.3	Nucleolar protein
CA2712	SPE2	orf19.8199	0.3	by homology to S. cerevisiae adenosylmethionine decarboxylase precurser
CA1088	IPF13319	orf19.740	0.3	unknown function
CA1666	SRP40	orf19.2859	0.3	RNA I and II supressor (by homology)
CA1500	IPF13221	orf19.1697	0.4	unknown function
CA4713	CCC1	orf19.6948	0.4	Transmembrane Ca2+ transporter (by homology)

CA0712	IPF13717	orf19.2167	0.4	unknown function
CA4067	IPF5924	orf19.445	0.4	unknown function
CA0715	DBP9	orf19.3393	0.4	dead box helicase
CA3927	DIP2	orf19.5106	0.4	beta transducin
CA2205	SEO2	orf19.8319	0.4	suppressor of sulfoxyde ethionine resistance
CA1274	SAS10	orf19.2717	0.4	Involved in silencing (by homology)
CA2034	SGD1	orf19.4363	0.4	Involved in HOG pathway, 3-prime end (by homology)
CA6038	LTV1	orf19.7650	0.4	low-temperature viability protein (by homology)
CA5668	IPF1261	orf19.7370	0.4	unknown function
CA4960	IPF3912	orf19.6514	0.4	unknown function
CA1803	IPF16748	orf19.2917	0.4	unknown function
CA0098	IPF16479	orf19.2319	0.4	unknown function
CA4892	PRT1	orf19.6584	0.4	Translation initiation factor eIF3 (by homology)
CA6061	IPF8302	orf19.6007	0.4	unknown function
CA1002	ROK1.3f	orf19.3756	0.4	RNA helicase, 3-prime end
CA5682	NOG1	orf19.7384	0.4	Nucleolar G-protein (by homology)
CA0580	[illegible]3184.EX[illegible]	orf19.3559	0.4	unknown function, exon 2
CA0462	MRPS28	orf19.2520	0.4	ribosomal protein (by homology)
CA5719	IPF2441	orf19.7424	0.4	unknown function
CA2772	IPF20112	orf19.2688	0.4	unknown function
CA2099	IPF11484	orf19.2386	0.4	unknown function
CA1938	NMD5	orf19.4188	0.4	putative Nam7p/Upf1p-interacting protein (by homolgy)
CA4222	GCD7	orf19.825	0.4	translation initiation factor eIF2b subunit (by homology)
CA2449	ENP1	orf19.5507	0.4	Essential nuclear protein (by homology)
CA2881	RAD4	orf19.5850	0.4	Excision repair protein (by homology)
CA2847	NOG2	orf19.5732	0.4	unknown function, exon 1
CA4470	IPF9717	orf19.1902	0.4	unknown function
CA1709	HOL2	orf19.4889	0.4	Multidrug-resistance protein subfamily 1 (by homology)
CA2368	RPA190	orf19.1839	0.4	DNA-directed RNA polymerase I (by homology)

CA5051	IPF3709	orf19.3015	0.4	unknown function
CA5510	IPF741	orf19.5356	0.4	unknown function
CA1822	IPF9525	orf19.4268	0.4	unknown function
CA1443	IPF4776	orf19.4492	0.4	unknown Function
CA4289	IPF6665	orf19.6686	0.4	unknown function
CA0443	SPB1	orf19.7727	0.4	Putative methyltransferase by homology
CA5474	F16944.3e	orf19.3287	0.4	unknown function, 3-prime end
CA1397	TOM72	orf19.3700	0.4	mitochondrial import receptor (by homology)
CA4465	IPF9829	orf19.6862	0.4	unknown function
CA3168	PRS3	orf19.1575	0.4	ribose-phosphate pyrophosphokinase
CA3308	PET127.5	orf19.9845	0.4	component of mitochondrial translation (by homology)
CA2992	RRS1	orf19.6014	0.4	Regulator for ribosome synthesis (by homology)
CA0201	IPF17419	orf19.9108	0.4	Unknown function
CA3828	SNQ2	orf19.5759	0.4	multidrug resistance protein (by homology)
CA5685	HPA1	orf19.7387	0.4	RNA polymerase II-associated Histone acetyltransferase (by homology)
CA6056	IPF4955	orf19.7675	0.5	similar to Saccharomyces cerevisiae Mrpl25p ribosomal protein YmL25, mitochondrial (by homology)
CA3229	FUN30	orf19.6291	0.5	helicases of the Snf2/Rad54 family(by homology)
CA2399	MAK5	orf19.3540	0.5	ATP-dependent RNA helicase (by homology)
CA2126	IPF4896	orf19.417	0.5	unknown function
CA4630	IPF1537	orf19.336	0.5	putative adrenodoxin and ferrodoxin (by homolgy)
CA6021	BFR2	orf19.7624	0.5	involved in protein transport steps at the Brefeldin A block (by homology)
CA4211	IPF5052	orf19.809	0.5	RNA-binding protein (by homology)
CA5696	F3309.3e	orf19.7398	0.5	unknown function, 3-prime end
CA2515	IPF19759	orf19.5510	0.5	Unknown Function
CA3165	FMI1	orf19.1578	0.5	processing of pre-ribosomal RNA
CA2044	IPF10668	orf19.10447	0.5	unknown function
CA1914	IPF16564	orf19.2438	0.5	putative mitochondrial ribosomal protein S12
CA0923	IPF12987	orf19.1047	0.5	unknown function
CA3993	IPF8275	orf19.9142	0.5	unknown function

CA5652	IPF19815	orf19.7354	0.5	longevity-assurance protein (by homology)
CA1000	IPF6011	orf19.9061	0.5	unknown function
CA4350	FUM12.53	orf19.6724	0.5	Fumarate hydratase, internal fragment (by homology)
CA0235	HIS3	orf19.7813	0.5	imidazole glycerol phosphate dehydratase
CA1293	HCA4	orf19.2712	0.5	Can suppress the U14 snoRNA rRNA processing function
CA0813	MPP10	orf19.1915	0.5	component of the U3 small nucleolar ribonucleoprotein (by homology)
CA4708	MEU1	orf19.6938	0.5	regulator of ADH2 expression (by homology)
CA6134	MAK21	orf19.5912	0.5	Ribosome biogenesis protein (by homology)
CA4810	IPF1164	orf19.2091	0.5	Subunit NUHM of NADH:Ubiquinone Oxidoreductase (by homology)
CA3707	TYE7	orf19.4941	0.5	Basic helix-loop-helix transcription factor by homology
CA1779	IPF14510	orf19.1826	0.5	unknown function
CA1747	IPF10884	orf19.5049	0.5	unknown function
CA2631	IPF14389	orf19.4758	0.5	ubiquinone oxidoreductase subunit NUIM (by homology)
CA1226	IPF15660	orf19.4159	0.5	putative mitochondrial carrier (by homology)
CA0027	RCL1	orf19.1886	0.5	RNA 3 -terminal phosphate cyclase (by homology)
CA5116	TPM2	orf19.6414.3	0.5	Tropomyosin, 3-prime end
CA5509	IPF743	orf19.5353	0.5	unknown function
CA0986	IPF4814	orf19.3350	0.5	similar to Saccharomyces cerevisiae Mrp20p ribosomal protein of the large subunit, mitochondrial (by homo
CA2035	SGD1	orf19.11841	0.5	Involved in HOG pathway, 5-prime end
CA5507	TIF11	orf19.5351	0.5	translation initiation factor eIF1a (by homology)
CA3726	IPF6175	orf19.4176	0.5	unknown function
CA1496	CTR1	orf19.3646	0.5	copper transport protein
CA0969	IPF9132	orf19.12987	0.5	unknown function
CA3094	IPF11548	orf19.11335	0.5	serine/threonine protein kinase (by homology)
CA1940	IPF7999	orf19.4190	0.5	unknown function
CA0579	F3184.EXO	orf19.3560	0.5	unknown function, exon 1
CA1110	DIS3	orf19.5229	0.5	3 --5 exoribonuclease required for 3 end formation of 5.8S rRNA (by homology)
CA4787	IPF3364	orf19.4021	0.5	Unknown function
CA1552	PUT1	orf19.4274	0.5	proline oxidase (by homology)
CA4033	IPF19795	orf19.3431	0.5	similar to Saccharomyces cerevisiae Mip1p DNA-directed DNA polymerase gamma catalytic subunit, mitoc

CA4099	IPF3986	orf19.665	0.5	unknown function
CA1416	BP2.EXON	orf19.171	0.5	ATP-dependent RNA helicase of DEAD box family, exon 1 (by homology)
CA2708	RPS6A	orf19.4660	0.5	ribosomal protein S6 (by homology)
CA2256	IPF14757	orf19.1956	0.5	unknown function
CA4502	IPF10391	orf19.8136	0.5	Similar to dnaJ proteins
CA4044	GAL7	orf19.3675	0.5	UDP-glucose-hexose-1-phosphate uridylyltransferase (by homology)
CA0641	IPF15977	orf19.4698	0.5	unknown function
CA1532	IPF9507.3	orf19.8806	0.5	unknown function
CA4718	IPF8661	orf19.6955	0.5	unknown function
CA1438	NOP58	orf19.1199	0.5	nucleolar protein required for pre-18S rRNA processing
CA0667	TIF5	orf19.11737	0.5	Translation initiation factor eIF5 (by homology)
CA0778	IPF12884	orf19.4779	0.5	unknown function
CA1999	IPF19513	orf19.13830	0.5	unknown function
CA5151	IPF1009	orf19.4590	0.5	Weak similarity to S. cerevisiae RFX1
CA4284	DPH52	orf19.6682	0.5	Diphthamide methyltransferase, 3-prime end (by homology)
CA4039	SKS1	orf19.3669	0.5	serine/threonine kinase by homology
CA1414	CHO2	orf19.169	0.5	phosphatidylethanolamine N-methyltransferase (by homology)
CA2304	PRP43	orf19.1687	0.5	RNA-dependent ATPase (by homology)
CA4097	GIN4	orf19.663	0.5	ser/thr protein kinase (by homology)
CA2593	RRP6	orf19.58	0.5	involved in 5.8S rRNA processing (by homology)
CA1770	IPF12457	orf19.2185	0.5	unknown function
CA5807	RPS24	orf19.5466	0.5	ribosomal protein S24.e
CA2255	CYC3	orf19.1957	0.5	cytochrome C heme lyase
CA5497	IPF776	orf19.5338	0.5	transcriptional activator (by homology)
CA1557	IPF15677	orf19.1710	0.5	probable NADH-ubiquinone oxidoreductase (by homology)
CA2154	ERG2	orf19.6026	0.5	C-8 sterol isomerase
CA5681	MNN9	orf19.7383	0.5	Required for complex N-glycosylation
CA1560	MET18	orf19.1706	0.5	Involved in NER repair and RNA polymerase II transcription (by homology)
CA5644	IPF447	orf19.7050	0.5	unknown function
CA4339	IPF3618	orf19.6710	0.5	Unknown function
CA5911	IPF5965	orf19.6794	0.5	NADH-ubiquinone oxidoreductase (by homology)

CA4092	DBP8	orf19.6652	0.5	DEAD box protein ATP-dependent RNA helicase (by homology)
CA5197	IPF2342	orf19.7011	0.5	unknown function
CA3130	TIM23	orf19.1361	0.5	mitochondrial inner membrane import translocase subunit (by homology)
CA1047	IPF11615	orf19.9215	0.5	RNA-binding proteins (by homology)
CA5466	PWP2	orf19.3276	0.5	periodic tryptophan protein (by homology)
CA3977	IPF20153	orf19.1549	0.5	unknown function
CA5292	IPF1948	orf19.7154	0.5	unknown function
CA1559	IPF16533	orf19.1708	0.5	unknown function
CA0570	SVL3	orf19.8732	0.5	Involved in vacuole function (by homology)
CA4296	IPF2605	orf19.6693	0.5	unknown function
CA2056	URA5	orf19.2555	0.5	Orotate phosphoribosyltransferase (by homology)
CA1702	HEL1	orf19.702	0.5	DNA helicase I (by homology)
CA6029	DRS1	orf19.7635	0.5	ATP dependent RNA helicase (by homology)
CA2670	MAK16	orf19.5500	0.5	nuclear viral propagation protein (by homology)
CA2326	NUBM	orf19.11971	0.5	nucleotide-binding respiratory complex I subunit (by homology)
CA3787	HPT1	orf19.5832	0.5	hypoxanthine guanine phosphoribosy transferase(by homology)
CA5665	UBP1	orf19.7367	0.5	Ubiquitin-specific protease (by homology)
CA1277	IPF10837	orf19.3630	0.5	unknown function
CA1757	PF14559.5	orf19.5128	0.5	unknown function, 5-prime end
CA5133	IPF5129	orf19.6392	0.5	unknown function
CA2986	CDC5	orf19.6010	0.5	Cell-cycle protein kinase (by homology)
CA2869	RPC82	orf19.2847	0.5	DNA-directed RNA polymerase III, 82 KD subunit (by homology)
CA0298	PPT1	orf19.1673	0.5	Protein ser/thr phosphatase
CA5718	LCP5	orf19.7422	0.5	Ngg1p interacting protein (by homology)
CA5243	IPF3040	orf19.5020	0.5	unknown function
CA2377	MRPL7	orf19.2214	0.5	Ribosomal protein of the large subunit, mitochondrial (by homology)
CA5419	PRS1	orf19.969	0.5	Ribose-phosphate pyrophosphokinase
CA4226	IPF8752	orf19.828	0.5	similar to Saccharomyces cerevisiae Mrpl24p ribosomal protein of the large subunit, mitochondrial (by hom
CA3990	IPF14782	orf19.1566	0.5	beta-transducin (by homology)
CA1385	IPF8424	orf19.925	0.5	unknown function
CA3446	IPF7324	orf19.2977	0.5	unknown function

CA0540	IPF10896	orf19.2821	0.5	NADH dehydrogenase (ubiquinone) (EC 1.6.5.3) 22K chain precursor (by homology)
CA5396	NAM7	orf19.939	0.5	nonsense-mediated mRNA decay protein (by homology)
CA1365	IPF11849	orf19.4459	0.5	unknown function
CA0161	RMS1	orf19.10177	0.5	(putative) transcriptional regulator (by homology)

9) Comparison of heat shock inducible, Hsf1-dependent genes in *C. albicans* with Hsf1-dependent genes in *S. cerevisiae* identified by ChIP

The *S. cerevisiae* ChIP data are from Hahn *et al.*, (2004) Mol Cell Biol 24: 5249-5256.

13 *C. albicans* Hsf1 induced genes have orthologues in the list of *S. cerevisiae* Hsf1 target genes identified by ChIP

HSP family genes					**HSEs**
CA1239	HSP60	Heat Shock Protein 60 (HSP60)	YLR259c ; HSP60 ; heat	orf19.717	
CA4683	HSP78	heat shock protein of clpb family of ATP-	YDR258c ; HSP78 ; hea	orf19.884	
CA4959	HSP90	heat shock protein	YMR186w ; HSC82 ; hea	orf19.6515	
CA5135	HSP104	Heat shock protein (by homology)	YLL026w ; HSP104 ; hea	orf19.13747	
CA0915	KAR2	dnaK-type molecular chaperone (by hon	YJL034w ; KAR2 ; nucle	orf19.9564	
CA4275	MDJ1	Heat shock protein - chaperone (by hom	YFL016c ; MDJ1 ; heat s	orf19.6672	
CA2854	RPN4	26S proteasome subunit (by homology)	YDL020c ; RPN4 ; 26S p	orf19.1069	
CA3098	SIS1	heat shock protein (by homology)	YNL007c ; SIS1 ; heat sh	orf19.3861	
CA2857	SSA1	Heat shock protein of HSP70 family	YER103w ; SSA4 ; heat	orf19.1065	
CA1230	SSA4	cahsp70 mRNA for heat shock	YER103w ; SSA4 ; heat	orf19.4980	
CA1911	SSE1	heat shock protein of HSP70 family (by h	YPL106c ; SSE1 ; heat s	orf19.2435	
Other stress induced genes					
CA6040	CPR6	cyclophylin (by homology)	YLR216c ; CPR6 ; meml	orf19.7654	
CA0265	STI1	stress-induced protein (by homology)	YOR027w ; STI1 ; stress	orf19.10702	

The *C. albicans* Hsf1 induced genes

CA2342	IPF13836	probable heat shock protein (by homolog	no significant match	orf19.2344
CA6059	CTA26	transcriptional activation	no significant match	orf19.7680
CA2317	IPF13586	unknown function	no significant match	orf19.5479
CA4757	LIP10	Secretory lipase	no significant match	orf19.4822
CA0037	IPF17652.3	reverse transcriptase, 3-prime end (by h	no significant match	orf19.6078
CA0602	CTA22	Protein with putative transcription activat	no significant match	orf19.3074
CA2752	IPF6238	GAG protein of retrotransposon pCal	no significant match	orf19.2374
CA2218	IPF18508	unknown function	no significant match	orf19.5375
CA5613	IPF525	unknown function	no significant match	orf19.7085
CA4221	IPF8760	unknown function	no significant match	orf19.823
CA2161	IPF11876	unknown function	no significant match	orf19.5295
CA3969	IPF9379	unknown function	no significant match	orf19.1430
CA3553	IFA16.5	unknown function, 5-prime end	no significant match	orf19.3878
CA1809	IPF6325	unknown function	no significant match	orf19.1116
CA4111	IPF3964	unknown function	no significant match	orf19.675
CA3803	IPF12407	unknown function	no significant match	orf19.2481

CA5552	IPF5971	unknown function	no significant match	orf19.7316
CA2562	IPF19953	unknown function	no significant match	orf19.5642
CA0611	IPF17542	unknown function	no significant match	orf19.13024
CA1168	IPF13017	unknown function	no significant match	orf19.1785
CA2316	IPF14542	unknown function	no significant match	orf19.8963
CA5266	IPF1787.3F	unknown function, 3-prime end	YBL029w ; ; hypothetica	orf19.4996
CA1908	APL3	AP-2 complex subunit, alpha-adaptin (by	YBL037w ; APL3 ; AP-2	orf19.2786
CA5339	IPF885	glucan 1,3-beta-glucosidase (by homolo	YBR056w ; ; similarity to	orf19.7214
CA0147	IPF9690	unknown function	YBR101c ; ; weak simila	orf19.11133
CA5325	IPF2138	unknown function	YBR137w ; ; hypothetica	orf19.7199
CA4602	IPF6231	unknown function	YCL033c ; ; similarity to	orf19.10802
CA0263	GLK1	aldohexose specific glucokinase (by hor	YCL040w ; GLK1 ; aldoh	orf19.1408
CA1137	IFN1	glycerophosphoinositol transporter (by h	YCR098c ; GIT1 ; glycer	orf19.1979
CA1138	IFN3	glycerophosphoinositol transporter (by h	YCR098c ; GIT1 ; glycer	orf19.1978
CA2854	RPN4	26S proteasome subunit (by homology)	YDL020c ; RPN4 ; 26S p	orf19.1069
CA1075	IPF4991	putative membrane protein	YDL058w ; USO1 ; intra	orf19.2531
CA2038	IPF17510	unknown function	YDL157c ; ; hypothetica	orf19.11836
CA4934	CDC36	transcription factor (by homology)	YDL165w ; CDC36 ; trar	orf19.6075
CA5846	IPF409	unknown function	YDL194w ; SNF3 ; high-	orf19.7502
CA4593	IPF2214	unknown function	YDR013w ; ; similarity to	orf19.6910
CA2895	IFS1	Unknown function	YDR014w ; RAD61 ; wea	orf19.2461
CA5029	IPF2997	unknown function	YDR028c ; REG1 ; regul	orf19.6983
CA5950	TPI1	Triose phosphate isomerase	YDR050c ; TPI1 ; triose-	orf19.6745
CA2173	IPF18527	unknown function	YDR108w ; GSG1 ; spor	orf19.3764
CA3379	IPF8350	putative methyltransferase (by homology	YDR140w ; FYV9 ; putat	orf19.331
CA0551	CDC37	Cell division control protein (by homolog	YDR168w ; CDC37 ; cell	orf19.5531
CA6002	IPF661	unknown function	YDR214w ; ; similarity to	orf19.7602
CA4683	HSP78.3F	heat shock protein of clpb family of ATP-	YDR258c ; HSP78 ; heat	orf19.884
CA4684	HSP78.5F	heat shock protein of clpb family of ATP-	YDR258c ; HSP78 ; heat	orf19.882
CA3367	IPF4667	unknown Function	YDR380w ; ARO10 ; sim	orf19.9405
CA2843	ALK5.5F	n-alkane-inducible cytochrome P-450, 5-	YDR402c ; DIT2 ; cytoch	orf19.5728
CA6054	IPF4952	unknown function	YDR411c ; ; weak simila	orf19.7672
CA2433	IPF12959	unknown function	YDR485c ; ; similarity to	orf19.3399
CA3551	IPF13229	unknown function	YDR520c ; ; weak simila	orf19.3876
CA0828	IPF17186	unknown function	YDR533c ; ; strong simi	orf19.7882
CA2361	IPF8950	unknown function	YER004w ; ; similarity to	orf19.12363
CA2857	SSA1	Heat shock protein of HSP70 family	YER103w ; SSA4 ; heat	orf19.1065
CA1230	SSA4	cahsp70 mRNA for heat shock	YER103w ; SSA4 ; heat	orf19.4980
CA4275	MDJ1	Heat shock protein - chaperone (by hom	YFL016c ; MDJ1 ; heat s	orf19.6672
CA2299	IPF8222	unknown function	YGL014w ; PUF4 ; simila	orf19.5381
CA1829	IPF17177.3F	similar to Saccharomyces cerevisiae Srr	YGL097w ; SRM1 ; GDP	orf19.5184
CA4437	IPF13867	unknown function	YGL145w ; TIP20 ; requi	orf19.5158

CA5848	IPF407	unknown function	YGR161c ; ; hypothetica	orf19.7504
CA6096	IPF56	similar to Saccharomyces cerevisiae Na	YGR232w ; NAS6 ; poss	orf19.5961
CA3405	IPF8644	maltase (by homology)	YGR287c ; ; strong simi	orf19.3982
CA1497	IPF9683	unknown function	YHR006w ; STP2 ; involv	orf19.3644
CA2756	IPF18418	unknown function	YHR029c ; ; similarity to	orf19.13065
CA1630	RPP1	required for processing of tRNA and 35S	YHR062c ; RPP1 ; requi	orf19.1029
CA5536	IPF2837	putative cystathionine gamma-synthase	YHR112c ; ; similarity to	orf19.7297
CA1399	IPF8069	unknown function	YHR121w ; ; weak simil	orf19.3698
CA3254	IPF4728	unknown Function	YHR172w ; SPC97 ; spir	orf19.11227
CA4227	SCH9	strong similarity to S.pombe sck1	YHR205w ; SCH9 ; stror	orf19.829
CA5602	IPF554	RNA binding protein (by homology)	YIR001c ; SGN1 ; mRNA	orf19.7097
CA3625	CYR1.3F	adenylate cyclase, 3-prime end	YJL005w ; CYR1 ; adeny	orf19.12617
CA0915	KAR2	dnaK-type molecular chaperone (by hom	YJL034w ; KAR2 ; nucle	orf19.9564
CA2594	IPF12824	unknown function	YJL072c ; ; hypothetical	orf19.57
CA5891	IPF2400	putative aldehyde reductase (by homolo	YJR096w ; ; similarity to	orf19.6816
CA5601	IPF556	transcriptional regulator (by homology)	YKL070w ; ; similarity to	orf19.7098
CA0896	SBA1	Hsp90 (Ninety) Associated Co-chaperon	YKL117w ; SBA1 ; Hsp9	orf19.5749
CA2130	RPS27A	ribosomal protein S27.e (by homology)	YKL156w ; RPS27A ; ribosomal protein S27	
CA5478	JEN2	carboxylic acid transporter protein (by ho	YKL217w ; JEN1 ; carbo	orf19.12767
CA4480	IPF6464	putative triacylglycerol lipase (by homolo	YLL012w ; ; similarity to	orf19.1887
CA3545	BPT1.5F	membrane transporter of the ATP-bindin	YLL015w ; BPT1 ; memb	orf19.6383
CA5135	HSP104	Heat shock protein (by homology)	YLL026w ; HSP104 ; hea	orf19.13747
CA3966	IPF9376	unknown function	YLR004c ; ; similarity to	orf19.1427
CA2474	PDC11	Pyruvate decarboxylase (by homology)	YLR044c ; PDC1 ; pyruv	orf19.2877
CA5264	IPF1798	unknown function	YLR098c ; CHA4 ; transc	orf19.4998
CA0150	CDC123	similar to Saccharomyces cerevisiae Cd	YLR215c ; CDC123 ; stro	orf19.10236
CA6040	CPR6	cyclophylin (by homology)	YLR216c ; CPR6 ; memb	orf19.7654
CA1822	IPF9525	unknown function	YLR222c ; ; similarity to	orf19.4268
CA1239	HSP60	Heat Shock Protein 60 (HSP60)	YLR259c ; HSP60 ; heat	orf19.717
CA3730	IPF11900	unknown function	YLR292c ; SEC72 ; ER p	orf19.4180
CA5847	CDA2	chitin deacetylase (by homology)	YLR308w ; CDA2 ; sporu	orf19.7503
CA1773	VRP1	verprolin (by homology)	YLR337c ; VRP1 ; verpro	orf19.2190
CA2338	NIT3	nitrilase (by homology)	YLR351c ; NIT3 ; nitrilas	orf19.2351
CA4739	IPF4498	unknown function	YMR071c ; ; hypothetica	orf19.4845
CA2039	IPF12947	unknown function	YMR115w ; ; similarity to	orf19.11835
CA3964	IPF9370	unknown function	YMR155w ; ; weak simil	orf19.1424
CA4473	IPF6447	unknown function	YMR172w ; HOT1 ; prote	orf19.1897
CA4959	HSP90	heat shock protein	YMR186w ; HSC82 ; hea	orf19.6515
CA6003	IPF660	unknown function	YMR244ca ; ; ; 3E-15 ;	orf19.7603
CA3886	IPF12963	ubiquitin-mediated protein degradation (	YMR275c ; BUL1 ; ubiqu	orf19.5094
CA4581	IPF2232	unknown function	YMR315w ; ; similarity to	orf19.6899
CA3098	SIS1	heat shock protein (by homology)	YNL007c ; SIS1 ; heat sl	orf19.3861

CA1795	IPF11261	unknown function	YNL041c ; ; weak simila	orf19.5209
CA3951	VAC7.3	Vacuolar protein, 3-prime end (by homol	YNL054w ; VAC7 ; vacu	orf19.1409
CA5120	YDJ1	Mitochondrial and ER import protein (by	YNL064c ; YDJ1 ; mitocl	orf19.6408
CA4502	IPF10391	Similar to dnaJ proteins	YNL064c ; YDJ1 ; mitocl	orf19.8136
CA5480	IPF8210	unknown function	YNL103w ; MET4 ; trans	orf19.5312
CA1966	IFR4	unknown function	YNL134c ; ; similarity to	orf19.2394
CA0821	IPF16795	glycerate/formate-dehydrogenase (by ho	YNL274c ; ; similarity to	orf19.2989
CA2434	IPF9484	unknown function	YNL281w ; HCH1 ; stron	orf19.3396
CA0796	ALR1	divalent cation transporter (by homology	YOL130w ; ALR1 ; divale	orf19.9175
CA2644	GRP2	Reductase (by homology)	YOL151w ; GRE2 ; simil	orf19.4309
CA0265	STI1	stress-induced protein (by homology)	YOR027w ; STI1 ; stress	orf19.10702
CA6128	IPF152	unknown function	YOR129c ; ; hypothetica	orf19.5919
CA1846	FDH12	Formate dehydrogenase (by homology)	YOR388c ; FDH1 ; stron	orf19.638
CA0924	IFD5	Putative aryl-alcohol dehydrogenase (by	YPL088w ; ; similarity to	orf19.1048
CA1911	SSE1	heat shock protein of HSP70 family (by I	YPL106c ; SSE1 ; heat s	orf19.2435
CA0169	RBT7	repressed by TUP1	YPL123c ; RNY1 ; simila	orf19.10196
CA5558	IPF1969	unknown function	YPL225w ; ; hypothetica	orf19.7322

The *S. cerevisiae* Hsf1 target genes from CHIP on Chip

ISNR190	YJL148W	unshared RNA polymerase I subunit	RPA34
ITD(GUC)B	YBR083W	transcription factor of the TEA/ATTS DNA-	TEC1
ITK(CUU)M	YMR106C	Exhibits DNA binding activity on its own	YKU80
ITR(ACG)O	YOR007C	small glutamine-rich tetratricopeptide	SGT2
iYAL004W	YAL005C	Heat shock protein of HSP70 family	SSA1
iYAL039C-1	YAL039C	cytochrome c heme lyase (CCHL)	CYC3
iYBL006C	YBL005W	Pleiotropic drug resistance protein 3	PDR3
iYBL075C	YBL075C	heat-inducible cytosolic member of the 70	SSA3
iYBR049C	YBR049C	RNA polymerase I enhancer binding	REB1
iYBR053C-	YBR054W	Homolog to HSP30 heat shock protein	YRO2
iYBR071W	YBR072W	heat shock protein 26	HSP26
iYBR082C-	YBR082C	ubiquitin-conjugating enzyme	UBC4
iYBR082C-	YBR082C	ubiquitin-conjugating enzyme	UBC4
iYBR085W-	YBR087W	Subunit 5 of Replication Factor C;	RFC5
iYBR101C	YBR101C	Hypothetical ORF	YBR101C
iYBR117C	YBR117C	transketolase	TKL2
iYBR157C-	YBR157C	Increased Copper Sensitivity	ICS2
iYBR169C	YBR169C	HSP70 family member	SSE2
iYCL050C	YCL050C	diadenosine 5'	APA1
iYCL051W	YCL051W		N/A
iYCR011C	YCR011C		N/A
iYCR021C	YCR021C	Protein induced by heat shock	HSP30
iYDL020C	YDL020C	ubiquitin-mediated 26S proteasome	RPN4

iYDL037C-	YDL037C	Hypothetical ORF	YDL037C
iYDL084W	YDL082W	Ribosomal protein L13A	RPL13A
iYDL194W	YDL193W	Protein required for cell viability	YDL193W
iYDL197C	YDL197C	Anti-silencing protein	ASF2
iYDR002W	YDR003W	Hypothetical ORF	YDR003W
iYDR010C	YDR011W	ABC transporter	SNQ2
iYDR108W	YDR110W	DNA replication fork blocking protein	FOB1
iYDR151C	YDR151C	member of the CCCH zinc finger protein	CTH1
iYDR155C	YDR155C	cyclophilin peptidyl-prolyl cis-trans	CPR1
iYDR170W-	YDR171W	heat shock protein similar to HSP26	HSP42
iYDR184C	YDR184C	nuclear protein that interacts with Aip3	ATC1
iYDR208W	YDR210W	Hypothetical ORF	YDR210W
iYDR213W	YDR214W	Hypothetical ORF	AHA1
iYDR258C	YDR258C	Mitochondrial heat shock protein 78 kDa	HSP78
iYDR313C	YDR313C	phosphatidylinositol(3)-phosphate binding	PIB1
iYER034W	YER035W	Functions with Edc1p to stimulate mRNA	EDC2
iYER036C	YER037W	involved in phosphate metabolism	PHM8
iYER045C-	YER046W	Hypothetical ORF	SPO73
iYER102W	YER103W	member of 70 kDa heat shock protein	SSA4
iYFL015C	YFL014W	12 kDa heat shock protein	HSP12
iYFL016C	YFL014W	12 kDa heat shock protein	HSP12
iYFL039C	YFL039C	Actin	ACT1
iYGL007W-	YGL006W	putative vacuolar Ca2+ ATPase	PMC1
iYGL008C	YGL006W	putative vacuolar Ca2+ ATPase	PMC1
iYGL037C	YGL037C	pyrazinamidase and nicotinamidase	PNC1
iYGL070C	YGL068W	Protein required for cell viability	YGL068W
iYGR141W	YGR142W	Gene/protein whose expression is elevated	BTN2
iYGR146C	YGR146C	Hypothetical ORF	YGR146C
iYGR161C-	YGR161C	Hypothetical ORF	YGR161C
iYGR192C	YGR192C	Glyceraldehyde-3-phosphate	TDH3
iYGR197C	YGR197C	involved in nitrosoguanidine resistance	SNG1
iYGR210C	YGR211W	zinc finger protein	ZPR1
iYGR250C-	YGR250C	Hypothetical ORF	YGR250C
iYGR284C	YGR284C	ER-Golgi transport vesicle protein	ERV29
iYHR048W	YHR049W		FSH1
iYHR053C	YHR053C	copper-binding metallothionein	CUP1-1
iYHR055C	YHR055C	copper-binding metallothionein	CUP1-2
iYHR082C	YHR082C	Ser/Thr protein kinase	KSP1
iYHR103W	YHR104W	a keto-aldose reductase	GRE3
iYHR107C	YHR107C	Component of 10 nm filaments of mother-	CDC12
iYHR173C	YHR174W	enolase	ENO2
iYIL109C	YIL108W	Hypothetical ORF	YIL108W

iYIR017C	YIR017C	transcriptional activator in the Cbf1p-Met4p-	MET28
iYIR018W-	YIR018W-2		N/A
iYIRCDELT	YIRCdelta6	Ty1 LTR	YIRCdelta6
iYJL035C	YJL034W	Homologue of mammalian BiP (GPR78)	KAR2
iYJL074C	YJL074C	SMC chromosomal ATPase family member	SMC3
iYJL116C	YJL116C	involved in regulating expression of F0F1	NCA3
iYJL145W	YJL144W	Hypothetical ORF	YJL144W
iYJR045C	YJR046W	Product of gene unknown	TAH11
iYJR056C	YJR056C	Hypothetical ORF	YJR056C
iYJR103W	YJR105W	adenosine kinase	ADO1
iYJR116W	YJR117W	zinc metallo-protease that catalyzes the	STE24
iYKL010C	YKL010C	involved in ubiqitin-mediated protein	UFD4
iYKL038W	YKL035W	Uridinephosphoglucose	UGP1
iYKL052C	YKL051W	Hypothetical ORF	YKL051W
iYKL097W-	YKL096W	cell wall mannoprotein	CWP1
iYKL110C-0	YKL109W	transcriptional activator protein of CYC1	HAP4
iYKL164C-0	YKL163W	Protein containing tandem internal repeats	PIR3
iYKR071C	YKR074W	Hypothetical ORF	YKR074W
iYLL024C	YLL024C	member of 70 kDa heat shock protein	SSA2
iYLL027W	YLL026W	104 kDa heat shock protein	HSP104
iYLR063W	YLR064W	Hypothetical ORF	YLR064W
iYLR108C	YLR109W	alkyl hydroperoxide reductase	AHP1
iYLR217W	YLR216C	cyclophilin related to the mammalian CyP-	CPR6
iYLR218C	YLR219W		MSC3
iYLR259C	YLR259C	mitochondrial chaperonin	HSP60
iYLR327C	YLR327C	Hypothetical ORF	YLR327C
iYLR347C	YLR350W	Endoplasmic reticulum membrane-	ORM2
iYML101C	YML100W	123 kD regulatory subunit of trehalose-6-	TSL1
iYML130C	YML130C	involved in protein disulfide bond formation	ERO1
iYMR068W	YMR069W	Hypothetical ORF	YMR069W
iYMR107W	YMR108W	acetolactate synthase	ILV2
iYMR160W	YMR161W	similar to E. coli DnaJ	HLJ1
iYMR185W	YMR186W	constitutively expressed heat shock protein	HSC82
iYMR210W	YMR211W	YMR211W	DML1
iYMR251W	YMR251W-A	hyperosmolarity-responsive gene	HOR7
iYNL007C	YNL007C	sit4 suppressor	SIS1
iYNL064C	YNL064C	yeast dnaJ homolog (nuclear envelope	YDJ1
iYNL078W	YNL077W	HSP40 family chaperone	YNL077W
iYNL125C	YNL125C	Putative monocarboxylate permease	ESBP6
iYNL194C	YNL194C	Hypothetical ORF	YNL194C
iYNL281W	YNL279W	pheromone-regulated membrane protein	PRM1
iYNL282W	YNL281W	high copy Hsp90 supressor	HCH1

iYNR033W	YNR034W	shows similarity to glucose-6-phosphate	SOL1
iYNR069C-	YNR069C	Hypothetical ORF	YNR069C
iYOL033W	YOL032W	Hypothetical ORF	YOL032W
iYOL052C	YOL051W	Component of the RNA polymerase II	GAL11
iYOL082W	YOL081W	encodes a GTPase activating protein	IRA2
iYOL110W	YOL109W	Overexpression causes resistance to	YOL109W
iYOR007C	YOR007C	small glutamine-rich tetratricopeptide	SGT2
iYOR019W	YOR019W		N/A
iYOR020C-	YOR020C	10 kDa mitochondrial heat shock protein	HSP10
iYOR025W	YOR026W	cell cycle checkpoint protein	BUB3
iYOR026W	YOR027W	Heat shock protein also induced by	STI1
iYOR152C-	YOR152C	Hypothetical ORF	YOR152C
iYOR267C	YOR267C	Hypothetical ORF	HRK1
iYOR298C-	YOR299W	involved in bipolar bud site selection	BUD7
iYOR298W	YOR298W		N/A
iYOR344C-	YOR344C	TYE7	TYE7
iYOR344C-	YOR344C	TYE7	TYE7
iYPL106C	YPL106C	HSP70 family member	SSE1
iYPL240C	YPL240C	82 kDa heat shock protein; homolog of	HSP82
iYPL250C	YPL250C	interacting with the cytoskeleton	ICY2
iYPL251W	YPL252C	iron-sulfur protein homologous to human	YAH1
iYPR148C-	YPR149W	Involved in secretion of proteins that lack	NCE102
iYPR157W	YPR158W	Hypothetical ORF	YPR158W
iYPR174C	YPR174C	Hypothetical ORF	YPR174C
YBL074C	YBL075C	heat-inducible cytosolic member of the 70	SSA3
YBR050C	YBR050C	putative Glc7 regulatory subunit	REG2
YBR071W	YBR071W	Hypothetical ORF	YBR071W
YBR082C	YBR082C	ubiquitin-conjugating enzyme	UBC4
YBR101C	YBR101C	Hypothetical ORF	YBR101C
YCL050C	YCL050C	diadenosine 5'	APA1
YCR012W	YCR011C	Active transport ATPase	ADP1
YCR022C	YCR021C	Protein induced by heat shock	HSP30
YDL020C	YDL020C	ubiquitin-mediated 26S proteasome	RPN4
YDR003W	YDR003W	Hypothetical ORF	YDR003W
YDR010C	YDR011W	ABC transporter	SNQ2
YDR151C	YDR151C	member of the CCCH zinc finger protein	CTH1
YDR156W	YDR155C	cyclophilin peptidyl-prolyl cis-trans	CPR1
YDR210W	YDR210W	Hypothetical ORF	YDR210W
YDR213W	YDR214W	Hypothetical ORF	AHA1
YDR214W	YDR214W	Hypothetical ORF	AHA1
YDR215C	YDR216W	positive transcriptional regulator of ADH2	ADR1
YDR231C	YDR231C	protein required for maturation and	COX20

YDR246W	YDR247W	Hypothetical ORF	YDR247W
YDR259C	YDR258C	Mitochondrial heat shock protein 78 kDa	HSP78
YER034W	YER033C	zinc regulated gene	ZRG8
YER035W	YER035W	Functions with Edc1p to stimulate mRNA	EDC2
YER057C	YER057C	heat-regulated protein	HMF1
YER103W	YER103W	member of 70 kDa heat shock protein	SSA4
YER150W	YER150W	similar to Sed1; highly expressed in	SPI1
YFL015C	YFL014W	12 kDa heat shock protein	HSP12
YFL016C	YFL016C	DnaJ homolog involved in mitochondrial	MDJ1
YGL007W	YGL006W	putative vacuolar Ca2+ ATPase	PMC1
YGL069C	YGL068W	Protein required for cell viability	YGL068W
YGR141W	YGR142W	Gene/protein whose expression is elevated	BTN2
YGR210C	YGR210C	Hypothetical ORF	YGR210C
YGR211W	YGR211W	zinc finger protein	ZPR1
YGR285C	YGR284C	ER-Golgi transport vesicle protein	ERV29
YHR053C	YHR053C	copper-binding metallothionein	CUP1-1
YHR054C	YHR054C	Hypothetical ORF	YHR054C
YHR055C	YHR054C	Hypothetical ORF	YHR054C
YHR156C	YHR156C	Hypothetical ORF	LIN1
YHR162W	YHR161C	clathrin assembly protein	YAP1801
YHR173C	YHR174W	enolase	ENO2
YJL144W	YJL144W	Hypothetical ORF	YJL144W
YJL145W	YJL144W	Hypothetical ORF	YJL144W
YJR046W	YJR046W	Product of gene unknown	TAH11
YKL031W	YKL032C	intrastrand crosslink recognition protein	IXR1
YKL036C	YKL035W	Uridinephosphoglucose	UGP1
YKL037W	YKL035W	Uridinephosphoglucose	UGP1
YLL023C	YLL024C	member of 70 kDa heat shock protein	SSA2
YLL027W	YLL026W	104 kDa heat shock protein	HSP104
YLL038C	YLL039C	ubiquitin	UBI4
YLR064W	YLR064W	Hypothetical ORF	YLR064W
YLR168C	YLR168C	possibly involved in intramitochondrial	YLR168C
YLR170C	YLR168C	possibly involved in intramitochondrial	YLR168C
YLR171W	YLR170C	clathrin-associated protein complex	APS1
YLR216C	YLR216C	cyclophilin related to the mammalian CyP-	CPR6
YLR217W	YLR216C	cyclophilin related to the mammalian CyP-	CPR6
YLR218C	YLR219W		MSC3
YLR327C	YLR327C	Hypothetical ORF	YLR327C
YLR379W	YLR378C	membrane component of ER protein	SEC61
YML101C	YML101C	Hypothetical ORF	CUE4
YMR069W	YMR070W	2 Cys2-His2 zinc fingers at c-terminus	MOT3
YMR185W	YMR186W	constitutively expressed heat shock protein	HSC82

YMR186W	YMR186W	constitutively expressed heat shock protein	HSC82
YMR210W	YMR210W	Hypothetical ORF	YMR210W
YNL006W	YNL007C	sit4 suppressor	SIS1
YNL063W	YNL064C	yeast dnaJ homolog (nuclear envelope	YDJ1
YNL064C	YNL064C	yeast dnaJ homolog (nuclear envelope	YDJ1
YNL083W	YNL084C	Required for endocytosis and organization	END3
YNL281W	YNL281W	high copy Hsp90 supressor	HCH1
YOR020C	YOR020C	10 kDa mitochondrial heat shock protein	HSP10
YOR026W	YOR027W	Heat shock protein also induced by	STI1
YOR027W	YOR027W	Heat shock protein also induced by	STI1
YOR203W	YOR201C	Ribose methyltransferase for mitochondrial	PET56
YOR267C	YOR267C	Hypothetical ORF	HRK1
YOR268C	YOR267C	Hypothetical ORF	HRK1
YOR298C-	YOR299W	involved in bipolar bud site selection	BUD7
YPL054W	YPL054W	Product of gene unknown	LEE1
YPL106C	YPL106C	HSP70 family member	SSE1
YPL170W	YPL170W	Hypothetical ORF	YPL170W
YPL239W	YPL240C	82 kDa heat shock protein; homolog of	HSP82
YPL250C	YPL250C	interacting with the cytoskeleton	ICY2

10) Impact of Temperature and Growth Rate upon HSP gene expression in *S. cerevisiae*

Expression data on *S. cerevisiae HSP* mRNA levels were extracted from the following datasets.
The mean fold changes for these *HSP* transcripts were then calculated and plotted

1. Gasch *et al.* (2000) *Mol Biol Cell* 11: 4241-4257
2. Regenberg *et al.* (2006) *Genome Biol* 7: R107

S. cerevisiae data

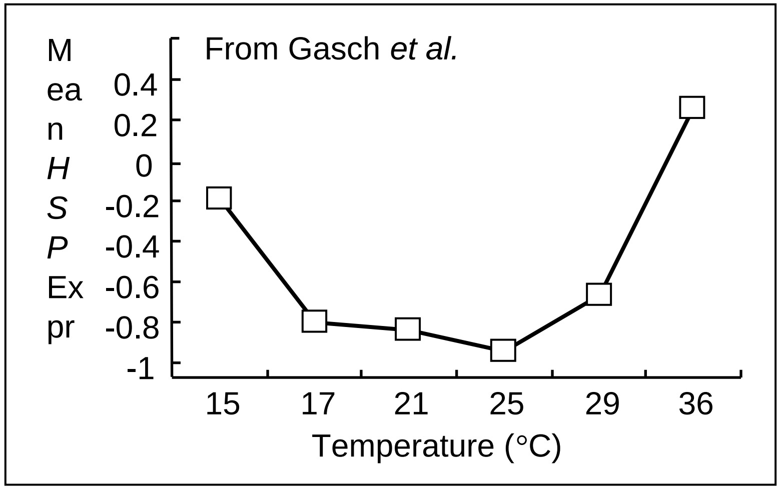

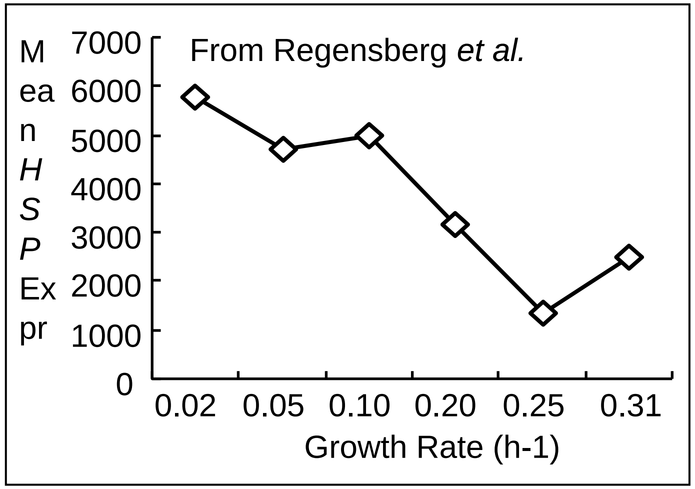

C. albicans data (from this study)

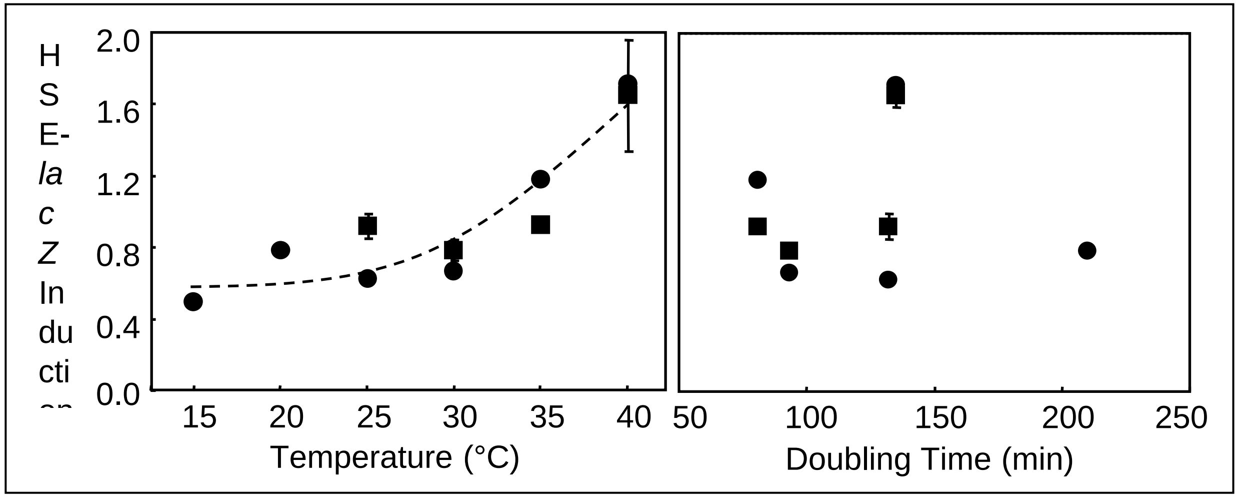

11) Oligonucleotides used in this study

Primer	Sequence (5’ to 3’)	Use
RPS1-GEN	GTGTGGGATTAAGTGAATACG	Diagnosis of insertion of CIp10-based plasmids at [illegible]
TET-F	TTTTACTAGTTGGACTTCTTCGCCAGAGG	Diagnosis of *tet-HSF1* allele
TET-R	TTTTACTAGTACTAGTTTTCTGAGATAAAGCTGTTTTT	Diagnosis of *tet-HSF1* allele
HSF1-5DR	CATCTCGAGATATTTCTTCCTCAACAACCTCATTAACAGTTTCTATATTTAT ATTCGTTTTTGTATGATTATGAATATGACTACTGATTATAGAGATCCAGTTT TCCCAGTCACGACGTT	PCR-amplification of *hsf*::*hisG-URA3-hisG* cassette
HSF1-3DR	ATAATGAATTGAAAAATATAATGATTATAGACACACTTCATCTATATCCTAT TTAATGATCGCTAACTTCTTCGATTGATCTTTTCTTCAGTTTCTTCTCTGTG GAATTGTGAGCGGATA	PCR-amplification of *hsf*::*hisG-URA3-hisG* cassette
Ura3-diag	CGTAGATTATTTTGTTGAAA	Diagnosis of *URA3* allele
HSF-F	GTTTGTGGCACTGACAGA	Diagnosis of *HSF1* allele
HSF-Diag	GACTGTTATTAGCTGGGC	Diagnosis of *HSF1* allele
HSF1-TET-5’	AAACATCACGAAGAGGTTAATCATTAGTTTAGACTTTTTCAAATTTTCATCC TTAATTTAACATCTCGAGATATTTCTTCCTCAACAACCTCATTAACAGGTAA TACGACTCACTATAGGG	PCR-amplification of *URA3-tet-HSF1* cassette
HSF1-TET-3’	TGGGAGATGATGTTTCATTGCCAGAGTTAGACTCTGTACCAAACAAATCGAG TAATGGATCTCTATAATCAGTAGTCATATTCATAATCATACAAAAACGCTAG TTTTCTGAGATAAAGCTG	PCR-amplification of *URA3-tet-HSF1* cassette
ACT1-F	GATGAAGCCCAATCCAAAAG	PCR-amplification of *ACT1* probe
ACT1-R	GGAGTTGAAAGTGGTTTGGT	PCR-amplification of *ACT1* probe
HSP90-F	TAGTCGACTATGGCTGACGCAAAAGTTG	PCR-amplification of *HSP90* probe
HSP90-R	ACATGGTACCACGACCCAAT	PCR-amplification of *HSP90* probe
HSP104-F	TTGCTGCATTTATCCCATCA	PCR-amplification of *HSP104* probe
HSP104-R	CAGCATCACCAATCAACACC	PCR-amplification of *HSP104* probe
HSP70-F	TGATGCTGCCAAGAATCAAG	PCR-amplification of *HSP70* probe
HSP70-R	TCACCAGCAGTGGCTTTAACT	PCR-amplification of *HSP70* probe
LacZ-F	GCTTCAAGGTTTTGGTTCTCC	PCR-amplification of *lacZ* probe
LacZ-R	GGACTTTCAGCACTCCAAGG	PCR-amplification of *lacZ* probe
HSP104p-F	TAGTCGACTGTAAGGAATATACTGTG	Cloning of *HSP104* promoter
HSP104p-R	ATAGTGACGATCTAATAGTGTATAATTG	Cloning of *HSP104* promoter

HSE-F	TCGACATTCGAGAATATTCCAGAATG	Cloning of standard HSE elements
HSE-R	TCGACATTCTGGAATATTCTCGAATG	Cloning of standard HSE elements
nHSE-F	TCGACAGAAGTCATTAGAATCGCTAAGAATG	Cloning of nonstandard HSE elements
nHSE-R	TCGACATTCTTAGCGATTCTAATGACTTCTG	Cloning of nonstandard HSE elements
FLAG-F	AGCTACGGATCCACCATGGATTACAAAGATGATGATGATAAAGGTGGTGATT ACAAAGATGATGATGATAAAGGTGGTGGTGATTACAAAGATGATGATGATAA AGGTGGTTTA	Cloning of FLAG tag
FLAG-R	AGCTTAAACCACCTTTATCATCATCATCTTTGTAATCACCACCACCTTTATC ATCATCATCTTTGTAATCACCACCTTTATCATCATCATCTTTGTAATCCATG GTGGATCCGT	Cloning of FLAG tag
ACT1-F	GCTGAACGTATGCAAAAG	Real time RT-PCR of *ACT1* transcript levels
ACT1-R	GAACAATGGATGGACCAG	Real time RT-PCR of A*CT1* transcript levels
CTA1-F	GGCCCATTTCGATAGAGA	Real time RT-PCR of *CTA1* transcript levels
CTA1-R	AAACACCATAAGCACCGG	Real time RT-PCR of *CTA1* transcript levels
EFB1-F	CCAAAACCAGCTGCCAAA	Real time RT-PCR of *EFB1* transcript levels
EFB1-R	GGAATCCATTGGTGAGCA	Real time RT-PCR of *EFB1* transcript levels
HSP90-F	CTGGTGCTGACGTTTCTA	Real time RT-PCR of *HSP90* transcript levels
HSP90-R	ACCAGCGTTAGATTCCCA	Real time RT-PCR of *HSP90* transcript levels
PGA23-F	GCCGGTATTGCAAACACT	Real time RT-PCR of *PGA23* transcript levels
PGA23-R	ACCAACAGCACCAGAAGT	Real time RT-PCR of *PGA23* transcript levels
